# Supplementary material for: Enalaprilat reverses neutrophil polarization imbalance via targeting taurine-STING axis for treatment of diabetic wounds
Source: Cell Rep Med. 2026 Mar 30;7(4):102714. doi: 10.1016/j.xcrm.2026.102714 (PMC13130670; doi:10.1016/j.xcrm.2026.102714)
Supplement: Document S1. Figures S1–S13 and Tables S1–S4 [file mmc1.pdf]

Cell Reports Medicine, Volume *7*

Supplemental information

Enalaprilat reverses neutrophil polarization imbalance via targeting taurine-STING axis for treatment of diabetic wounds

Li Lu, Yuan Xiong, Jiewen Liao, Juan Zhou, Guangji Wang, Yating Qin, Shengming Zhang, Yanzhi Zhao, Xiaodan Zhong, Mengwen Wang, Kangkang Zha, Fawwaz Al-Smadi, Guohui Liu, Yanli Zhao, and Bobin Mi

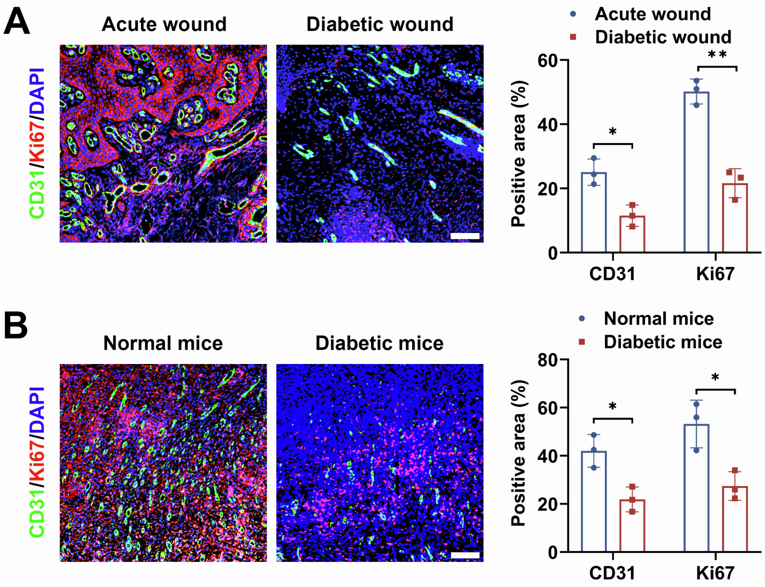

Figure S1. New vessel formation in the wound area of clinical samples and animal samples. Related to Figure 1.

(A) Representative immunofluorescence staining images of CD31 and Ki67 levels in clinical specimens of diabetic wounds and acute wounds. Scale bar: 100 μm; n = 3 biologically independent samples.

(B) Representative immunofluorescence staining images of CD31 and Ki67 levels in murine diabetic wounds and acute wounds. Scale bar: 200 μm; n = 3 biologically independent samples.

Data were shown as mean ± standard deviation (S.D.) from biological replicates and statistical comparisons were performed using unpaired Student's t test in A and B. *$P < 0.05$, **$P < 0.01$.

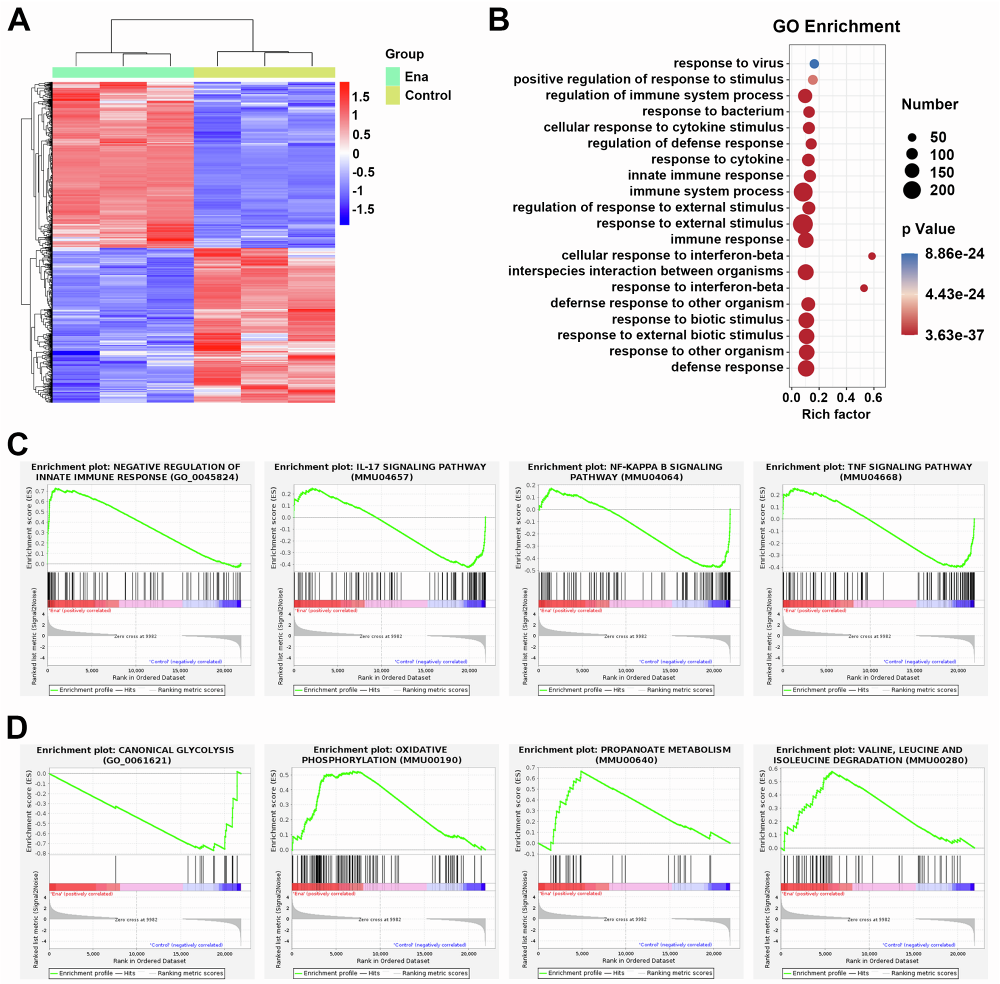

Figure S2. High-throughput RNA sequencing performed on AGE-stimulated BMDNs with or without Ena treatment. Related to Figure 3.

(A) Clustering analysis plot.

(B) Top 20 GO terms enriched by differentially expressed genes.

(C) Pathways involved in immune regulation shown by GESA.

(D) GESA revealing metabolic pathway including glycolysis, oxidative phosphorylation, propanoate metabolism as well as degradation of valine, leucine, and isoleucine.

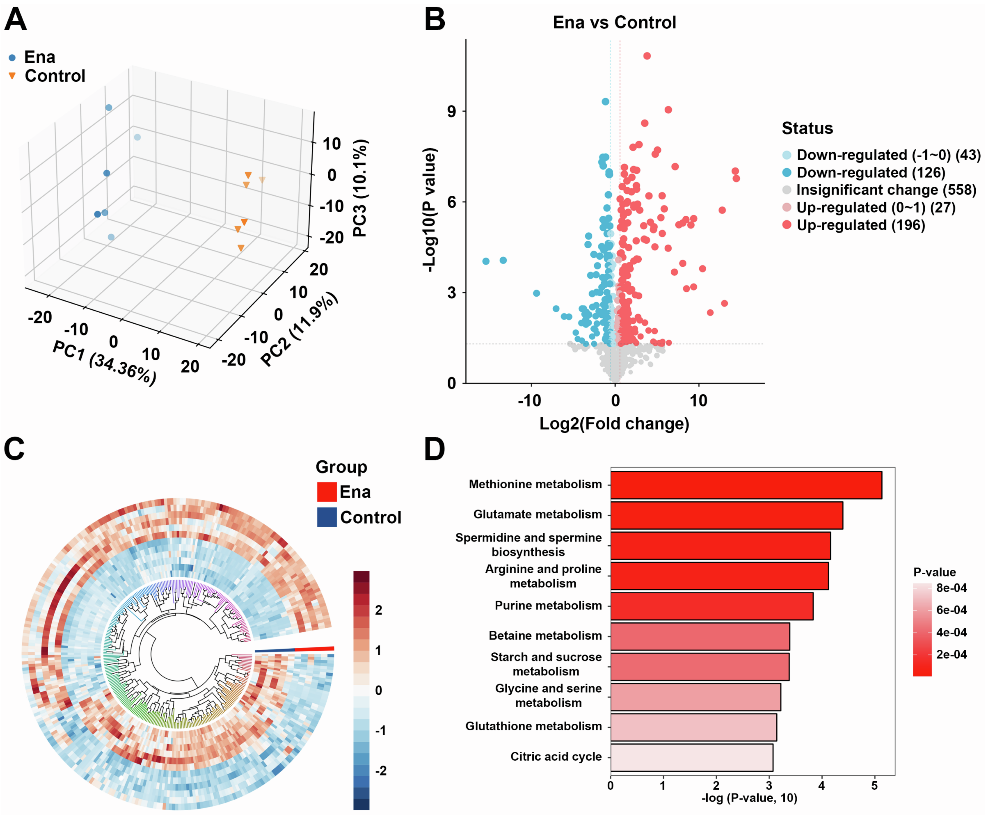

Figure S3. Metabolomics analysis performed on AGE-stimulated BMDNs with or without Ena treatment. Related to Figure 3.

(A) Visualization of PCA result.

(B) Volcano plot.

(C) Hierarchical clustering of differentially accumulated metabolites after the treatment of Ena.

(D) Small Molecule Pathway Database analysis showing significant pathways enriched by differentially accumulated metabolites.

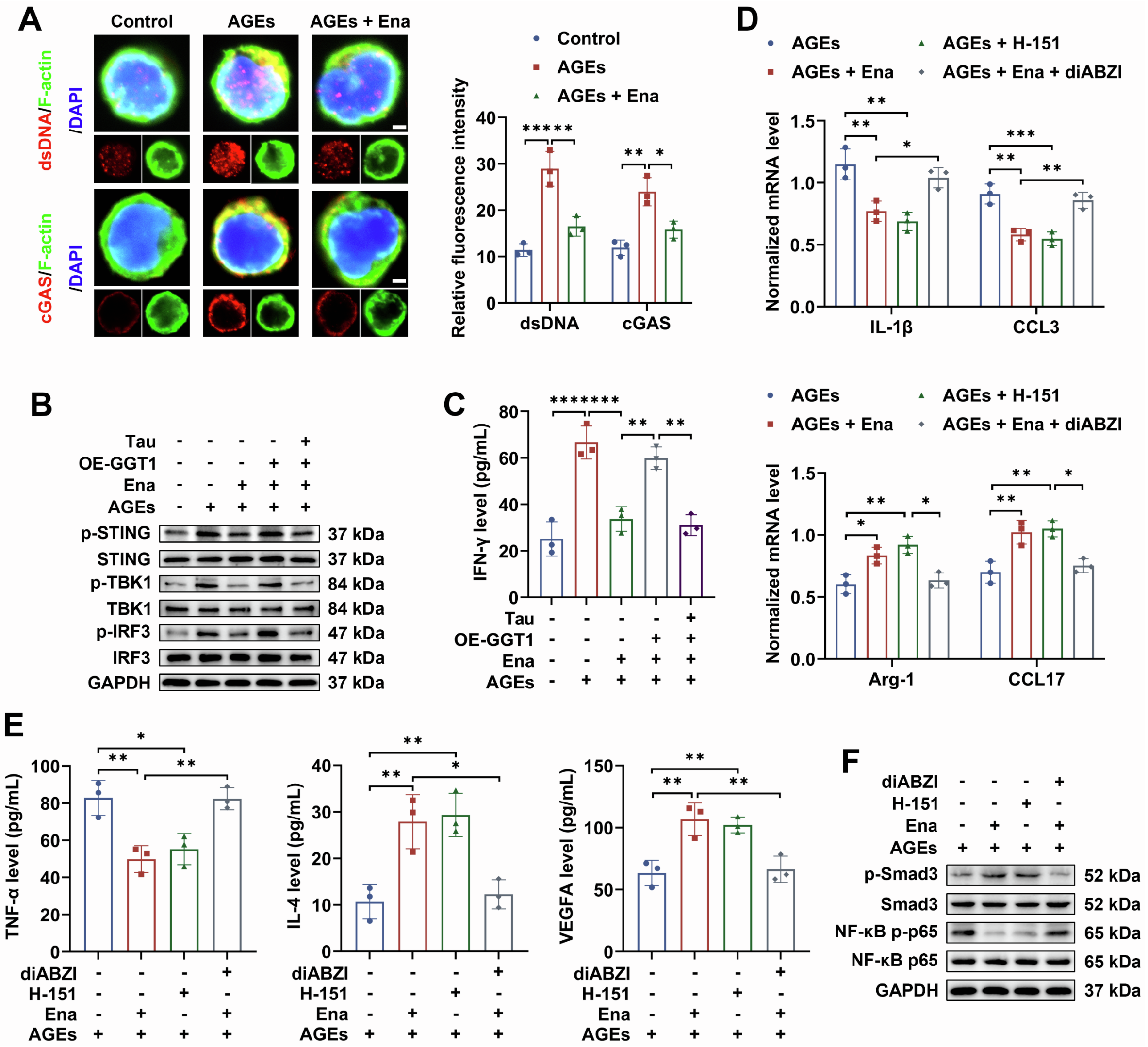

Figure S4. Ena facilitated phenotype repolarization via taurine-dependent cGAS-STING pathway inhibition. Related to Figure 4.

(A) Immunofluorescence staining analysis of dsDNA and cGAS in BMDNs with different treatments. Scale bar: 2 μm; n = 3 independent experiments.

(B) Western blot analysis of STING, TBK1 and IRF3 activity in neutrophils with indicated treatments; n = 3 independent experiments.

(C) Levels of IFN-γ released by neutrophils as measured by ELISA reagent. n = 3 independent experiments.

(D) qRT-PCR was employed to measure the expression of *il1b*, *ccl3*, *arg1* and *ccl17* in neutrophils. n = 3 independent experiments.

(E) Levels of TNF-α, IL-4 and VEGFA generated by neutrophils were detected by ELISA kits. n = 3 independent experiments.

(F) Western blot was adopted to determine the activities of Smad3 and NF-κB p65 in neutrophils. n = 3 independent experiments.

Data were shown as mean ± standard deviation (S.D.) from biological replicates and statistical comparisons were performed using one-way ANOVA followed by Tukey's multiple comparisons test in (A-F). *$P < 0.05$, **$P < 0.01$, ***$P < 0.001$, ****$P < 0.0001$.

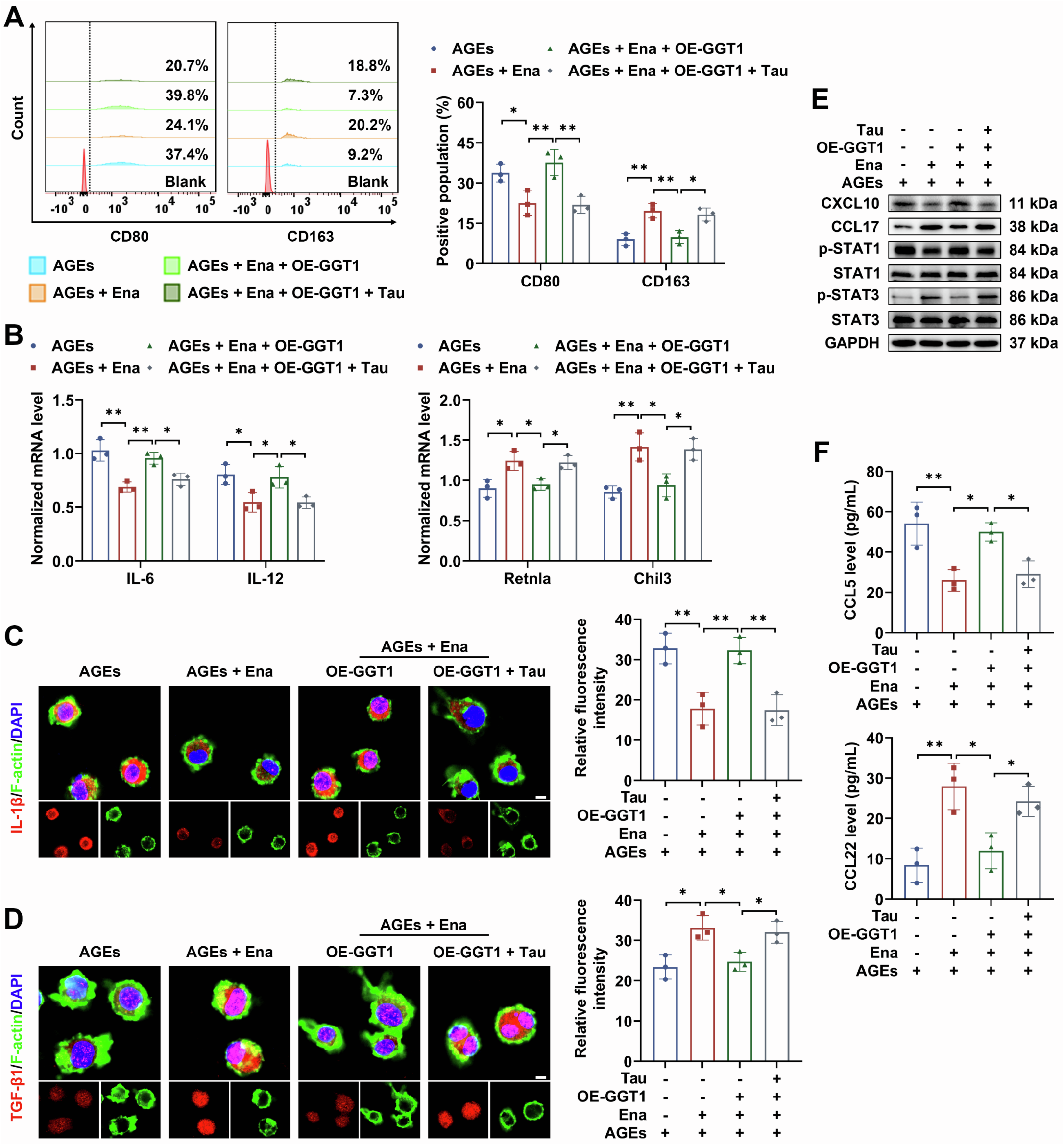

Figure S5. Regulator roles produced by Ena-treated neutrophils in the inflammation alleviation of macrophages. Related to Figure 4.

(A) Population of CD80+ or CD163+ BMDMs as evaluated by flow cytometry. n = 3 independent experiments.

(B) mRNA levels of *il6*, *il12*, *retnla* and *chil3* in BMDMs incubated with neutrophils pretreated by different approaches as measured by qRT-PCR. n = 3 independent experiments.

(C and D) Representative immunofluorescence staining of IL-1β and TGF-β1 in BMDMs affected by neutrophils with indicated preconditioning. Scale bar: 5 μm; n = 3 independent experiments.

(E) Western blot analysis of CXCL10, CCL17, STAT1 and STAT3 in macrophages. n = 3 independent experiments.

(F) Levels of CCL5 and CCL22 in BMDMs as measured by ELISA kits. n = 3 independent experiments.

Data were shown as mean ± standard deviation (S.D.) from biological replicates and statistical comparisons were performed using one-way ANOVA followed by Tukey's multiple comparisons test in (A-F). $*P < 0.05$, $**P < 0.01$.

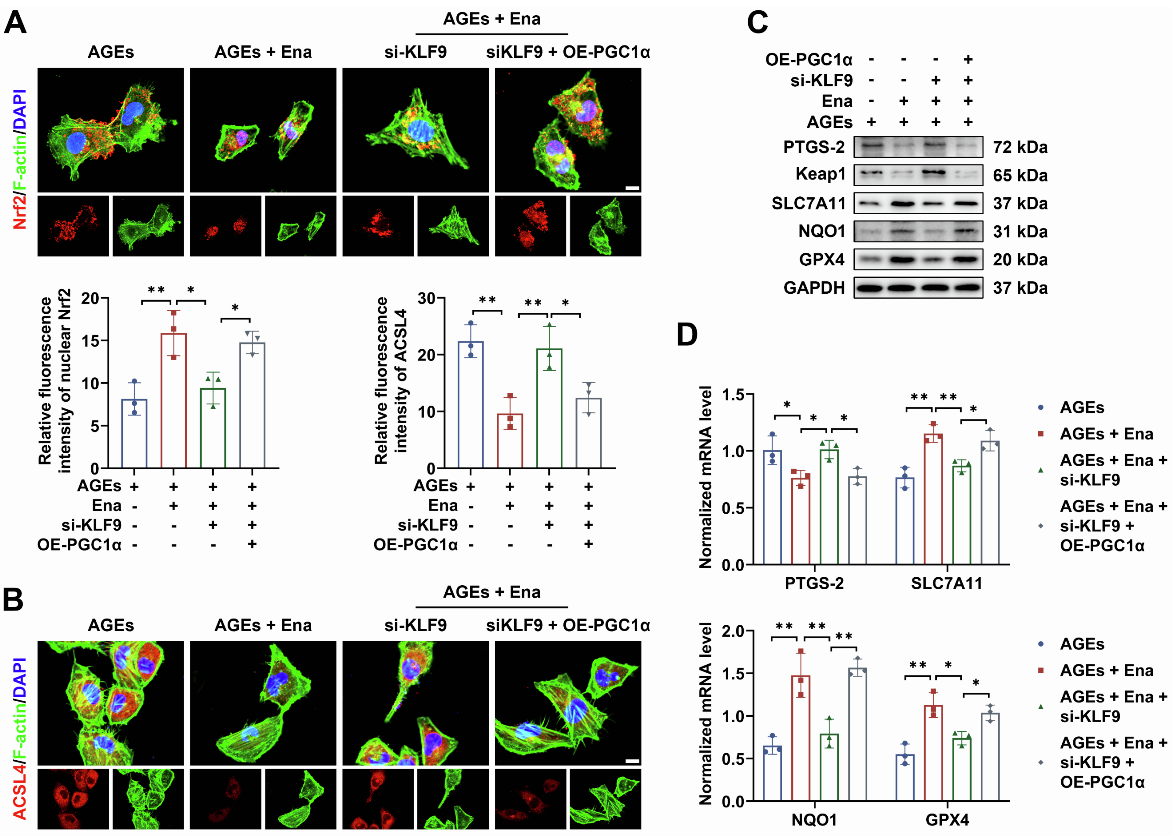

Figure S6. Mechanisms by which Ena-treated neutrophils repressed HUVEC ferroptosis. Related to Figure 5.

(A and B) Immunofluorescence analysis was employed to detect Nrf2 and ACSL4 expression in pretreated HUVECs co-cultured with AGEs and Ena-treated neutrophils. Scale bar: 10 μm; n = 3 independent experiments.

(C) Western blot analysis of PTGS-2, Keap1, SLC7A11, NQO1 and GPX4 in HUVECs. n = 3 independent experiments.

(D) mRNA levels of *ptgs2*, *slc7a11*, *nqo1* and *gpx4* in HUVECs measured by qRT-PCR. n = 3 independent experiments.

Data were shown as mean ± standard deviation (S.D.) from biological replicates and statistical comparisons were performed using one-way ANOVA followed by Tukey's multiple comparisons test in (A-D). $^{*}P < 0.05$, $^{**}P < 0.01$.

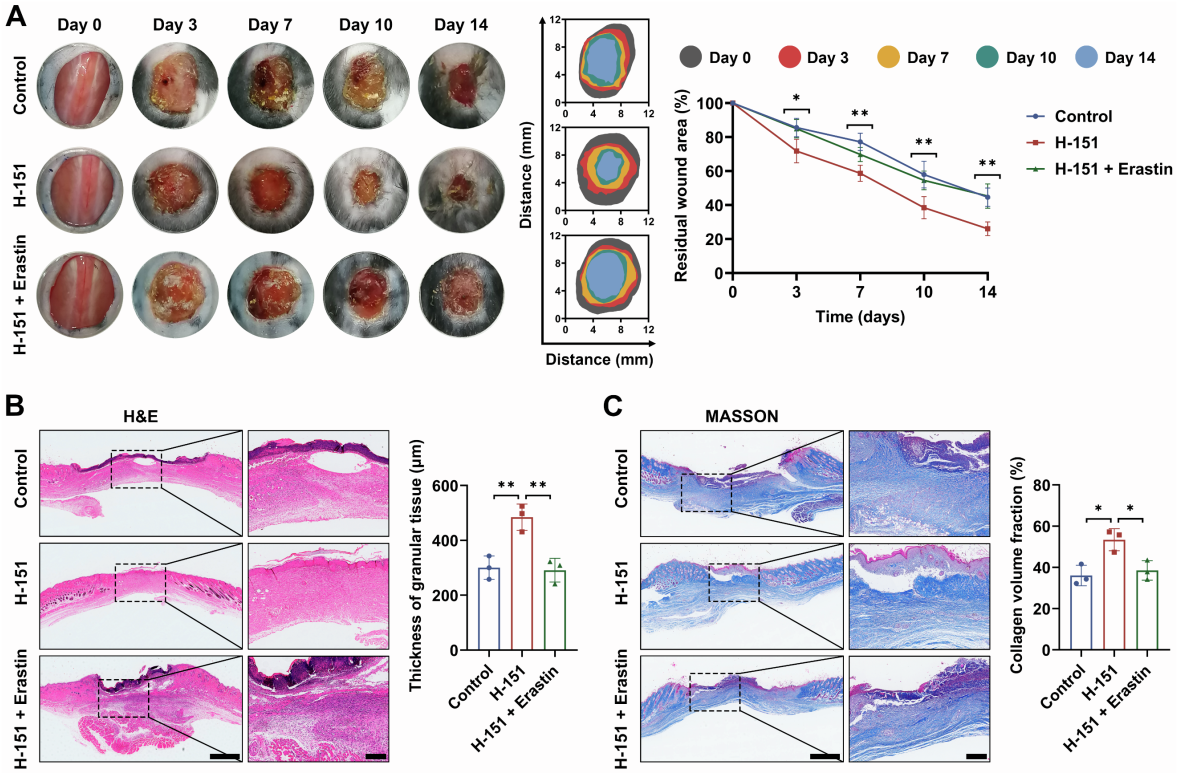

Figure S7. Effect of STING-ferroptosis axis on the healing process of diabetic wounds. Related to Figure 5.

(A) Representative images of wound area during the healing course. n = 4 mice per group.

(B) Histological analysis via H&E staining. Scale bar: 1 mm (left) and 250 μm (right); n = 3 biologically independent samples.

(C) Tissue collagen content visualized by Masson's trichrome staining. Scale bar: 1 mm (left) and 250 μm (right); n = 3 biologically independent samples.

Data were shown as mean ± standard deviation (S.D.) from biological replicates and statistical comparisons were performed using one-way ANOVA followed by Tukey's multiple comparisons test in (A-C). $^{*}P < 0.05$, $^{**}P < 0.01$.

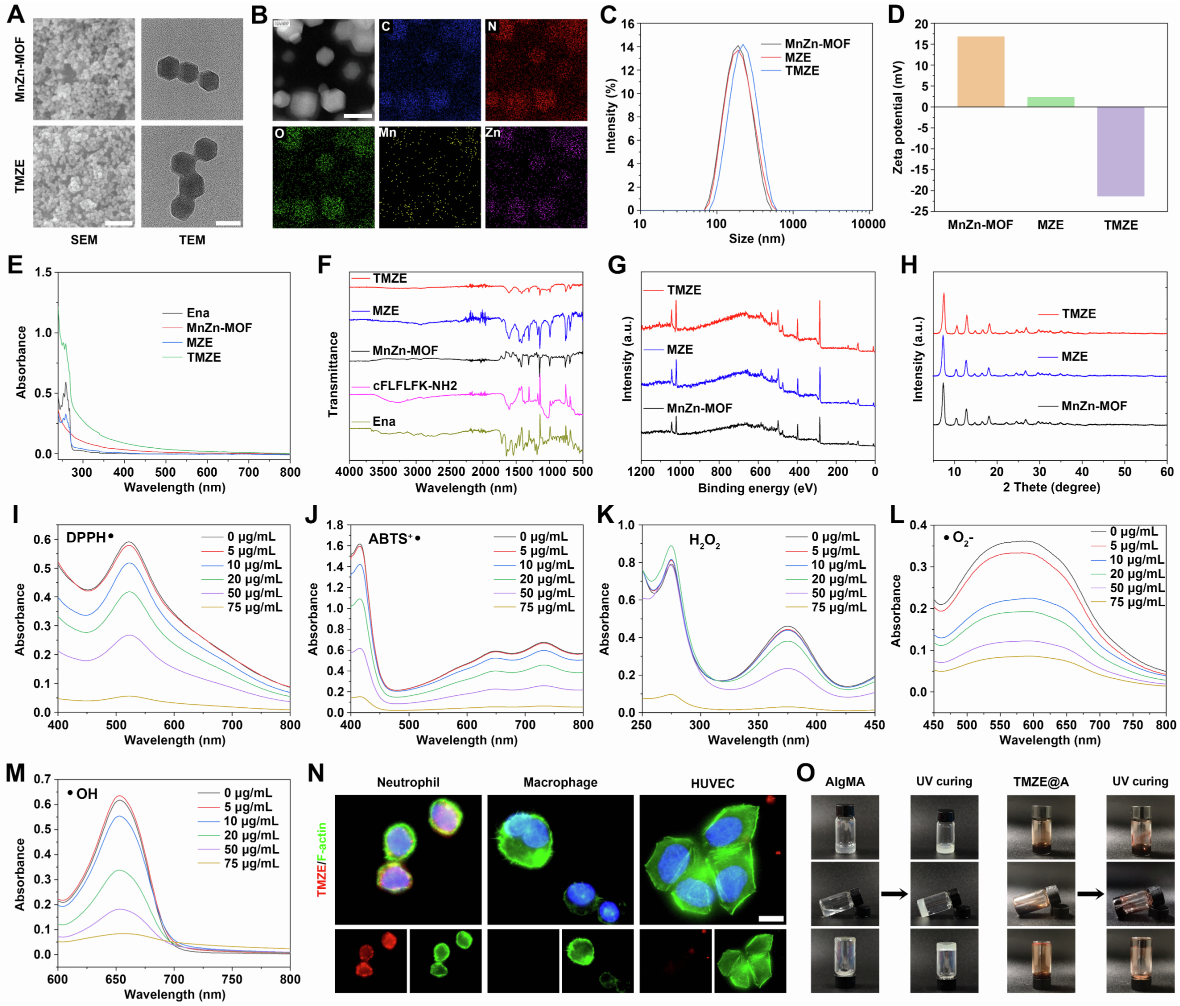

Figure S8. Manifestation of TMZE nanoparticles. Related to Figure 6.

(A) SEM and TEM were employed to disclose the appearance of MnZn-MOF and TMZE. Scale bar: 500 nm (SEM) and 50 nm (TEM); n = 3 independent experiments.

(B) Composite images were visualized by elemental mapping. Scale bar: 50 nm. n = 3 independent experiments.

(C and D) Particle size distribution and zeta potential of MnZn-MOF, MZE and TMZE. n = 3 independent experiments.

(E and F) UV-vis absorption spectra and Fourier transform infrared spectroscopy (FTIR) of each sample. n = 3 independent experiments.

(G and H) XPS spectra and powder XRD pattern of MnZn-MOF, MZE and TMZE. n = 3 independent experiments.

(L-M) Radical-eliminating activities of varied concentrations of TMZE for DPPH•, ABTS^{+}•, H_2O_2, •O_2^- and •OH as measured using UV-vis absorbance spectra. n = 3 independent experiments.

(N) Fluorescence staining was used to detect the uptake capacity of TMZE by multiple cells. Scale bar: 10 μm; n = 3 independent experiments.

(O) UV-curing property of AlgMA-based hydrogel. n = 3 independent experiments.

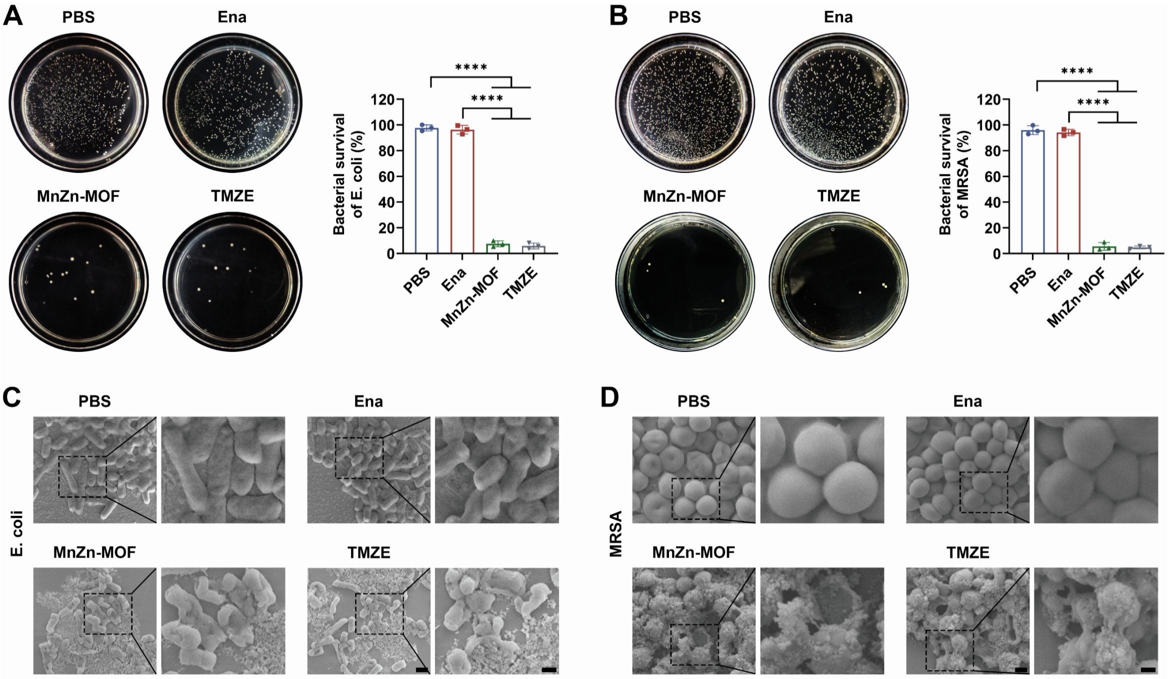

Figure S9. Antibacterial abilities of the drug carrier. Related to Figure 6.
(A and B) Bacteria-killing properties of MnZn-MOF and TMZE determined by the colony count method. n = 3 independent experiments.
(C and D) Morphology of E. coli and MRSA detected by SEM. Scale bar: 500 nm and 250 nm; n = 3 independent experiments.
Data were shown as mean ± standard deviation (S.D.) from biological replicates and statistical comparisons were performed using one-way ANOVA followed by Tukey’s multiple comparisons test in (A and B). ****$P < 0.0001$.

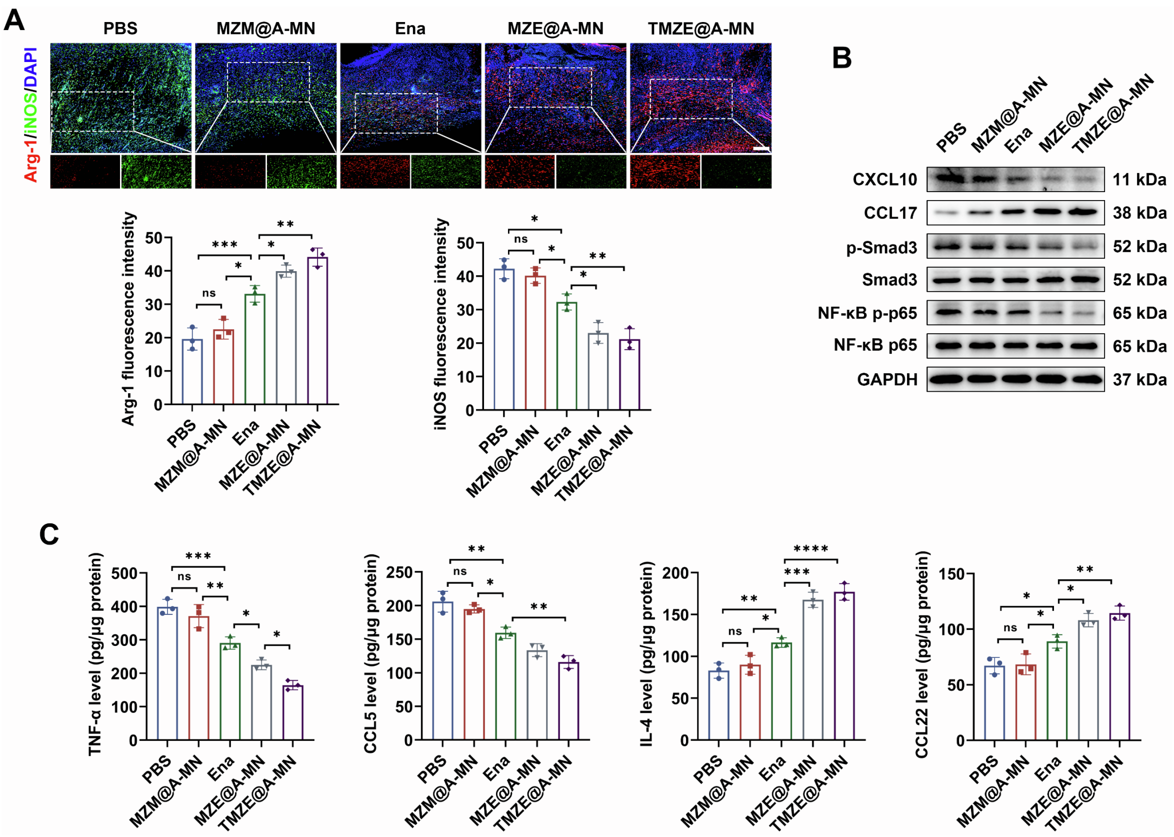

Figure S10. Effects of TMZE@A-MN on repressing inflammation responses of diabetic mouse wound tissues. Related to Figure 7.

(A) Levels of Arg-1 and iNOS in the wound area as measured using immunofluorescence staining. Scale bar: 200 μm; n = 3 biologically independent samples.

(B) Western blot detection of CXCL10, CCL17, Smad3 and NF-κB in wound tissues with different treatments. n = 3 biologically independent samples.

(C) Contents of TNF-α, CCL5, IL-4 and CCL22 in skin wounds as measured by ELISA kits. n = 3 biologically independent samples.

Data were shown as mean ± standard deviation (S.D.) from biological replicates and statistical comparisons were performed using one-way ANOVA followed by Tukey's multiple comparisons test in (A-C). ns: no significance, *$P < 0.05$, **$P < 0.01$, ***$P < 0.001$, ****$P < 0.0001$.

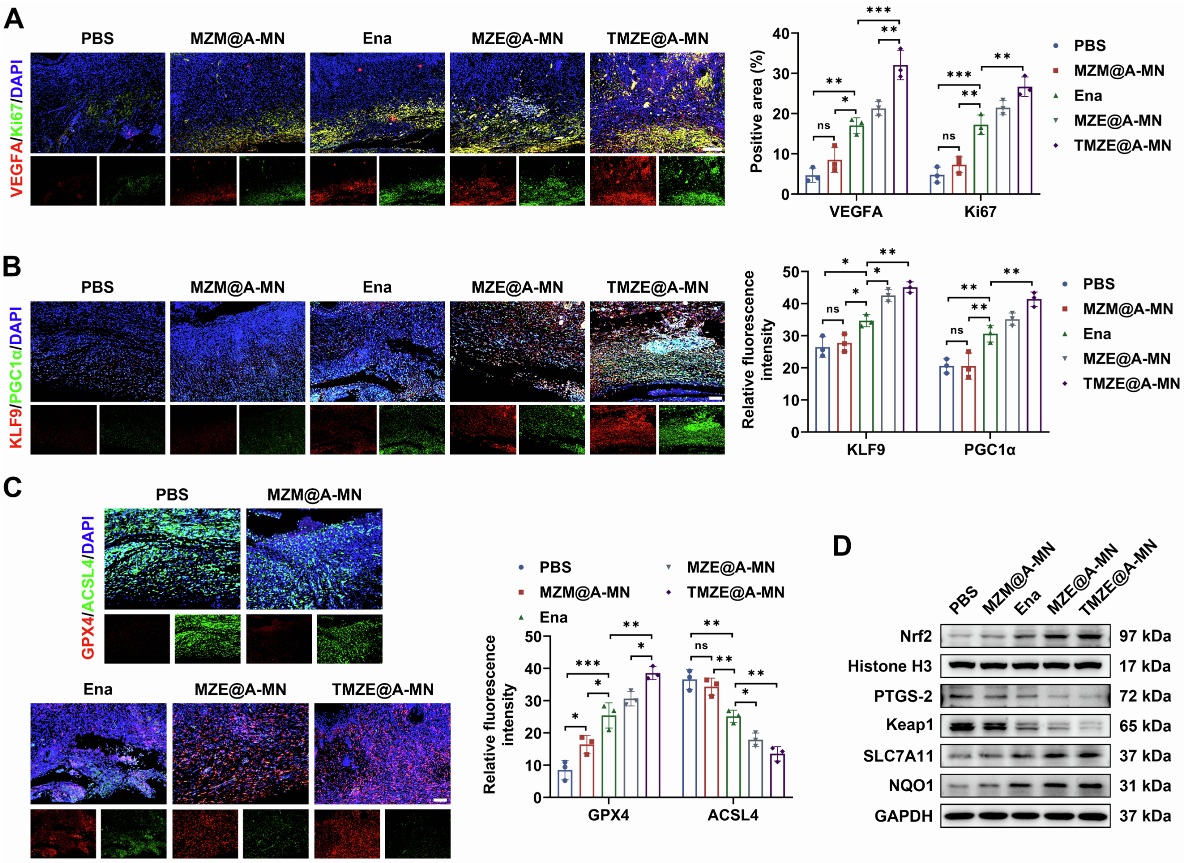

Figure S11. Regulatory roles of TMZE@A-MN in facilitating angiogenesis in the wound tissue of diabetic mice. Related to Figure 7.

(A) Expressions of VEGFA and Ki67 detected by immunofluorescence staining. Scale bar: 200 μm; n = 3 biologically independent samples.

(B) Fluorescence signals of KLF9 (red) and PGC1α (green) in the wound area. Scale bar: 100 μm; n = 3 biologically independent samples.

(C) Levels of GPX4 and ACSL4 measured by immunofluorescence staining. Scale bar: 100 μm; n = 3 biologically independent samples.

(D) Western blot analysis of Nrf2, PTGS-2, Keap1, SLC7A11 and NQO1 in the wound tissues with different treatments. n = 3 biologically independent samples.

Data were shown as mean ± standard deviation (S.D.) from biological replicates and statistical comparisons were performed using one-way ANOVA followed by Tukey's multiple comparisons test in (A-D). ns: no significance, *$P < 0.05$, **$P < 0.01$, ***$P < 0.001$.

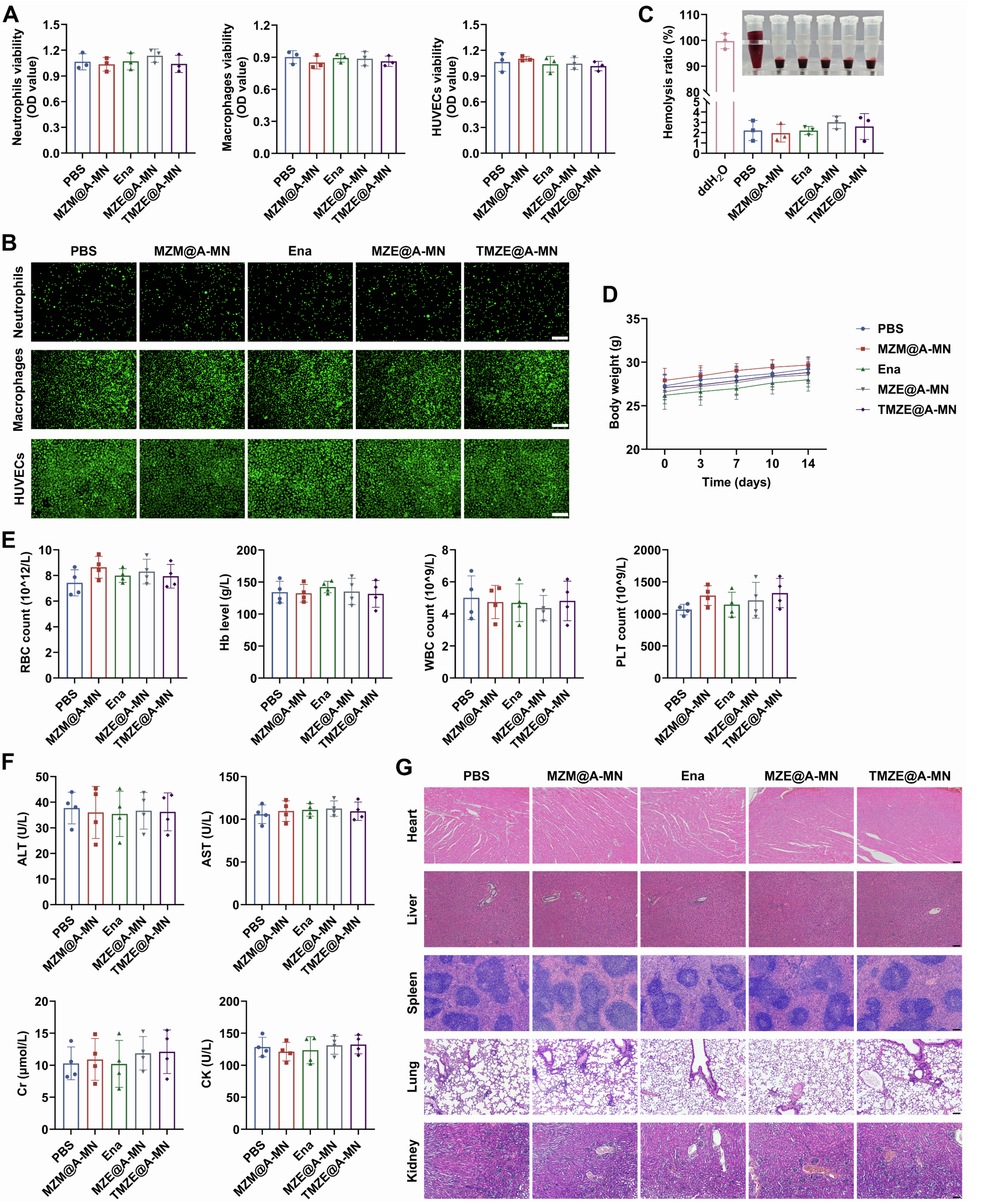

Figure S12. Biosafety of TMZE@A-MN tested in vitro and in vivo. Related to Figure 7.

(A and B) Viability of BMDNs, BMDMs and HUVECs as measured by CCK-8 assay and calcein/PI staining after incubation with different agents. Scale bar: 200 μm; n = 3 independent experiments.

(C) Results of hemolysis test. n = 3 independent experiments.

(D) Body weight changes of mice in each group. n = 4 mice per group.

(E) Main blood routine parameters as detected after mice were sacrificed. n = 4 biologically independent samples.

(F) Biochemical indicators in the blood of mice in each group. n = 4 biologically independent samples.

(G) H&E staining of visceral organs in mice with different treatment modalities for 14 days. Scale bar: 100 μm; n = 4 biologically independent samples.

Data were shown as mean ± standard deviation (S.D.) from biological replicates and statistical comparisons were performed using one-way ANOVA followed by Tukey's multiple comparisons test (A-E). The statistical differences showed above were not significant.

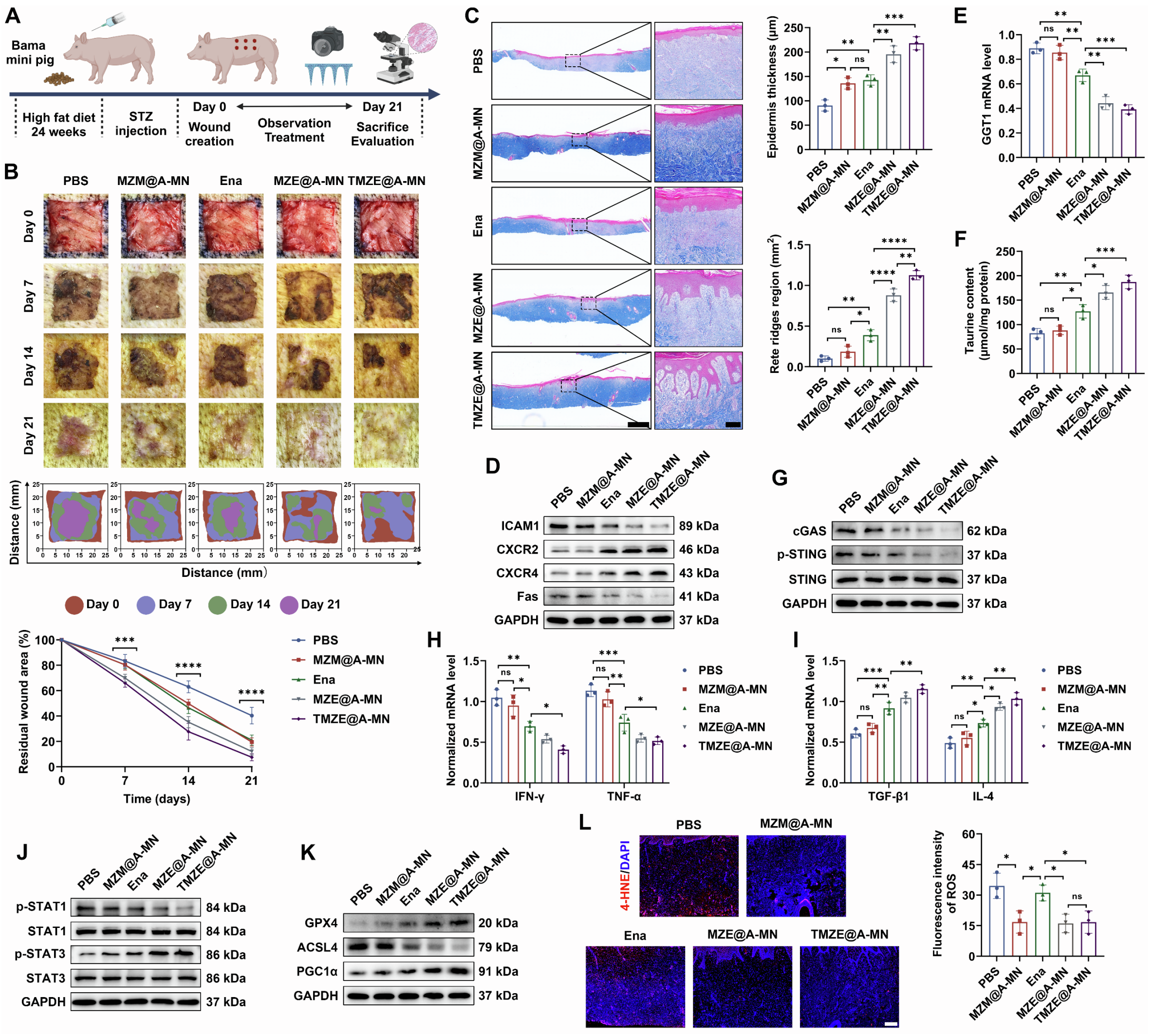

Figure S13. Beneficial roles of Ena-loaded delivery system in healing processes of porcine diabetic wounds. Related to Figure 7.

(A) Schematic illustration of the diabetic porcine wound establishment and the schedule of wound treatment *in vivo*.

(B) Representative wound images and wound closure rates of each group at days 0, 7, 14 and 21 post-surgery. n = 3 biologically independent samples.

(C) Masson's trichrome staining images of wound samples at day 21. Scale bar: 2.5 mm (left) and 250 μm (right); n = 3 biologically independent samples.

(D) Contents of ICAM1, CXCR2, CXCR4 and Fas in porcine wound tissues as determined by Western blot. n = 3 biologically independent samples.

(E and F) Quantitative analysis of GGT1 expression and taurine content within wound tissues after animals were sacrificed. n = 3 biologically independent samples.

(G-I) Activity of cGAS-STING pathway and expression of *ifnγ*, *tnfa*, *tgfb1* and *il4* in skin wounds. n = 3 biologically independent samples.

(J and K) Western blot was performed to measure the activation of STAT1 and STAT3 and the level of PGC1α, GPX4 and ACSL4 in diabetic porcine wounds. n = 3 biologically independent samples.

(L) ROS level in the wound tissue evaluated by immunofluorescence staining. Scale bar: 200 μm; n = 3 biologically independent samples.

Data were shown as mean ± standard deviation (S.D.) from biological replicates and statistical comparisons were performed using one-way ANOVA followed by Tukey's multiple comparisons test in (B-L). ns: not significant, $*P < 0.05$, $**P < 0.01$, $***P < 0.001$, $****P < 0.0001$.

Table S1. Demographic features of study participant. Related to STAR Methods.

Group	Patient	Age (years)	Gender	Diagnosis	Complication	Wound location	Sample size (cm^3)
DW	1	52	Male	Diabetes	Refractory ulcer	Foot	1 × 0.6 × 0.4
DW	2	59	Male	Diabetes	Refractory ulcer	Foot	0.8 × 0.8 × 0.5
DW	3	47	Male	Diabetes	Refractory ulcer	Foot	0.8 × 0.7 × 0.4
DW	4	46	Male	Diabetes	Refractory ulcer	Foot	1 × 0.8 × 0.6
DW	5	54	Male	Diabetes	Refractory ulcer	Foot	1 × 0.7 × 0.5
DW	6	63	Male	Diabetes	Refractory ulcer	Foot	0.9 × 0.6 × 0.5
AW	7	54	Male	Lower limb trauma	N/A	Foot	1 × 0.7 × 0.3
AW	8	50	Male	Lower limb trauma	N/A	Foot	0.9 × 0.6 × 0.4
AW	9	65	Male	Lower limb trauma	N/A	Foot	0.9 × 0.5 × 0.5
AW	10	49	Male	Lower limb trauma	N/A	Foot	1 × 0.7 × 0.5
AW	11	61	Male	Lower limb trauma	N/A	Foot	0.9 × 0.4 × 0.4
AW	12	51	Male	Lower limb trauma	N/A	Foot	0.7 × 0.6 × 0.5

Table S2. Sequences for siRNA design. Related to STAR Methods.

siRNA	Sense strand (5' to 3')	Antisense strand (5' to 3')
ZNF460	GGAUGGAGAUGAUAUUGUAGC	UACAAUAUCAUCUCCAUCCUA
GGT1	GCGGGAUCCUGUUCAAUAAUG	UUAUUGAACAGGAUCCCGCUG
KLF9	GGUUCAAAUAGGACUACUAGA	UAGUAGUCCUAUUUGAACCAU

Table S3. Primer sequences for ChIP. Related to STAR Methods.

Fragment	Forward (5' to 3')	Reverse (5' to 3')
RE1	TTTGAGACAGTCTCGCTCCG	AAATTAGTCAGGCGTGGTGG
RE2	TGAAGGAGGTAGGTGCTCTG	TGAAGGAGGTAGGTGCTCTG

Table S4. Primer sequences for qRT-PCR. Related to STAR Methods.

Gene	Forward (5' to 3')	Reverse (5' to 3')
**Mus musculus**		
IL-1β	TGCCACCTTTTGACAGTGATG	AAGGTCCACGGGAAAGACAC
CCL3	GTAGCCACATCGAGGGACTC	GATGGGGGTTGAGGAACGTG
Arg-1	AGCCAGGGACTGACTACCTT	TTGGGAGGAGAAGGCGTTTG
CCL17	AATGTAGGCCGAGAGTGCTG	TGCCCTGGACAGTCAGAAAC
IL-6	CCCCAATTTCCAATGCTCTCC	CGCACTAGGTTTGCCGAGTA
IL-12	GCCACCTACTCCCTTGGATCT	GACTGGCTAAGACACCTGGC
Retnla	GGGATGACTGCTACTGGGTG	TCAACGAGTAAGCACAGGCA
Chil3	TGTACCAGCTGGGAAGAAACAA	TGAGAGCAAGAAACAAGCATGG
**Homo sapiens**		
IL-1β	AGCTGACTGTCCTGGCTGAT	AGCTGACTGTCCTGGCTGAT
CCL3	ATTCCGTCACCTGCTCAGAA	GTCACACGCATGTTCCCAAG
Arg-1	GTCTGTGGGAAAAGCAAGCG	CACCAGGCTGATTCTTCCGT
CCL17	ATTCAAAACCAGGGTGTCTCC	CTCTTGTTGTTGGGGTCCGA
CDO	GGTGGGTCTCTTGCTGTCTC	TCAGCACTTCGGTCTGTTCC
CSAD	CTCAGAGCGGGTGAGCTTG	CACGTGTGGAGGCTAGTGTT
FMO1	TTGTTAGAGCAGCCAAGGGT	TTCGGTGAATCTCCACAGCC
BAAT	CAGGAGAGGGTCTCTTCCCA	AGAAAGTTGGCAGCCTCCTC
PTGS-2	TTGCATTCTTTGCCCAGCAC	ACCGTAGATGCTCAGGGACT
SLC7A11	TGGAACGAGGAGGTGGAGAA	TGGTGGACACAACAGGCTTT
NQO1	TGCTTACACTTACGCTGCCAT	CCAGTGGTGATGGAAAGCAC
GPX4	ATTGGTCGGCTGGACGAGG	TCGATGTCCTTGGCGGAAAA
**Sus scrofa**		
GGT1	GAAGGAGGACTGTCAGTGGC	CTCAATGCTGGGCTGGAAGA
IFN-γ	AGCTTTTCAGCTTTGCGTGA	TGCTCCTTTGAATGGCCTGG
TNF-α	CTGTAGGTTGCTCCCACCTG	ACACGCTTTTATTTCTCGCCA
TGF-β1	ACCTGCAAGACCATCGACAT	AGAGCAATACAGGTTCCGGC
IL-4	CTCCCAACTGATCCCAACCC	TGCACGAGTTCTTTCTCGCT
