# Supplementary material for: Enalaprilat reverses neutrophil polarization imbalance via targeting taurine-STING axis for treatment of diabetic wounds
Source: Cell Rep Med. 2026 Mar 30;7(4):102714. doi: 10.1016/j.xcrm.2026.102714 (PMC13130670; doi:10.1016/j.xcrm.2026.102714)
Supplement: Document S2. Article plus supplemental information [file mmc2.pdf]

Cell Reports
Medicine

Article

Enalaprilat reverses neutrophil polarization imbalance via targeting taurine-STING axis for treatment of diabetic wounds

Graphical abstract

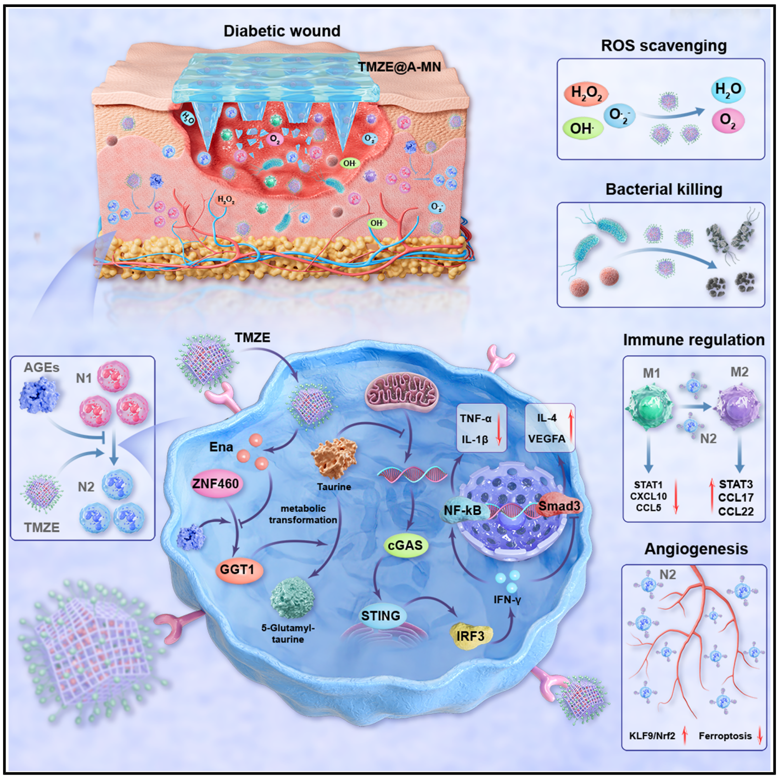

Highlights

- Neutrophils can experience polarization to inflammatory N1 phenotype
- Enalaprilat induces N2 conversion via inhibiting STING axis
- Enalaprilat-affected neutrophils facilitate formation of regeneration-friendly niche
- Microneedle-based delivery system is beneficial for the diabetic wound treatment

Authors

Li Lu, Yuan Xiong, Jiewen Liao, ..., Guohui Liu, Yanli Zhao, Bobin Mi

Correspondence

liuguohui@hust.edu.cn (G.L.), zhaoyanli@ntu.edu.sg (Y.Z.), mibobin@hust.edu.cn (B.M.)

In brief

Lu et al. develop a multifunctional microneedle nanosystem for transdermal delivery of enalaprilat. Enalaprilat promotes neutrophil repolarization from pro-inflammatory N1 to anti-inflammatory N2 state via taurine metabolic reprogramming, efficiently improving macrophage inflammation and angiogenic processes for accelerating diabetic wound healing.

Lu et al., 2026, Cell Reports Medicine 7, 102714
April 21, 2026 © 2026 The Author(s). Published by Elsevier Inc.
https://doi.org/10.1016/j.xcrm.2026.102714

CellPress

Cell Reports Medicine

CellPress
OPEN ACCESS

Article

Enalaprilat reverses neutrophil polarization imbalance via targeting taurine-STING axis for treatment of diabetic wounds

Li Lu,[1,2,8] Yuan Xiong,[3,8] Jiewen Liao,[1,8] Juan Zhou,[4] Guangji Wang,[5] Yating Qin,[6] Shengming Zhang,[1] Yanzhi Zhao,[1] Xiaodan Zhong,[6] Mengwen Wang,[6] Kangkang Zha,[1] Fawwaz Al-Smadi,[1] Guohui Liu,[1,*] Yanli Zhao,[7,*] and Bobin Mi[1,7,9,*]
[1]Department of Orthopedics, Union Hospital, Tongji Medical College, Huazhong University of Science and Technology, Wuhan 430022, China
[2]Department of Rehabilitation, Tongji Hospital, Tongji Medical College, Huazhong University of Science and Technology, Wuhan 430030, China
[3]Department of Orthopedic Surgery, Tongji Hospital, Tongji Medical College, Huazhong University of Science and Technology, Wuhan 430030, China
[4]Department of Cardiology, Hubei Provincial Hospital of Traditional Chinese Medicine, Wuhan 430073, China
[5]Department of Cardiology, The Central Hospital of Wuhan, Tongji Medical College, Huazhong University of Science and Technology, Wuhan 430073, China
[6]Department of Cardiology, Tongji Hospital, Tongji Medical College, Huazhong University of Science and Technology, Wuhan 430030, China
[7]School of Chemistry, Chemical Engineering and Biotechnology, Nanyang Technological University, 21 Nanyang Link, Singapore 637371, Singapore
[8]These authors contributed equally
[9]Lead contact
*Correspondence: liuguohui@hust.edu.cn (G.L.), zhaoyanli@ntu.edu.sg (Y.Z.), mibobin@hust.edu.cn (B.M.)
https://doi.org/10.1016/j.xcrm.2026.102714

SUMMARY

Persistent inflammation derived from neutrophil activation drives delayed healing of diabetic wounds. Herein, a dissolvable alginate methacryloyl-based microneedle patch functionalized with polypeptide CFLFLFK-NH_2-coupled manganese/zinc ion metal-organic framework (MnZn-MOF) loading enalaprilat (Ena) (TMZE@A-MN) is developed. Ena promotes neutrophil repolarization from pro-inflammatory N1 to anti-inflammatory N2 state by inhibiting nuclear factor (NF)-κB axis and activating Smad3 pathway, attributed to Ena-induced level elevation of taurine and subsequently STING signaling cascade suppression, thus causing macrophage phenotype switching and endothelial cell ferroptosis repression. Due to identifiable property of CFLFLFK-NH2 on neutrophil membrane receptors, the delivery system endows Ena with targeting inhibitory roles in neutrophil activation. In addition, MnZn-MOFs possess free radical-eliminating performance and can effectively combat the growth of methicillin-resistant *Staphylococcus aureus* and *Escherichia coli*. *In vivo* evaluation on diabetic murine and porcine wounds also demonstrates that the TMZE@A-MN accelerates wound healing process. Consequently, the targeted microneedle delivery system holds great promise for diabetic wound treatment.

INTRODUCTION

Diabetic foot ulcers (DFUs), characterized by prolonged healing time, high recurrence rate, and high risks of amputation and mortality, represent a profoundly widespread complication in the diabetic cohort.[1,2] Mounting evidence has established that neutrophils display heterogeneous phenotypes and high plasticity in different pathological contexts.[3–5] N1 neutrophils exert inflammatory effects to eradicate extraneous bacteria and abnormal tissues, whereas N2 neutrophils exhibit immune-suppressive behaviors to promote tumor metastasis and angiogenesis, improve myocardial and neural protection, and favor bone regeneration.[6–8] As the first arrival and most abundant type of leukocytes in the wound region, neutrophils might provide contributing roles in halting inflammation phase transition during diabetic tissue repair through reprograming failure from N1 to N2 phenotype.[9,10]

Apart from disrupting the conversion of angiotensin, enalapril is suggested to repress inflammation responses to alleviate the progression of multiple diseases, especially myocardial and cerebral ischemia.[11,12] It is demonstrated that inflammation stress-induced vascular structure disarrangement and regeneration dysfunction exert pivotal roles in aggravating shortage of blood supply, among which pro-inflammatory neutrophils and associated damaged endothelial cells are involved.[13,14] Notably, the association of upregulated angiotensin-converting enzyme (ACE) with activated neutrophil immune response has been revealed.[15] Moreover, enalapril is reported to alleviate the progression of diabetic nephropathy via mitigating microvascular injury, which is also an important predisposing factor prone to delayed

This is an open access article under the CC BY license (http://creativecommons.org/licenses/by/4.0/).

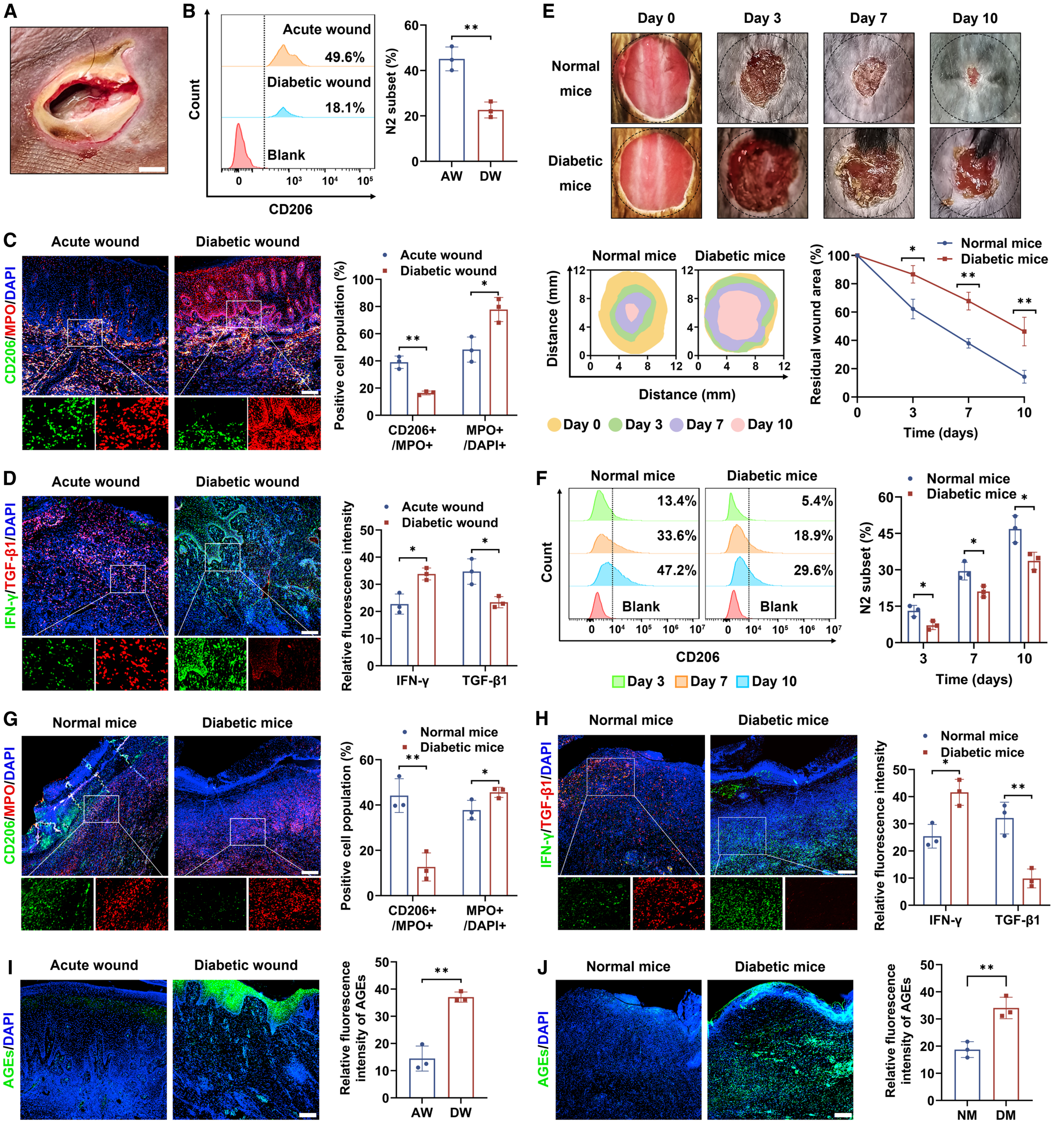

Figure 1. Neutrophil polarization and inflammation profile in skin wound tissues

(A) Representative appearance of diabetic wounds. Scale bar, 10 mm.

(B) Populations of CD206+ neutrophils in the AW and DW tissues as determined by flow cytometry. AW, acute wound; DW, diabetic wound; $n = 3$ biologically independent samples.

(C) Immunofluorescence staining to measure the distribution of CD206 and MPO in skin wound area. Scale bar, 200 μm; $n = 3$ biologically independent samples.

(D) Expression of IFN-γ and TGF-β1 evaluated by immunofluorescence analysis. Scale bar, 200 μm; $n = 3$ biologically independent samples.

(E) Wound closure rate of normal and diabetic mice on days 0, 3, 7, and 10 post-operation. $n = 3$ mice per group.

(F) Flow cytometry was employed to detect the change tendency of CD206+ neutrophil percentage in the wound region following the healing time extension. $n = 3$ biologically independent samples.

(G) Positive area of CD206 and MPO in wound tissues on day 7 post-surgery from normal mice and diabetic mice as assessed using immunofluorescence staining. Scale bar, 200 μm; $n = 3$ biologically independent samples.

(legend continued on next page)

healing of DFUs.[16] Thus, enalapril possibly suppresses N1 neutrophil-related immune activation to facilitate angiogenic processes, contributing to tissue repair in diabetic wounds. However, as the incidence of cough and angioedema is not uncommon when exposed to the systemic use of enalapril, it is worthy to develop safe and feasible a local drug delivery system to conquer the side effects and heighten the anti-inflammatory efficiency of enalapril.

Metal-organic frameworks (MOFs) are a group of porous nanoplatforms built from metal ions or cluster nodes bridged by organic linkers and possess good biodegradability, responsive drug-controlled release, and high loading capacities, determining them to be promising systems in medical application for drug delivery.[17–19] Since MOFs have been developed to possess therapeutic functions to achieve synergetic effects with the encapsulated drugs for wound management, we introduce an antibacterial and antioxidative manganese/zinc ion MOF (MnZn-MOF)-enveloped microneedle (MN) patch as a drug vehicle. Enalaprilat (Ena), the active pharmacological component of enalapril used *in vivo*, is loaded into the MnZn-MOF for overcoming the drawbacks of rapid release, low tissue retention, and activity loss. The obtained system is named MZE. After combining with neutrophil-targeted peptide (named TMZE), the nanosystem is immersed in alginate methacryloyl (AlgMA) hydrogel-based MN (TMZE@A-MN), which guarantees the transdermal delivery of Ena and sufficient remedy range. *In vivo* evaluation further verifies that the multifunctional TMZE@A-MN exerts promotive effects on diabetic wound repair, potentiating its potential application for clinical treatment of DFU.

RESULTS

Co-existence of reduced N2 neutrophil cohort and immune niche hostile to regeneration in the diabetic wound

The flow cytometry of skin tissues from diabetic and non-diabetic patients showed that the percentage of N2 neutrophils in acute wounds increased to a level more than double that in diabetic wounds (Figures 1A and 1B). This result was further corroborated by the immunofluorescent detection against N2 marker CD206. In contrast, the positive fluorescence signal of myeloperoxidase (MPO), a constitutively expressed landmark of neutrophils, was increased by approximately 1.5 times in the tissue of diabetic group compared to that in the acute group, suggesting the impaired neutrophil inflammation resolution in the diabetic niche (Figure 1C). The extent of pro-inflammatory interferon (IFN)-γ of diabetic wounds outperformed that of acute wounds, yet anti-inflammatory transforming growth factor (TGF)-β1 yielded opposite trend, further showing the disordered immune microenvironment unfriendly to angiogenesis and regeneration in diabetic wound area, as confirmed by the lower newly formed vessel density and decreased proportion of proliferation marker Ki67 (Figures 1D and S1A).

Similar neutrophil polarization was also witnessed in murine wound tissues. In the same time point, diabetic mice with larger residual wound area developed reduced N2 population in the wounds as compared to the normal mice. Meanwhile, with the development of wound healing, the percentage of N2 within neutrophils in skin tissues was on the rise, suggesting the implication of N2 phenotype in facilitating wound healing (Figures 1E and 1F). At day 7 post-operation, compared to the normal mice, wounds of diabetic mice possessed higher MPO-positive cells, among which CD206+ population was reduced (Figure 1G). Immunofluorescent staining revealed enhanced inflammation, impaired neovascularization, and repressed regeneration activity in the diabetic murine wound as well (Figures 1H and S1B).

Advanced glycation end products (AGEs) are involved in the structural and functional alterations of multiple organs in diabetic individuals like retinopathy, keratopathy, and vasculopathy.[20–22] In terms of the skin wound, higher level of AGEs was seen in the wound area of diabetic patients in comparison with that of acute wounds (Figure 1I). Such an observation was in line with wound AGE measurement of murine models (Figure 1J). Given that AGEs activated inflammation signals to cause tissue damage, abundance of AGEs probably disrupted neutrophil reprogramming from N1 to N2 state, thereby delaying the skin tissue rehabilitation of DFUs.

Ena treatment alleviated angiogenesis dysfunction and inflammation expansion via antagonizing AGE-induced N1 polarization of neutrophils

Ena has been reported to suppress inflammation cascades, whereas the association of Ena with the polarization conversion of neutrophils is ambiguous.[11] As shown in Figure 2A, the AGE stimulation reduced the percentage of CD206+ bone marrow-derived neutrophils (BMDNs), which was reversed upon Ena treatment. Further characterization of N1 biomarker ICAM1 and Fas and N2 indicator CXCR2 and CXCR4 manifested repressed polarization skewing toward N1 stage in response to Ena exposure (Figures 2B and 2C). Corroboratively, the AGE-evoked elevation of pro-inflammatory *il1b* and *ccl3* was abated by Ena and diminution of anti-inflammatory *arg1* and *ccl17* caused by AGEs was normalized under the treatment of Ena, separately (Figure 2D).

Subsequently, the pro-angiogenic functions of human umbilical vein endothelial cells (HUVECs) affected by preconditioned neutrophils were evaluated (Figure 2E). BMDNs pretreated with AGEs induced a reduced 5-ethynyl-2′-deoxyuridine (EdU)-positive ratio of HUVECs, which was increased by addition of Ena (Figure 2F). Transwell assay and cell scratch test both revealed

(H) Expression of IFN-γ and TGF-β1 in wound tissues on day 7 post-surgery from normal mice and diabetic mice as assessed using immunofluorescence staining. Scale bar, 200 μm; $n = 3$ biologically independent samples.

(I) Wound tissues from diabetic patients exhibited higher level of AGEs than those from non-diabetic samples. Scale bar, 200 μm; $n = 3$ biologically independent samples.

(J) Wound tissues from mice exhibited higher level of AGEs than those from non-diabetic samples. Scale bar, 200 μm; $n = 3$ biologically independent samples. Data were shown as mean ± standard deviation (SD) from biological replicates, and statistical comparisons were performed using unpaired Student's t test in (B–D and G–J) and one-way ANOVA followed by Tukey's multiple comparisons test in (E and F). $^{*}p < 0.05$, $^{**}p < 0.01$.

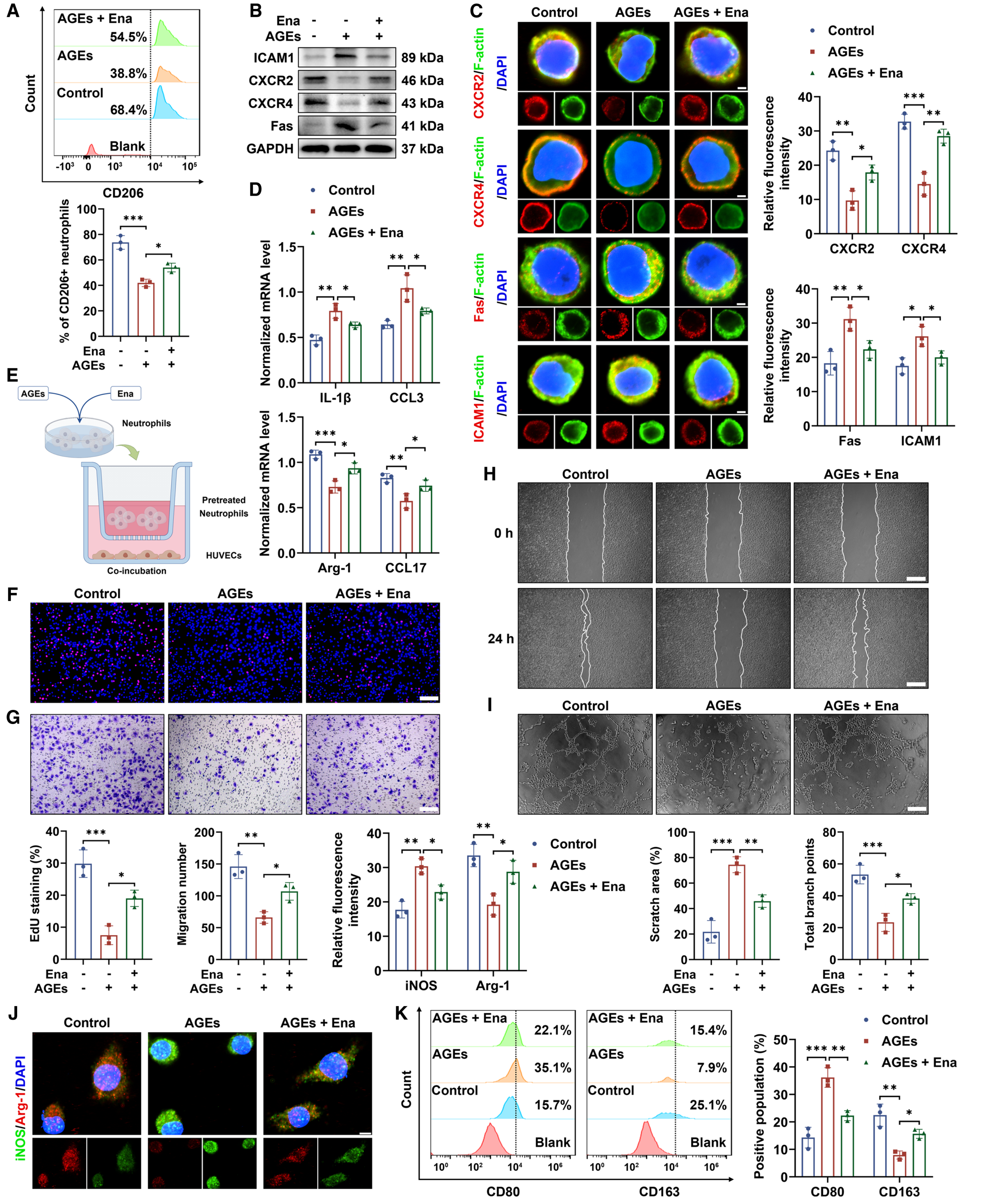

Figure 2. Phenotype repolarization of neutrophils induced by Ena affected the pro-angiogenic capacities of HUVECs and inflammatory state of macrophages

(A) Flow cytometry was adopted to reveal the difference of CD206+ population ratio among the three groups. *n* = 3 independent experiments.

(B) Effects of AGE stimulation and sequential Ena supplement on the expression of ICAM1, CXCR2, CXCR4, and Fas as evaluated by western blot.

(legend continued on next page)

that there was a declining number of migrated cells exposed to the pro-inflammatory N1 cohort compared to those co-cultured with resting neutrophils. Instead, more cells were observed to move within the milieu affected by Ena-prompted N2 population (Figures 2G and 2H). Then, compared to the quiescent circumstance, AGE priming yielded fewer capillary branches and short length of total tubes. On the contrary, Ena supplement developed better tube formation performance with more tube branch points and vessel-like structures (Figure 2I).

Considering pivotal roles of macrophages in orchestrating inflammation and tissue regeneration, murine bone marrow-derived macrophages (BMDMs) affected by neutrophils were applied for ascertaining the inflammation profiles.[23] We found that the green fluorescence of M1 marker iNOS was enhanced and the red fluorescence of M2 indicator Arg-1 was weakened in macrophages irritated by AGE-pretreated BMDNs, which was ameliorated when exposed to these neutrophils with Ena preconditioning (Figure 2J). Consistently, the repressed roles of Ena in the elevation of $CD80^{+}$ M1 proportion and reduction of CD163+ M2 percentage induced by AGEs were confirmed using the flow cytometry as well (Figure 2K).

Multi-omics analysis disclosed taurine accumulation in Ena-treated neutrophils

To delve deeper into underlying mechanisms by which Ena promoted neutrophil phenotype repolarization, multi-omics analysis was performed (Figure 3A). RNA sequencing revealed differentially expressed genes (DEGs) between the two groups, with 434 encountering up-regulation and 405 experiencing down-regulation (Figures 3B and S2A). Then, Gene Ontology (GO) enrichment showed that DEGs were primarily enriched in the regulation of immune responses (Figure S2B). Inflammation-related pathways such as nuclear factor (NF)-κB were identified to be enriched using Kyoto Encyclopedia of Genes and Genomes (KEGG) database and gene set enrichment analysis (GSEA) (Figures 3C and S2C). Moreover, repression of glycolytic gene sets and enhancement of gene sets linked with amino acid metabolism were observed (Figure S2D).

Then, principal-component analysis of metabolomics demonstrated the differences in the metabolite profiles of BMDNs between the two groups with good sample reproducibility (Figure S3A). There were 169 differentially accumulated metabolites (DAMs) with reduced content and 223 DAMs with increased level screened in AGE-stimulated neutrophils exposed to Ena, as compared to that without Ena intervention (Figures S3B and S3C). We found that the DAMs were divided into 13 subclasses, mainly containing organic acids and derivatives (30.77%), lipids and lipid-like molecules (18.05%), and organoheterocyclic compounds (11.83%) (Figure 3D). Small Molecule Pathway Database analysis showed obvious pathways enriched by DAMs, including methionine, glutamate, and purine metabolism cascades (Figure S3D).

To gain an in-depth insight into the relationship of obtained DAMs with DEGs, integrated analysis of multi-omics was performed, which showed 97 shared pathways of KEGG enrichment (Figures 3E and 3F). Among them, glutathione metabolism was revealed to affect immune functions of neutrophils via maintaining redox homeostasis to improve chemotaxis, degranulation, and formation of neutrophil extracellular traps.[24] Moreover, pyrimidine metabolism was not only associated with neutrophil phagocytosis but also regulated inflammasome-dependent innate immunity in neutrophils.[25] Enriched DAMs comprised up-regulated taurine, proline, and glucose 1-phosphate and down-regulated spermidine, arginine and pyridoxal phosphate (Figure 3G). Then, above metabolites like taurine and its derivatives were positively associated with genes responsible for immune regulation, including *TNFSF10*, *CCL7*, *CCL2*, and *IL18* (Figure 3H). Given that taurine exhibited key roles in repressing inflammation and improving cellular viability, we hypothesized that Ena impeded the inflammatory phenotype of neutrophils relying on level increase of taurine.[26,27]

Ena regulated taurine-dominated metabolic reprogramming relying on ZNF460-GGT1 axis

Taurine, a type of conditionally essential amino acid, is widely distributed in multiple tissues for maintaining metabolic homeostasis.[28] Herein, an enhanced content of taurine was found in AGE-induced neutrophils following Ena treatment, and *γ-glutamyltransferase 1* (GGT1) required for the bioconversion of taurine displayed a decreased expression, providing the possibility that Ena-triggered taurine level elevation was attributed to expression suppression of *GGT1*, which in turn showed restriction on taurine transformation (Figures 4A and 4B).

Using the JASPAR and UCSC database, we found that zinc finger protein 460 (ZNF460) was predicted as the transcriptional factor of *GGT1* with the highest binding score and there were two putative response elements (REs) for ZNF460 within the *GGT1* promoter region, named RE1 (from −917 to −902) and

(C) Effects of AGE stimulation and sequential Ena supplement on the expression of ICAM1, CXCR2, CXCR4, and Fas as evaluated by immunofluorescence staining. Scale bar, 2 μm; $n = 3$ independent experiments.
(D) Quantitative reverse-transcription PCR (RT-qPCR) was employed to quantify the level of *il1b*, *ccl3*, *arg1*, and *ccl17*. $n = 3$ independent experiments.
(E) Schematic diagram for the incubation of HUVECs with pretreated neutrophils.
(F) Proliferative ability of HUVECs under the stimulation of neutrophils with indicated preconditioning as detected via EdU staining. Scale bar, 200 μm; $n = 3$ independent experiments.
(G) Migration property of HUVECs as measured by transwell assay. Scale bar, 200 μm; $n = 3$ independent experiments.
(H) Migration property of HUVECs as measured by wound scratch test. Scale bar, 500 μm.
(I) Tube formation assay was performed to visualize the neovascularization function of HUVECs with different treatments. Scale bar, 200 μm; $n = 3$ independent experiments.
(J) Phenotype characters of macrophages irritated by neutrophils as determined by immunofluorescence staining. Scale bar, 5 μm.
(K) Phenotype characters of macrophages irritated by neutrophils as determined by flow cytometry. $n = 3$ independent experiments.
Data were shown as mean ± standard deviation (SD) from biological replicates, and statistical analyses were performed using one-way ANOVA test followed by Tukey's multiple comparisons test in (A–D and F–K). *$p < 0.05$, **$p < 0.01$, ***$p < 0.001$.

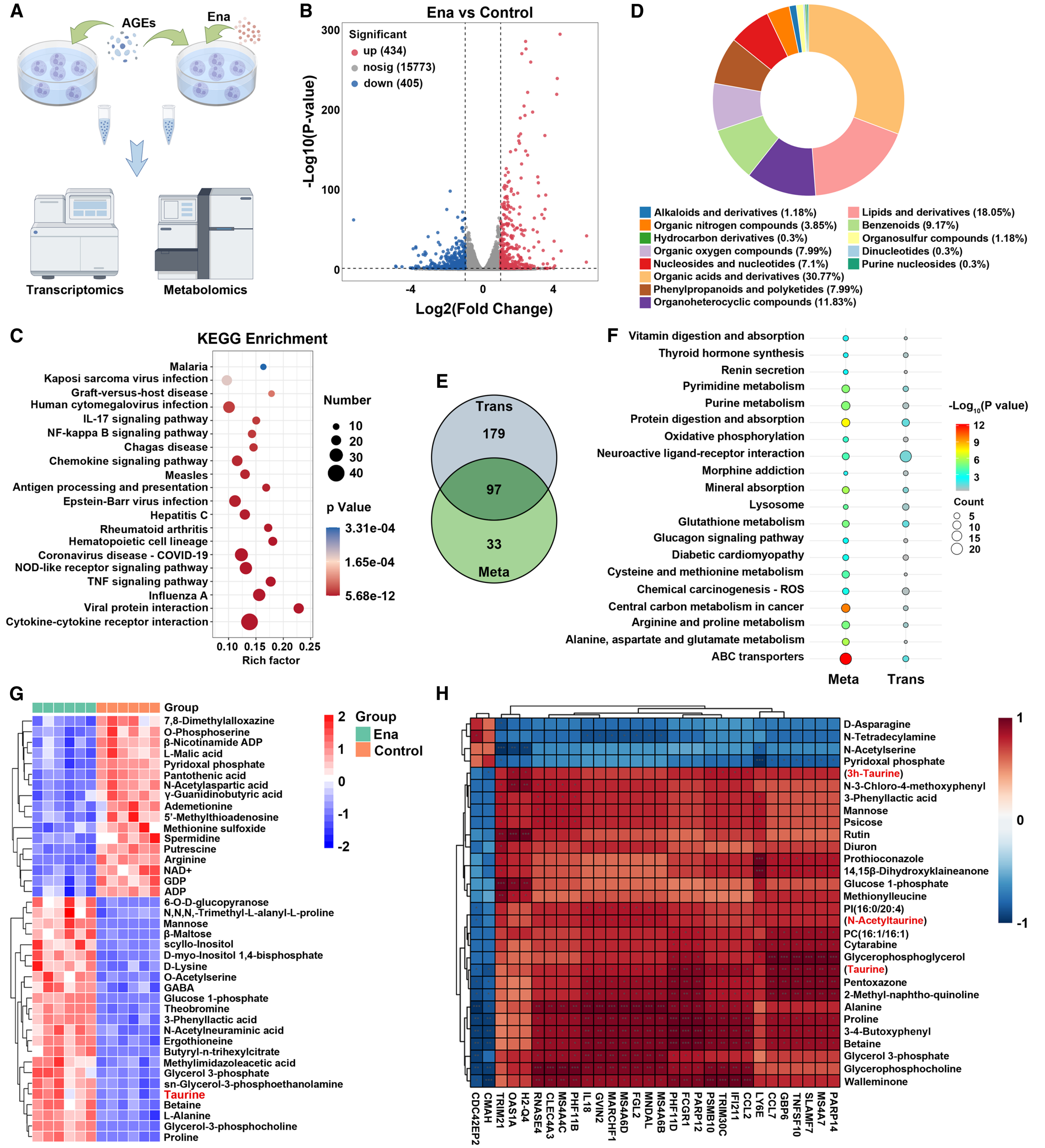

Figure 3. Multi-omics detection for revealing the gene expression and metabolic profiles stimulated by Ena

(A) Illustration of the procedures for the performance of transcriptomics and metabolomics.

(B) Volcano plot to present the DEG profiles regulated by Ena.

(C) KEGG analysis of the top 20 enriched pathways.

(D) Main metabolite categories among the DAMs.

(E) Shared significant pathways enriched by DEGs and DAMs.

(F) Top 20 shared KEGG pathways.

(G) Heatmap of the top 40 DAMs associated with the shared KEGG pathways.

(H) Correlation analysis of DEGs and DAMs.

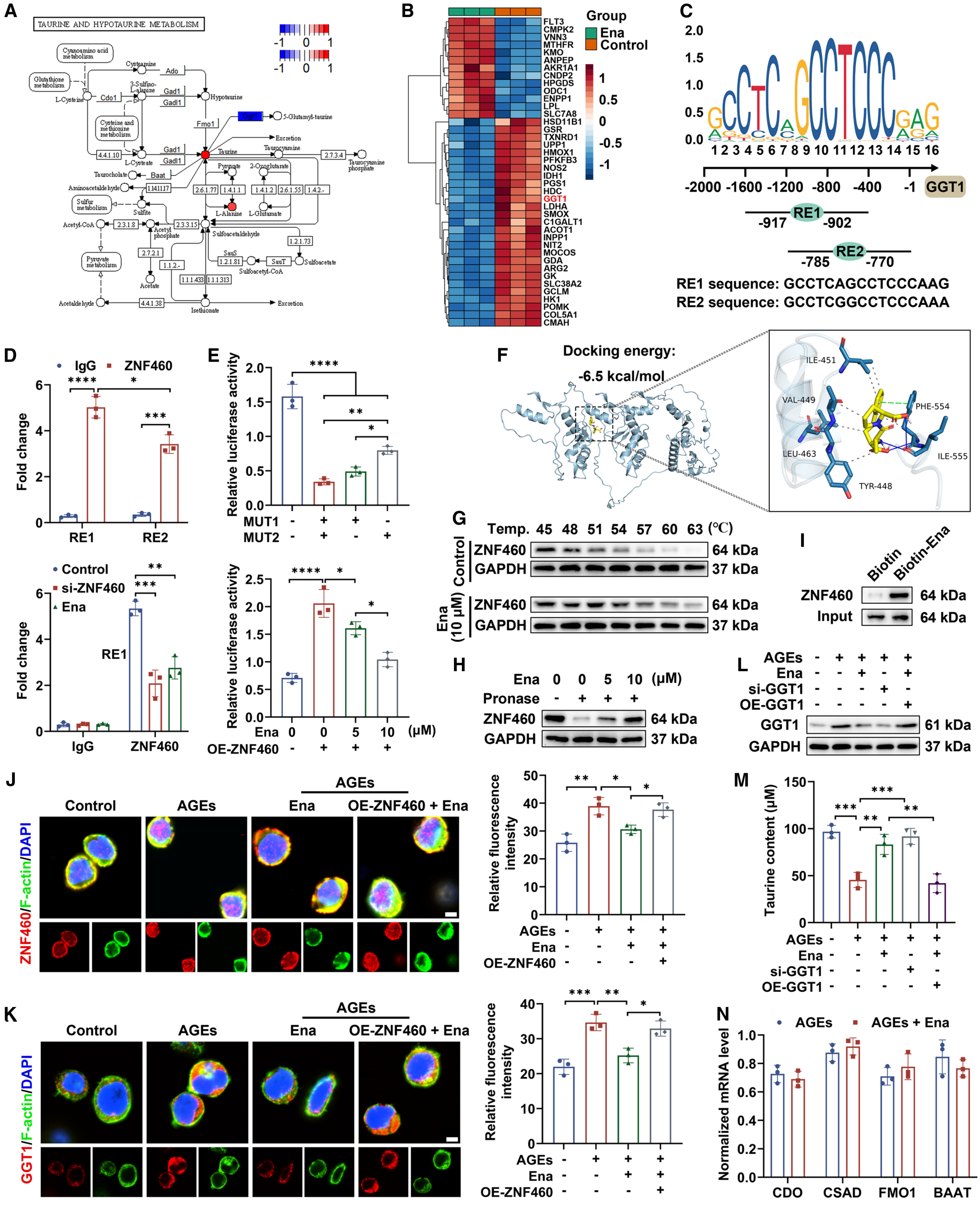

Figure 4. Regulatory roles of Ena in ZNF460-GGT1 axis-mediated taurine metabolism
(A) KEGG pathway analysis of taurine metabolism.
(B) Heatmap of top 40 significant DEGs.
(C) Predicted binding sites of ZNF460 in the promoter of *GGT1*.

(legend continued on next page)

RE2 (from −785 to −770) (Figure 4C). Then, we discovered that the RE1 and RE2 sequences in the *GGT1* promoter were enriched in ZNF460-ChIPed DNA fragments but not in IgG-ChIPed negative controls. Moreover, the chromatin imunoprecipitation (ChIP) enrichment efficiency for ZNF460 in the RE1 region was dramatically repressed by the intervention of both the si-ZNF460 and Ena (Figure 4D). Then, the wild-type (WT) promoter group possessed enhanced luciferase activity than the mutant promoter group, among which incubation of RE2 mutant plasmids (MUT2) induced an elevated fluorescence intensity than RE1 mutant plasmids (MUT1). Additionally, overexpression of ZNF460 markedly increased the WT *GGT1* promoter reporter activity, which was reversed by Ena in a dose-dependent manner (Figure 4E).

To evaluate the binding affinity of Ena for ZNF460, molecular docking was carried out, showing that Ena combined with the amino acid residue Phe 554 and Ile 555 via hydrogen bonds and developed hydrophobic interaction with Tyr 448, Val 449, Ile 451, and Leu 463 of ZNF 460, presenting a mean binding energy of −6.5 kcal/mol (Figure 4F). Then, cellular thermal shift assay showed that there was an increased tendency of ZNF460 protein denaturation with rising temperature ranging from 45°C to 63°C, which was alleviated by Ena (Figure 4G). The binding potential was further confirmed by drug affinity responsive target stability test and pull-down assay (Figures 4H and 4I). Notably, Ena impeded nuclear recruitment of ZNF460 and expression of GGT1 triggered by AGEs, which vanished when cells were pretreated with OE-ZNF460 plasmid, implying that interaction with Ena retained ZNF460 in the cytoplasm and then led to the expression reduction of target gene GGT1 (Figures 4J and 4K). Moreover, the level of GGT1 and concentration of taurine demonstrated the opposite trend in AGE-irritated cells exposed to indicated treatments (Figures 4L and 4M). Other key enzymes responsible for taurine metabolism including *cdo*, *csad*, *fmo1*, and *baat* kept unaltered, further verifying the dominant roles of Ena-induced GGT1 content decrement in alleviating the catabolism of taurine (Figure 4N).

Ena regulated neutrophil repolarization and pro-regenerative niche formation relying on taurine-induced cGAS-STING axis inhibition

Considering the crucial roles of double-stranded DNA (dsDNA)/cGAS/STING axis in inflammation activation, elevation of dsDNA content and cGAS level was seen in AGE-elicited BMDNs, which was normalized via Ena treatment (Figure S4A).[29–31] Then, AGE-triggered phosphorylation increase of STING, TBK1, and IRF3 in neutrophils was suppressed by Ena supplement and reappeared following OE-*GGT1* pretreatment (Figure S4B). Taurine addition neutralized the effects of *GGT1* overexpression on STING cascade activation and downstream IFN-γ production (Figure S4C). Afterward, repressed contents of *il1b* and *ccl3* were seen by the treatment of Ena or STING antagonist H-151 in the AGE-priming condition, in dramatic contrast to usage of STING agonist diABZI. An entirely different variation trend was manifested in levels of *arg1* and *ccl17* (Figure S4D). In parallel with H-151 replenishment, Ena produced decreased level of tumor necrosis factor alpha as well as enhanced contents of interleukin (IL)-4 and VEGFA, which was counteracted by diABZI (Figure S4E). NF-κB involved in N1 conversion had a comparable activity between the Ena and H-151 treatment, remarkably lower than AGE and diABZI stimulation.[32] Instead, the activation of Smad3 responsible for N2 switching experienced a distinct tendency (Figure S4F).[33] These findings disclosed that neutrophil phenotype repolarization observed for Ena was endowed by level elevation of taurine, which restrained cGAS-STING signaling, accompanied by NF-κB axis inhibition and Smad3 cascade enhancement.

As the first responder to tissue damage, neutrophils might induce adjacent immune cells to activate and release excessive pro-inflammatory cytokines detrimental to wound repair.[34,35] We found that BMDMs experienced phenotype transformation from M1 to M2 when exposed to AGE-induced neutrophils pretreated with Ena. This effect was alleviated by *GGT1* overexpression of neutrophils, and then taurine supplement in neutrophils triggered content decrease of CD80-positive population and level reduction of *il6*, *il12*, and IL-1β. Reversely, the percentage of CD163-positive BMDMs and content of intracellular *retnla*, *chil3*, and TGF-β1 displayed the opposite trend (Figures S5A–S5D). The underlying mechanisms were possibly attributed to reduced activity of STAT1 involved in M1 conversion and elevated activity of STAT3 associated with M2 switching (Figure S5E).[36] Decreased expression of CXCL10 and CCL5 and increased level of CCL17 and CCL22 from BMDMs incubated with Ena-treated neutrophils without taurine depletion further illustrated that N2-oriented switching of neutrophils caused by Ena-induced taurine

(D) Combination of ZNF460 with the promoter sequences of *GGT1* as validated by ChIP assay. $n = 3$ independent experiments.
(E) Dual luciferase reporter gene assay was conducted to evaluate the inhibitory effects of Ena on the conjunction of ZNF460 with *GGT1* promoter. $n = 3$ independent experiments.
(F) Molecular docking analysis between Ena and ZNF460.
(G) Results of cellular thermal shift assay as visualized by western blot. $n = 3$ independent experiments.
(H) Results of drug affinity responsive target stability test as visualized by western blot. $n = 3$ independent experiments.
(I) Results of pull-down assay as visualized by western blot. $n = 3$ independent experiments.
(J) Immunofluorescence staining was employed to assess the nuclear translocation of ZNF460 in HL-60 neutrophils with different treatments. Scale bar, 4 μm; $n = 3$ independent experiments.
(K) Immunofluorescence staining was employed to assess the expression of GGT1 in HL-60 neutrophils with different treatments. Scale bar, 4 μm; $n = 3$ independent experiments.
(L) After the cells underwent indicated treatments, GGT1 expression was detected. $n = 3$ independent experiments.
(M) After the cells underwent indicated treatments, taurine content was detected. $n = 3$ independent experiments.
(N) mRNA levels of *cdo*, *csad*, *fmo1*, and *baat* as measured using RT-qPCR. $n = 3$ independent experiments.
Data were shown as mean ± standard deviation (SD) from biological replicates, and statistical comparisons were performed using unpaired Student's t test in (D, G, I, and N) and one-way ANOVA followed by Tukey's multiple comparisons test in (D, E, H, and J–M). $^{*}p < 0.05$, $^{**}p < 0.01$, $^{***}p < 0.001$, $^{****}p < 0.0001$.

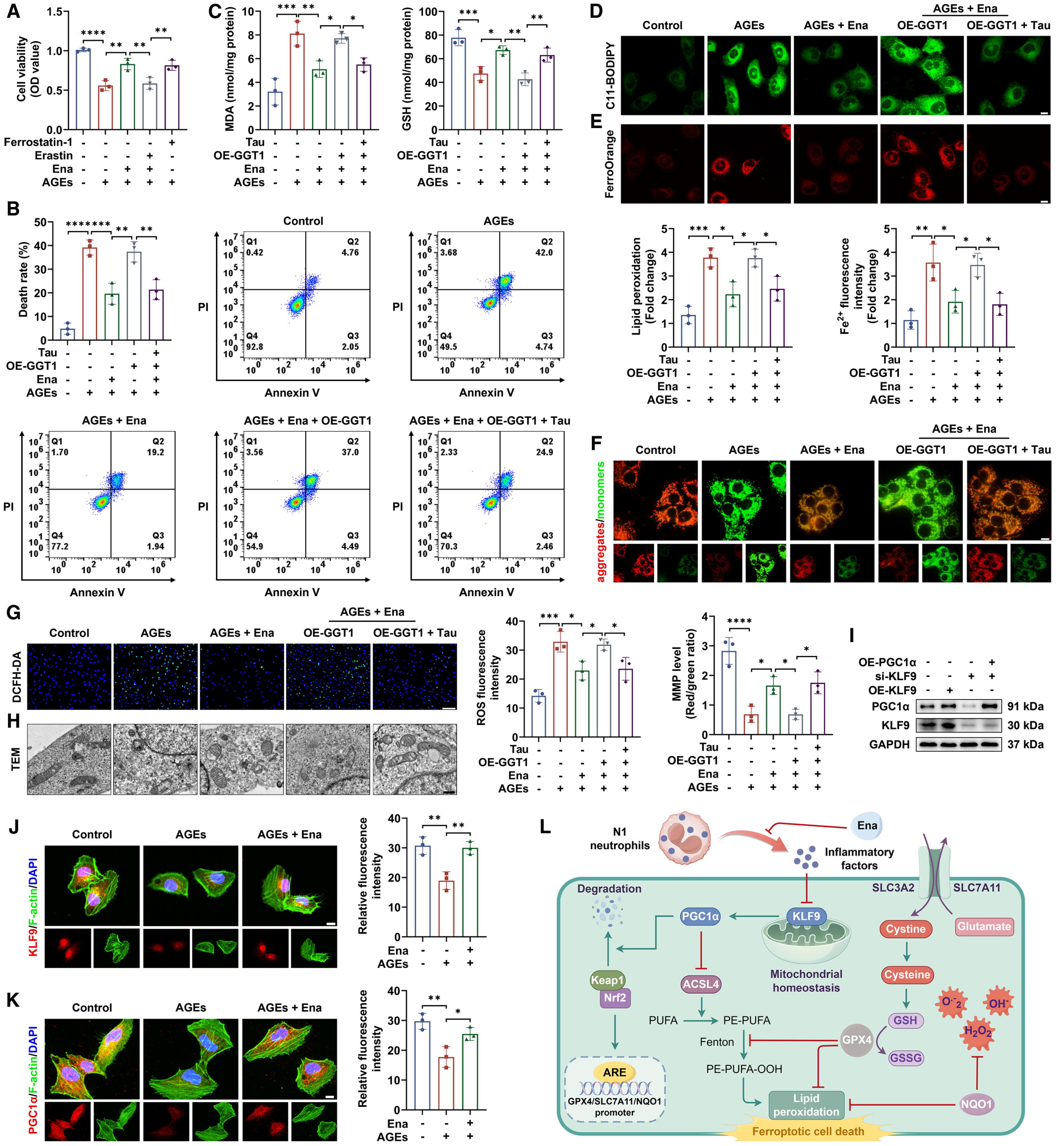

Figure 5. Inhibitory effects of Ena on HUVEC ferroptosis activated by AGE-elicited neutrophils

(A) CCK-8 assay was performed to measure the viability of HUVECs. *n* = 3 independent experiments.
(B) Death rate of HUVECs incubated with neutrophils pretreated by different strategies as detected using flow cytometry. *n* = 3 independent experiments.
(C) Levels of MDA and GSH were quantified to assess the ferroptosis activity. *n* = 3 independent experiments.
(D) Intracellular lipid peroxidation was visualized by fluorescence staining. Scale bar, 10 μm; *n* = 3 independent experiments.
(E) Fe^{2+} ion content was visualized by fluorescence staining. Scale bar, 10 μm; *n* = 3 independent experiments.
(F) JC-1 kit was used to evaluate the mitochondrial membrane potential of HUVECs. Scale bar, 10 μm; *n* = 3 independent experiments.
(G) DCFH-DA fluorescence probe was applied to determine ROS abundance in HUVECs. Scale bar, 200 μm; *n* = 3 independent experiments.
(H) Mitochondrial morphology in HUVECs as analyzed by transmission electron microscopy. Scale bar, 500 nm; *n* = 3 independent experiments.
(I) Western blot detection of PGC1α and KLF9 in HUVECs with different treatments. *n* = 3 independent experiments.

(legend continued on next page)

up-regulation was capable of providing an anti-inflammatory niche friendly to tissue proliferation (Figures S5E and S5F).[37]

Ena facilitated KLF9/PGC1α pathway activation to alleviate inflammation niche-induced endothelial cell ferroptosis

Subsequently, we found that Ena preconditioning reversed adverse effects of AGE-induced neutrophils on HUVEC viability, which was invalidated or mimicked once HUVECs were treated with ferroptosis activator erastin or inhibitor ferrostatin-1, separately (Figure 5A). Thereafter, we postulated that Ena ameliorated angiogenic dysfunction of endothelial cells incubated with inflamed neutrophils by impeding inflammation-initiated ferroptosis.[38] The flow cytometry test further validated that Ena protected HUVECs against inflammatory death in a taurine-dependent way (Figure 5B). Coincident results were seen by the quantitative analysis of lipid peroxidation indicator MDA, in contrast to the level change of anti-oxidative GSH (Figure 5C). Then, inhibitory effects of Ena-taurine axis on membrane lipid peroxidation and cytoplasmic Fe^{2+} ion accumulation were corroborated by BODIPY C11 and FerroOrange probe staining (Figures 5D and 5E). As mitochondrial dysfunction favored ferroptosis activation, we found that, via repressing taurine catabolism, Ena weakened N1-induced mitochondrial damage of HUVECs, as seen by MMP increase, ROS enrichment restraint, and morphological improvement (Figures 5F–5H).[39–42] The above data implied that angiogenic enhancement of endothelial cells was partly related to ferroptosis suppression, relying on taurine-dependent inflammation relief of adjacent neutrophils.

Krüppel-like factor 9 (KLF9) was revealed to mediate mitochondrial homeostasis via initiating the expression of peroxisome proliferator-activated receptor γ coactivator 1 α (PGC1α), a central regulator of energy metabolism (Figure 5I).[43–45] We observed a decreased level of KLF9 and PGC1α in HUVECs incubated with AGE-primed neutrophils, which was normalized by Ena preconditioning (Figures 5J and 5K). Given that PGC1α facilitated content elevation of nuclear factor E2-related factor 2 (Nrf2) responsible for transcription of genes against ferroptosis, we found that Ena-evoked neutrophils increased nuclear Nrf2 content, relying on endothelial KLF9/PGC1α axis (Figure S6A).[46] Meanwhile, acyl-CoA synthetase long-chain family 4 (ACSL4) required for lipid peroxidation possessed the opposite tendency of expression alteration (Figure S6B). Level increase of GPX4, NQO1, and SLC7A11 and content reduction of PTGS2 and Keap1 were observed when co-cultured neutrophils were pretreated with Ena, while KLF9-PGC1α inhibition in HUVECs weakened the above effects (Figures S6C and S6D). Due to the association of KLF9 with inflammation restriction, it was likely that Ena-triggered repression on disordered immune niche facilitated the activation of KLF9-PGC1α pathway, followed by enhancement of Nrf2 nuclear translocation and ACSL4 expression decrement, leading to ferroptosis inhibition (Figure 5L).[47]

Then, we investigated whether regulation of STING and ferroptosis activity affected diabetic wound repair process for initially validating the pro-healing mechanism of Ena *in vivo*. Our results revealed that H-151 treatment effectively accelerated wound closure of diabetic mice, which was reversed by erastin injection (Figure S7A). In parallel, histological examination demonstrated that compared to the control group, erastin addition markedly abolished the level increase of granular tissue thickness and collagen deposition induced by H-151 administration (Figures S7B and S7C). These findings disclosed the authenticity of STING-ferroptosis axis in mediating skin tissue regeneration, implying the potential that Ena acted as a candidate used for diabetic wound management *in vivo*. This observation was further supported in the following steps.

Preparation and characterization of TMZE

Sample morphology was assessed via scanning electron microscopy (SEM) and transmission electron microscopy (TEM), revealing the well-dispersed, uniform and regular rhombohedral shapes of MnZn-MOF and TMZE (Figure S8A). Moreover, high-angle annular dark field scanning TEM (HAADF-STEM) and elemental mapping visualized the homogeneous distribution of C, N, O, Mn, and Zn throughout the TMZE, validating the feasible introduction of Mn and Zn ions (Figure S8B). Dynamic light scattering data indicated that the particle diameter of MnZn-MOF (178.6 nm) was comparable to that of MZE (181.2 nm), but lower than that of TMZE (207.4 nm) (Figure S8C). The corresponding zeta potential values measured were 16.4, 2.3, and −21.3 mV, attributed to the encapsulation of Ena and targeting peptide functionalization (Figure S8D).

The ultraviolet-visible (UV-vis) absorption spectra uncovered that TMZE possessed a characteristic absorption peak of Ena at 258 nm, suggesting that the drug could be smoothly incorporated into the porous structure of TMZE (Figure S8E). According to the standard curve, Ena encapsulation and loading efficiency in the nanoparticles were calculated at 29.14 ± 2.25 wt % and 17.74 ± 1.88 wt %, respectively. Fourier transform infrared spectra showed that the MZE displayed a major C=C bonding absorption peak at 1,717 cm^{-1}, ascribed for the predominant incorporation of Ena within MnZn-MOF pores. For TMZE, a C=O bonding absorption peak at 1,606 cm^{-1} was exhibited, due to the stretching vibration of carboxyl bonds, confirming the successful surface modification of MZE with cFLFLFK-NH_2 (Figure S8F). X-ray photoelectron spectroscopy analysis showed the presence of Mn (663.7 eV) and Zn (1,021.5 eV) peaks in the full scan spectra of MnZn-MOF, MZE, and TMZE, which

(J) Expression of KLF9 in HUVECs incubated with neutrophils pretreated by different approaches as detected using immunofluorescence staining. Scale bar, 10 μm; *n* = 3 independent experiments.
(K) Expression of PGC1α in HUVECs incubated with neutrophils pretreated by different approaches as detected using immunofluorescence staining. Scale bar, 10 μm; *n* = 3 independent experiments.
(L) Schematic diagram of Ena-elicited alleviation on endothelial cell ferroptosis induced by pro-inflammatory neutrophils.
Data were shown as mean ± standard deviation (SD) from biological replicates, and statistical comparisons were performed using one-way ANOVA followed by Tukey's multiple comparisons test in (A–G and I–K). *$p < 0.05$, **$p < 0.01$, ***$p < 0.001$, ****$p < 0.0001$.

CellPress
OPEN ACCESS

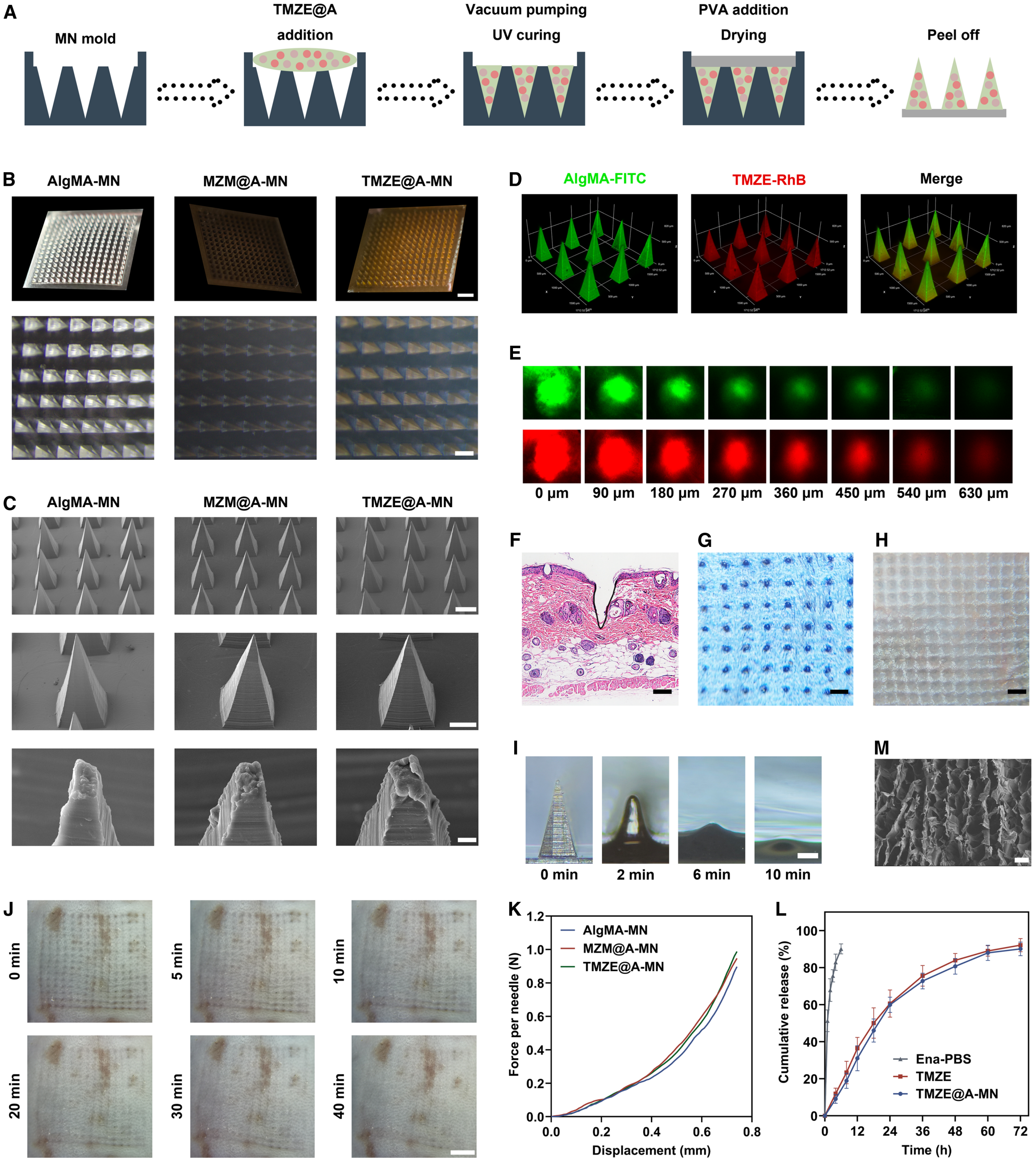

Figure 6. Characterization of AlgMA-based MN patch encapsulating Ena-load engineered MnZn-MOF

(A) Fabrication procedure of TMZE@A-MN.

(B) Overall shape of MN array comprising different constituents using a stereo microscope. Scale bars: 2 mm (upper) and 500 μm (lower); *n* = 3 independent experiments.

(C) Needle morphology as seen by SEM. Scale bars: 200 μm (upper), 100 μm (middle), and 3 μm (lower); *n* = 3 independent experiments.

(D) Fluorescence staining was adopted to visualize the distribution of TMZE within the MN. *n* = 3 independent experiments.

(E) Presence of TMZE@A-MN at different tissue layer was imaged by a confocal laser scanning microscope. *n* = 3 independent experiments.

(F) H&E staining of skin tissue following the insertion of MN patch. Scale bar, 100 μm; *n* = 3 independent experiments.

(legend continued on next page)

also verified the successful synthesis of these nanosystems (Figure S8G). Powder X-ray diffraction results displayed that these nanoparticles shared comparable sharp diffraction peaks, suggesting that loading of Ena and binding of peptide fragment did not compromise the crystalline structure of MnZn-MOF (Figure S8H).

Owing to the adverse effects of oxidative stress on diabetic wound repair, antioxidative properties of TMZE were assessed.[18] TMZE yielded a decrease in the UV-vis absorption peaks at 517 nm of DPPH· and 734 nm of $ABTS^{+}$· in a concentration-dependent manner, illustrating its potent free radical-scavenging activities (Figures S8I and S8J). The decrease in the absorbance peak of H_2O_2, $\cdot O_2^-$ and ·OH radicals also disclosed antioxidative functions of TMZE (Figures S8K–S8M). Of note, the uptake assay showed that fluorescence signal of TMZE labeled with rhodamine B (RhB) within neutrophils was markedly enhanced in contrast to that of BMDMs and HUVECs, affirming the targeting ability of MnZn-MOF endowed by the cFLFLFK-NH_2 peptide (Figure S8N). Moreover, the mixture of Ena-loaded MOF with AlgMA failed to invalidate photocuring property of the hydrogel, which guaranteed the performance of MN appearance (Figure S8O). We also found that MnZn-MOF could weaken the survival of *Escherichia coli* (*E. coli*) and methicillin-resistant *Staphylococcus aureus* (MRSA), which was not affected by Ena encapsulation and targeting peptide coupling (Figure S9).

Fabrication and manifestation of TMZE@A-MN

Through TMZE mixture with AlgMA, vacuum pumping, and ultraviolet curing, the TMZE@A-MN patch was formed (Figure 6A). Stereo microscopy indicated that the orderly 15 × 15 pyramid microarray was located on the polyvinyl alcohol (PVA) substrate (Figure 6B). The alteration of encapsulated substances had no influence on needle appearance, possessing a uniform tip height of 800 μm, base width of 360 μm, and needle spacing of 720 μm (Figure 6C). 3D visualization of the even distribution of TMZE in the needle tip validated the loading capacity of the photo-cross-linked MN (Figure 6D). Meanwhile, RhB and fluorescein isothiocyanate were seen in different skin tissue layers with confocal microscopy, suggesting that the MN patch successfully pierced through the stratum corneum and delivered Ena-loaded MnZn-MOF to deeper dermal regions (Figure 6E). Histological detection also revealed that the pinhole was formed by skin rupture after utilizing our MN (Figure 6F). Moreover, top view from excised murine and porcine back skin following MN insertion presented well-arranged microporous cavities, further confirming the robust transdermal penetration property of the prepared MN (Figures 6G and 6H).

Needle tips initiated the dissolution in the skin within 2 min, completely dissolved after insertion for 10 min, allowing TMZE to be released into local tissue and leaving the intact PVA portion for avoiding wound contamination, preventing drug loss, and absorbing wound exudate (Figure 6I). The skin of mice almost returned to its original state within 40 min upon the MN puncture, suggesting that TMZE@A-MN could not cause obvious damage and allergic reaction to the skin tissue (Figure 6J). The force-displacement curve indicated that each needle tip of AlgMA-MN could tolerate at least 0.6 N without fracture, which was similar to that of MZM@A-MN and TMZE@A-MN, demonstrating that the incorporated nanoparticle and drug did not change the compressive strength of MN, which was far greater than the reported minimum force (0.058 N) required for normal skin penetration (Figure 6K).[48] In contrast to the burst release of Ena dissolved in PBS, TMZE@A-MN exhibited a sustainable controlled release behavior, with a cumulative drug release rate of 31.09% ± 6.75%, 59.95% ± 4.11%, and 88.03% ± 4.02% at 12, 24, and 60 h, respectively (Figure 6L). More importantly, TMZE@A-MN and TMZE shared analogous release curve profiles, possibly attributed to polyporous microstructure of hydrogel (Figure 6M).

TMZE@A-MN treatment promoted diabetic wound healing in mice

Subsequently, the effects of Ena administration route alteration on wound healing process of diabetic mice were assessed *in vivo* (Figure 7A). We found that TMZE@A-MN intervention achieved faster healing extent than the other groups, reaching 94.28% on day 14. For the same period, the wound closure rates of MZE@A-MN, Ena, and MZM@A-MN were 86.8%, 76.27% and 71.82%, respectively, in contrast to that of PBS group (60.08%) (Figure 7B). Histological staining indicated that, compared to the PBS group, the granular tissue thickness increase by Ena was comparable to that of MZM@A-MN and much lower than that treated by MZE@A-MN, showing even higher level in the wound area surrounding TMZE@A-MN (Figure 7C). In parallel, both Ena and MZM@A-MN incurred much more collagen deposition than PBS, while MZE@A-MN and TMZE@A-MN were surrounded by denser collagen fibers (Figure 7D). The rising levels of these parameters relying on the administration route alteration of Ena further illustrated the contributing roles in diabetic wound repair induced by MN-mediated targeting delivery.[49]

As seen in Figure 7E, the neutrophil marker MPO was found to display abundant positive rate for the PBS and MZM@A-MN groups, which were reduced by Ena intervention. Fewer MPO-positive cells were identified when Ena was incorporated into

(G) MN-caused pinholes in the mouse back skin. Scale bars: 500 μm; $n = 3$ independent experiments.
(H) MN-caused pinholes in the porcine back skin. Scale bars: 1 mm; $n = 3$ independent experiments.
(I) Degradation trait of the MN patch after insertion into the mouse skin. Scale bar, 200 μm; $n = 3$ independent experiments.
(J) Recovery situation of mouse back skin following the MN application. Scale bar, 2 mm; $n = 3$ independent experiments.
(K) Mechanical force curves of AlgMA-MN, MZM@A-MN, and TMZE@A-MN. $n = 3$ independent experiments.
(L) Release curves of Ena in PBS, TMZE, and TMZE@A-MN. $n = 3$ independent experiments.
(M) Microstructure of AlgMA captured by SEM. Scale bar, 50 μm; $n = 3$ independent experiments.
Data were shown as mean ± standard deviation (SD) from biological replicates, and statistical comparisons were performed using one-way ANOVA followed by Tukey's multiple comparisons test in (K and L).

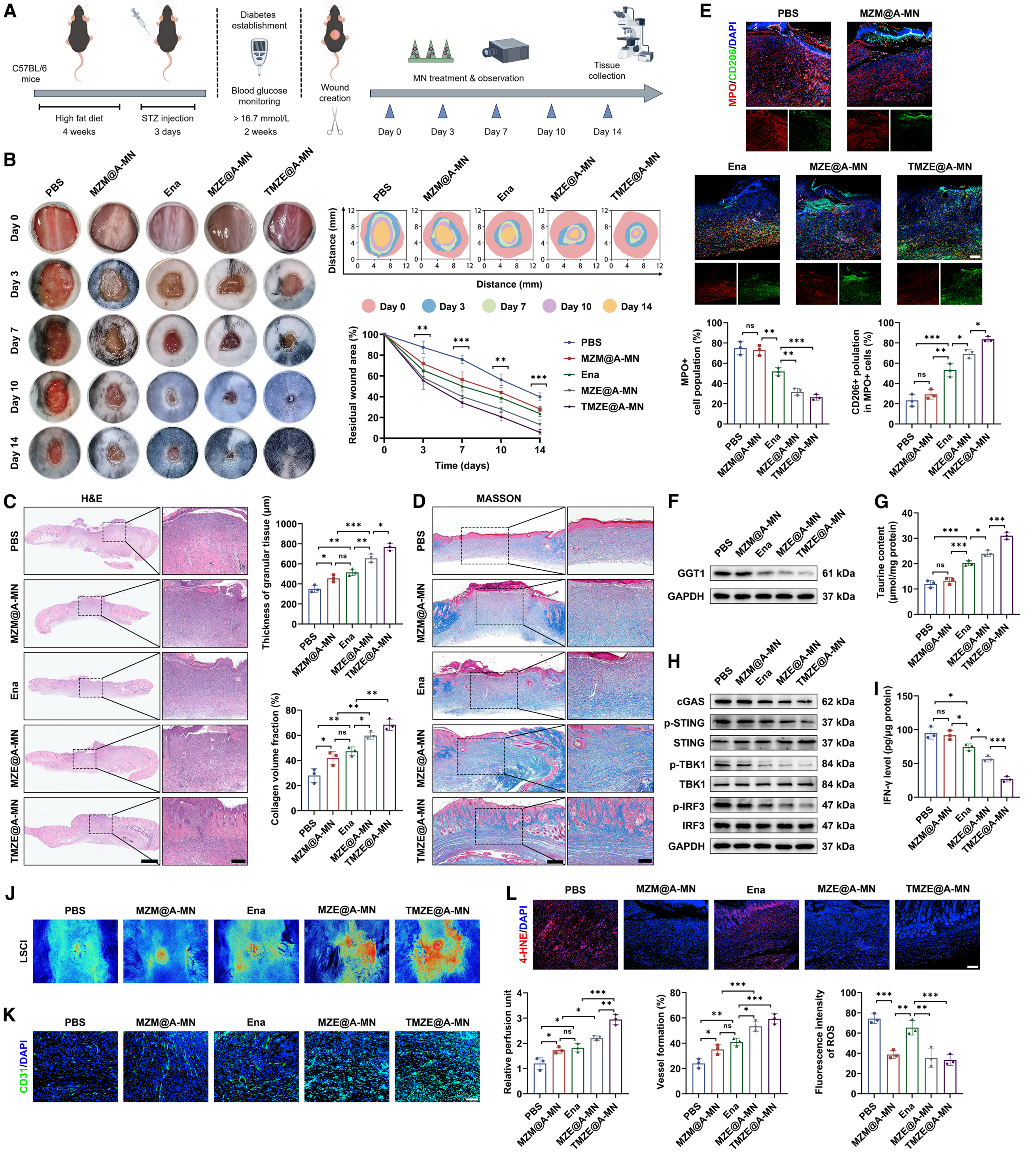

Figure 7. Therapeutic roles of TMZE@A-MN in wound repair of diabetic mice

(A) Schematic diagram demonstrating the experimental procedure of diabetic wound healing in mice.

(B) Representative images of wounds at days 0, 3, 7, 10, and 14 post-operation. $n = 4$ mice per group.

(C) Histological analysis with H&E staining of wounds following the sacrifice of mice. Scale bars: 1 mm and 200 μm; $n = 3$ biologically independent samples.

(D) Histological analysis with Masson's trichrome staining of wounds following the sacrifice of mice. Scale bars: 400 μm and 200 μm; $n = 3$ biologically independent samples.

(E) Expression of MPO and CD206 as detected in wound samples of PBS, MZM@A-MN, Ena, MZE@A-MN, and TMZE@A-MN groups. Scale bar, 200 μm; $n = 3$ biologically independent samples.

(legend continued on next page)

MnZn-MOF in the MN patch. Instead, more positive staining of CD206 among the MPO+ cells could be observed for the Ena-treated group, potentiating the drug-elicited N2 phenotype formation and neutrophil overload resolution. The expression of GGT1 and neutrophil aggregation in the wound region shared similar change behaviors exposed to different remedies, contrary to taurine level of local skin tissues, demonstrating the superiority of Ena transdermal targeting transportation *in vivo* (Figures 7F and 7G). Meanwhile, the suppressive effects of Ena on cGAS-STING pathway activity and downstream IFN-γ expression of diabetic wound tissues were accelerated by the application of MnZn-MOF carrier encapsulated in the MN patch (Figures 7H and 7I). Further characterization of macrophage phenotype manifested a lower level of iNOS and a higher expression of Arg-1 for MZE@A-MN and TMZE@A-MN compared to the Ena treatment (Figure S10A). Similar trends were also observed for inflammation-related signaling factors and cytokines of each group with indicated remedies, indicating the high efficiency of TMZE@A-MN administration improvement for reshaping the anti-inflammatory microenvironment in the diabetic wound area (Figures S10B and S10C).

Then, we found that blood flow within the skin wound tissue treated with Ena was close to the level of MZM@A-MN penetration, but dramatically lower than those by MZE@A-MN and TMZE@A-MN insertion, in contrast to the PBS group (Figure 7J). These findings were corroborated by the results of local vessel visualization, revealing the enhancement produced by this drug delivery vehicle on Ena-evoked neovascularization (Figure 7K). Then, an incremental expression of VEGFA and Ki67 was seen with the engineering improvement of Ena administration system, which might explain its beneficial roles in vessel formation in the diabetic wound tissues to some extent (Figure S11A). Consistent with the *ex vivo* results, Ena elevated the activity of KLF9/PGC1α pathway in skin wounds, yet higher pathway activity was uncovered in the TMZE@A-MN group (Figure S11B). In parallel, the repressive effects of Ena on ferroptosis activation were inferior to that yielded by Ena-loaded MN patch, which might be another mechanism underlaying its promotion on angiogenesis (Figures S11C and S11D). For evaluating the antioxidative abilities of the nanosystem within the wound area of diabetic mice, immunofluorescence staining against 4-hydroxynonenal (4-HNE) representing a sensitive biomarker of oxidative stress was performed, revealing that MOF-treated wound tissues displayed reduced level of 4-HNE when compared to those in PBS and Ena-affected wound areas (Figure 7L). Moreover, there was no difference in cellular viability and hemolysis ratio among these groups (Figures S12A–S12C). Similar body weight, blood parameters, and visceral histological characters further confirmed the good biosafety of Ena-incorporated delivery system used *in vivo* (Figures S12D–S12G).

In vivo wound healing evaluation of the porcine diabetic wound

Due to the resemblance to the subcutaneous structures and re-epithelialization-centric healing mode of human skin tissues, dorsal wounds of diabetic porcine model were created to mirror the wound status in the clinic (Figure S13A).[50,51] Herein, both MZE@A-MN- and TMZE@A-MN-treated wounds showed expedited closure rate compared to those by MZM@A-MN or Ena application alone, yet PBS injection led to the minimum wound area reduction (Figure S13B). Additionally, MN patch with Ena-loaded nanoparticles induced the thickest epidermis with complete stratification and enhanced formation of numerous rete ridge structures, while weakened effects were seen by Ena injection alone (Figure S13C). These microscopic observations and histological indicators revealed that TMZE@A-MN not only facilitated the structural regeneration but also induced functional recovery of the porcine skin tissue. Then, Ena treatment triggered level reduction of ICAM1, Fas, and GGT1 but stimulated expression increase of CXCR2, CXCR4, and taurine in diabetic porcine wound tissues, with a more conspicuous extent seen upon the adoption of transdermal drug delivery (Figures S13D–S13F). In contrast to the PBS injection, the activity of cGAS-STING pathway was repressed by Ena intervention, which displayed lower level in the targeting peptide-included group (Figure S13G). The efficacy of TMZE@A-MN against inflammatory responses in porcine wound region was further verified by analyzing inflammation cytokines and upstream signals, also affecting downstream anti-ferroptotic cascade activation (Figures S13H–S13K). Meanwhile, the level change of 4-HNE suggested the beneficial roles of drug entrance route improvement in scavenging ROS in diabetic porcine wound areas (Figure S13L).

DISCUSSION

In mammalian cells, taurine is generated from cysteine through a series of enzymatic reactions regulated by CDO, CSAD and FMO1, yet the conversion of taurine into downstream secondary metabolites is catalyzed by BAAT.[26,28,52] Herein, our results disclosed that Ena treatment induced content elevation of taurine in neutrophils, without affecting the expression of above enzymes. Multi-omics analysis uncovered that Ena-elicited suppression on the ZNF460-GGT1 axis delayed taurine transformation into 5-glutamyl-taurine, which provided an insight into endogenous secondary metabolism patterns of taurine. More importantly,

(F) GGT1 level in skin wounds of each group as quantified using western blot. $n = 3$ biologically independent samples.
(G) Content of taurine in the wound area with indicated treatments. $n = 3$ biologically independent samples.
(H) cGAS-STING pathway activity as measured with western blot. $n = 3$ biologically independent samples.
(I) IFN-γ level in the wound tissue as determined with the ELISA kit. $n = 3$ biologically independent samples.
(J) Blood flow within wound region of each group as measured by laser speckle contrast imaging. $n = 3$ biologically independent samples.
(K) Newly formed vessels in the wound area as visualized using immunofluorescence staining. Scale bar, 100 μm; $n = 3$ biologically independent samples.
(L) Level of ROS in each group was detected by immunofluorescence staining. Scale bar, 200 μm; $n = 3$ biologically independent samples.
Data were shown as mean ± standard deviation (SD) from biological replicates, and statistical comparisons were performed using one-way ANOVA followed by Tukey's multiple comparisons test in (B–L). ns: not significant, *$p < 0.05$, **$p < 0.01$, ***$p < 0.001$.

we discovered that Ena-induced increase of taurine restrained cGAS-STING cascade activity to facilitate N1 neutrophil repolarization to N2 phenotype, explaining Ena-provoked anti-inflammatory niche in favoring wound repair. Notably, given that N-acetyltaurine displayed regulatory roles in body weight control and energy control, whether 5-glutamyl-taurine, another taurine metabolite identified in this study, affected the immune status reprogramming of neutrophils was worth investigating in detail.[28] Since angiotensin II acted as a potent pro-inflammatory factor and ACE inhibition was reported to exert pivotal effects on improving the dysfunction of immune microenvironment,[53,54] there was a possibility that angiotensin II was also involved in taurine decrease of neutrophils within diabetic wound region. Thus, investigating the association of angiotensin II with ZNF-GGT1 axis regulation was worthy for further clarifying taurine-dependent anti-inflammatory activity of Ena. On account of a viewpoint derived from the previous studies revealing an interplay of cross-organ immune regulation,[55–57] elevated angiotensin II produced by activated diabetic pulmonary ACE system might enter the cutaneous wound area to exacerbate local inflammation, providing an insight into the pathogenic mechanisms of delayed healing in a lung-skin axis manner.

Dysfunctional immune microenvironment is documented to yield detrimental roles in regulating diabetic wound healing via triggering excessive death events of multiple cell types, particularly endothelial cell ferroptosis, which mainly aggravated the retardation of angiogenesis.[58,59] Our findings showed that KLF9 enhanced by Ena-provoked inflammation amelioration accelerated PGC1α expression to inhibit ferroptosis activation of HUVECs, depending on Nrf2 and ACSL4 mediation. Since down-expressed KLF9 was observed in disarranged mitochondria, there was a possibility that upregulation of KLF9 was due to mitochondrial structure restoration, by the fact that inflammation-inhibiting condition was beneficial for mitochondrial impairment improvement.[39,45] Since KLF9 deficiency led to mitophagy abrogation, which was considered susceptible to ferroptosis initiation, it was likely that KLF9-mediated mitophagic process in the PGC1α-dependent way and pro-survival effects of KLF9-PGC1α axis against ferroptosis were attributed to mitophagy activity enhancement to some extent. Intriguingly, results from another study manifested that taurine released from host anti-inflammatory macrophages entered into adjacent tumor cells via the taurine transporter for impeding ferroptosis; the direct involvement of Ena-elicited taurine elevation of neutrophils in the ferroptosis suppression of HUVECs might be rewarding.[60]

Approaches aimed at improving rapid release and low retention of drug application *in vivo* were demonstrated to be beneficial in strengthening the therapeutic efficacy.[18] Herein, we introduced the Ena-loaded engineered MnZn-MOF with neutrophil-targeted ability. By assembly in the MN patch, the nanoparticles developed transdermal delivery and enabled the drug to maintain controlled release in the deep wound tissue, which expedited immune homeostasis and triggered vessel sprout efficiently, thereby dramatically enhancing the salutary roles of Ena in wound healing. In addition, the robust antioxidative and antibacterial properties of MnZn-MOF not only endowed it with potent promotive effects on repair processes but also supported the combination therapy to be optimal, revealing enormous potential of the multifunctional TMZE on diabetic wound management.

In conclusion, our study provided evidence that abundant AGEs that existed in the diabetic niche could induce neutrophil polarization to N1 phenotype for facilitating the creation of local condition unfriendly to the wound repair process. By evoking metabolic reprogramming of taurine, Ena promoted N2 neutrophil repolarization to ameliorate ferroptosis activation of endothelial cells and favor M2 phenotype formation of macrophages. Notably, the introduction of multifunctional TMZE@A-MN patch was proven to possess beneficial effects on skin tissue recovery, emphasizing the potential of the nanosystem for application of DFU management.

Limitations of the study

Nevertheless, there were several limitations. First, although taurine was proven to alleviate mitochondrial dysfunction, mechanisms by which taurine represses dsDNA leakage from the mitochondria are still elusive.[26] Adopting the mice with specific gene interference like GGT1 and STING wound be worthy to determine the underlying molecular mechanisms that Ena accelerates diabetic wound repair *in vivo*. Moreover, considering the heterogeneity of neutrophils in the bone marrow niche, the suitability of BMDNs as the study object to analyze the roles of neutrophils might be further assessed. Additionally, the employment principle of databases across species was another thesis that remained to be validated. Meanwhile, the loading capacity and encapsulation efficiency of Ena were still needed to be heightened for abating drug loss and boosting its stability. Then, after MZE was discharged from the MN array, seeking viable approaches designed to reinforce the residence time of the composite nanomaterial in local wound area was of high priority. As the clinical samples and animal tissues used in this study were acquired from male subjects, future experiments should contain both sexes to evaluate whether the observed effects were sex specific. For further evaluating the translation feasibility of our drug delivery system in clinical application, wound healing experiments performed on other large animals like primates may be more conducive.

RESOURCE AVAILABILITY

Lead contact

Requests for further information, resources, and reagents should be directed to and will be fulfilled by the lead contact, Bobin Mi (mibobin@hust.edu.cn).

Materials availability

This study did not generate new unique reagents.

Data and code availability

- The RNA-seq data and metabolomics data have been deposited at NCBI (SRA: SRP668579) and Mendeley Data (https://data.mendeley.com/datasets/hy5v62xxpm/2) and are publicly available as of the data of publication.
- This paper does not report the original code.
- Additional data generated or analyzed during this study are included in the supplemental information files.
- Any additional information required to reanalyze the data reported in this paper is available from the lead contact upon request.

ACKNOWLEDGMENTS

This study was supported by the China Postdoctoral Science Foundation (2023M731217), and the National Natural Science Foundation of China (82372406 and 82572772). We thank the Figdraw (www.figdraw.com) for preparing Figure 5L in this manuscript and extend our gratitude to Scientific Compass (www.shiyanjia.com) for providing invaluable assistance with the preparation of the graphical abstract.

AUTHOR CONTRIBUTIONS

L.L., Y.X., and J.L. conceptualized and designed the research. Y.Q., S.Z., and Yanzhi Zhao performed the experiments, X.Z., M.W., and K.Z. analyzed the experimental data. G.W., J.Z., and F.A.-S. prepared the figures. L.L. and B.M. wrote the paper. G.L., Yanli Zhao, and B.M. supervised the project.

DECLARATION OF INTERESTS

The authors declare no competing interests.

STAR★METHODS

Detailed methods are provided in the online version of this paper and include the following:

- KEY RESOURCES TABLE
- EXPERIMENTAL MODEL AND STUDY PARTICIPANT DETAILS
  - Ethics statement
  - Dorsal skin wound model
  - Cell culture
  - Microbe strains
  - *In vivo* assessment of diabetic wound repair
- METHOD DETAILS
  - Flow cytometry
  - *In vitro* experiment for the effect of Ena on neutrophils
  - RNA sequencing
  - Metabolome analysis
  - Co-culture experiments
  - Proliferation assay
  - Cellular migration
  - Tube formation *in vitro*
  - Plasmid transfection and RNA interference
  - Chromatin immunoprecipitation (ChIP)
  - Luciferase reporter assay
  - Molecular docking
  - Cellular thermal shift assay (CETSA)
  - Drug affinity responsive target stability (DARTS) assay
  - Biotin pull-down assay
  - Taurine measurements
  - Cellular viability test
  - Ferroptosis indicator detection
  - Preparation and characterization of TMZE
  - Fabrication and characterization of TMZE@A-MN
  - Antibacterial activity
  - Wound healing marker detection
  - ELISA assay
  - Biosafety evaluation
  - Immunofluorescent detection
  - RNA extraction and quantitative real-time PCR (qRT-PCR)
  - Western blot analysis
  - Hemolytic test
- QUANTIFICATION AND STATISTICAL ANALYSIS

SUPPLEMENTAL INFORMATION

Supplemental information can be found online at https://doi.org/10.1016/j.xcrm.2026.102714.

Received: September 9, 2025
Revised: December 14, 2025
Accepted: March 1, 2026
Published: March 30, 2026

REFERENCES

1. Ruder, K. (2024). Diabetic foot infections and amputations are all too common-Here's what could move the needle. JAMA *331*, 998–1000. https://doi.org/10.1001/jama.2024.1016.
2. Xie, J., Liu, X., Wu, B., Chen, B., Song, Q., Guan, Y., Gong, Y., Yang, C., Lin, J., Huang, M., et al. (2024). Bone transport induces the release of factors with multi-tissue regenerative potential for diabetic wound healing in rats and patients. Cell Rep. Med. *5*, 101588. https://doi.org/10.1016/j.xcrm.2024.101588.
3. He, W., Yan, L., Hu, D., Hao, J., Liou, Y.C., and Luo, G. (2025). Neutrophil heterogeneity and plasticity: Unveiling the multifaceted roles in health and disease. MedComm *6*, e70063. https://doi.org/10.1002/mco2.70063.
4. Jerome, A.D., Sas, A.R., Wang, Y., Hammond, L.A., Wen, J., Atkinson, J.R., Webb, A., Liu, T., and Segal, B.M. (2024). Cytokine polarized, alternatively activated bone marrow neutrophils drive axon regeneration. Nat. Immunol. *25*, 957–968. https://doi.org/10.1038/s41590-024-01836-7.
5. Wu, J., Gao, P., Yang, C., Zhuang, F., Luo, Y., Wen, F., Zhang, P., Wang, L., Xie, H., Dai, C., et al. (2025). Targeting mitochondrial complex I of CD177+ neutrophils alleviates lung ischemia-reperfusion injury. Cell Rep. Med. *6*, 102140. https://doi.org/10.1016/j.xcrm.2025.102140.
6. Ge, M., Zhu, W., Mei, J., Hu, T., Yang, C., Lin, H., and Shi, J. (2025). Piezoelectric-enhanced nanocatalysts trigger neutrophil N1 polarization against bacterial biofilm by disrupting redox homeostasis. Adv. Mater. *37*, e2409633. https://doi.org/10.1002/adma.202409633.
7. Liu, Y., Sun, Q., Guo, J., Yan, L., Yan, Y., Gong, Y., Lin, J., Yuan, H., Jin, J., Wang, B., et al. (2025). Dual ferroptosis induction in N2-TANs and TNBC cells via FTH1 targeting: A therapeutic strategy for triple-negative breast cancer. Cell Rep. Med. *6*, 101915. https://doi.org/10.1016/j.xcrm.2024.101915.
8. Lentini, G., Famà, A., De Gaetano, G.V., Coppolino, F., Mahjoub, A.K., Ryan, L., Lien, E., Espevik, T., Beninati, C., and Teti, G. (2023). Caspase-8 inhibition improves the outcome of bacterial infections in mice by promoting neutrophil activation. Cell Rep. Med. *4*, 101098. https://doi.org/10.1016/j.xcrm.2023.101098.
9. Phillipson, M., and Kubes, P. (2019). The healing power of neutrophils. Trends Immunol. *40*, 635–647. https://doi.org/10.1016/j.it.2019.05.001.
10. Fan, Y., Yang, J., Xie, Y., Yang, X., Zhu, H., Liu, Y., Xia, Z., Ji, S., and Yang, R. (2025). Inflammatory memory-activated biomimetic nanovesicles regulate neutrophil plasticity and metabolic reprogramming for rapid diabetic wound healing via targeting miR-193a-5p/TLR4/JNK/P38 MAPK pathways. J. Nanobiotechnol. *23*, 115. https://doi.org/10.1186/s12951-025-03193-5.
11. Carroll, K.R., Mizrachi, M., Simmons, S., Toz, B., Kowal, C., Wingard, J., Tehrani, N., Zarfeshani, A., Kello, N., El Khoury, L., et al. (2024). Lupus autoantibodies initiate neuroinflammation sustained by continuous HMGB1:RAGE signaling and reversed by increased LAIR-1 expression. Nat. Immunol. *25*, 671–681. https://doi.org/10.1038/s41590-024-01772-6.
12. Amir, O., Elbaz-Greener, G., Carasso, S., Claggett, B., Barbarash, O., Zaman, A., Christersson, C., Kiatchoosakun, S., Anonuevo, J., Opolski, G., et al. (2024). Association between body mass index and clinical outcomes in patients with acute myocardial infarction and reduced systolic function: Analysis of PARADISE-MI trial data. Eur. J. Heart Fail. *27*, 558–565. https://doi.org/10.1002/ejhf.3542.
13. Qiu, X., Li, G., Wan, W., Li, J., Yan, G., Wang, S., Hu, X., Han, Z., Shan, Y., Zhou, Y., et al. (2026). Targeted 2-deoxy-D-ribose delivery by biomimetic nanoplatform activates EGFR for accelerated heart valve endothelialization. Adv. Sci. *13*, e14170. https://doi.org/10.1002/advs.202514170.

14. Kawamura, S., Yoneyama, Y., Saiki, N., Wu, Y., Moriya, C., Ohmura, R., Maezawa, M., Shimada, Y., Wang, Y., Mori, K., et al. (2025). Modeling antithymocyte globulin-induced microvasculopathy using human iPSC-derived vascularized liver organoids. Cell Rep. Med. *6*, 102433. https://doi.org/10.1016/j.xcrm.2025.102433.

15. Cao, D.-Y., Saito, S., Veiras, L.C., Okwan-Duodu, D., Bernstein, E.A., Giani, J.F., Bernstein, K.E., and Khan, Z. (2020). Role of angiotensin-converting enzyme in myeloid cell immune responses. Cell. Mol. Biol. Lett. *25*, 31. https://doi.org/10.1186/s11658-020-00225-w.

16. van Raalte, D.H., Bjornstad, P., Cherney, D.Z.I., de Boer, I.H., Fioretto, P., Gordin, D., Persson, F., Rosas, S.E., Rossing, P., Schaub, J.A., et al. (2024). Combination therapy for kidney disease in people with diabetes mellitus. Nat. Rev. Nephrol. *20*, 433–446. https://doi.org/10.1038/s41581-024-00827-z.

17. Linnane, E., Haddad, S., Melle, F., Mei, Z., and Fairen-Jimenez, D. (2022). The uptake of metal-organic frameworks: A journey into the cell. Chem. Soc. Rev. *51*, 6065–6086. https://doi.org/10.1039/d0cs01414a.

18. Xiong, Y., Feng, Q., Lu, L., Qiu, X., Knoedler, S., Panayi, A.C., Jiang, D., Rinkevich, Y., Lin, Z., Mi, B., et al. (2024). Metal-organic frameworks and their composites for chronic wound healing: From bench to bedside. Adv. Mater. *36*, e2302587. https://doi.org/10.1002/adma.202302587.

19. Wang, D., Yao, H., Ye, J., Gao, Y., Cong, H., and Yu, B. (2024). Metal-organic frameworks (MOFs): Classification, synthesis, modification, and biomedical applications. Small *20*, e2404350. https://doi.org/10.1002/smll.202404350.

20. Martin, M.S., Jacob-Dolan, J.W., Pham, V.T.T., Sjoblom, N.M., and Scheck, R.A. (2025). The chemical language of protein glycation. Nat. Chem. Biol. *21*, 324–336. https://doi.org/10.1038/s41589-024-01644-y.

21. Schalkwijk, C.G., Micali, L.R., and Wouters, K. (2023). Advanced glycation endproducts in diabetes-related macrovascular complications: Focus on methylglyoxal. Trends Endocrinol. Metab. *34*, 49–60. https://doi.org/10.1016/j.tem.2022.11.004.

22. Gao, Q., Jiang, Y., Zhou, D., Li, G., Han, Y., Yang, J., Xu, K., Jing, Y., Bai, L., Geng, Z., et al. (2024). Advanced glycation end products mediate biomineralization disorder in diabetic bone disease. Cell Rep. Med. *5*, 101694. https://doi.org/10.1016/j.xcrm.2024.101694.

23. Sharifiaghdam, M., Shaabani, E., Faridi-Majidi, R., De Smedt, S.C., Braeckmans, K., and Fraire, J.C. (2022). Macrophages as a therapeutic target to promote diabetic wound healing. Mol. Ther. *30*, 2891–2908. https://doi.org/10.1016/j.ymthe.2022.07.016.

24. NaveenKumar, S.K., Hemshekhar, M., Jagadish, S., Manikanta, K., Vishalakshi, G.J., Kemparaju, K., and Girish, K.S. (2020). Melatonin restores neutrophil functions and prevents apoptosis amid dysfunctional glutathione redox system. J. Pineal Res. *69*, e12676. https://doi.org/10.1111/jpi.12676.

25. Cui, J., Xu, Z., Yu, Z., Zhang, Q., Liu, S., Du, B., Gan, L., Yan, C., Xue, G., Feng, J., et al. (2025). High-alcohol-producing Klebsiella pneumoniae aggravates lung injury by affecting neutrophils and the airway epithelium. Cell Rep. Med. *6*, 101886. https://doi.org/10.1016/j.xcrm.2024.101886.

26. Singh, P., Gollapalli, K., Mangiola, S., Schranner, D., Yusuf, M.A., Chamoli, M., Shi, S.L., Lopes Bastos, B., Nair, T., Riermeier, A., et al. (2023). Taurine deficiency as a driver of aging. Science *380*, eabn9257. https://doi.org/10.1126/science.abn9257.

27. Izquierdo, J.M. (2024). Taurine as a possible therapy for immunosenescence and inflammaging. Cell. Mol. Immunol. *21*, 3–5. https://doi.org/10.1038/s41423-023-01062-5.

28. Wei, W., Lyu, X., Markhard, A.L., Fu, S., Mardjuki, R.E., Cavanagh, P.E., Zeng, X., Rajniak, J., Lu, N., Xiao, S., et al. (2024). PTER is a N-acetyltaurine hydrolase that regulates feeding and obesity. Nature *633*, 182–188. https://doi.org/10.1038/s41586-024-07801-6.

29. Zhang, B., Xu, P., and Ablasser, A. (2025). Regulation of the cGAS-STING pathway. Annu. Rev. Immunol. *43*, 667–692. https://doi.org/10.1146/annurev-immunol-101721-032910.

30. Zhang, Z., and Zhang, C. (2025). Regulation of cGAS-STING signalling and its diversity of cellular outcomes. Nat. Rev. Immunol. *25*, 425–444. https://doi.org/10.1038/s41577-024-01112-7.

31. Shang, L., Jiang, X., Zhao, X., Huang, X., Wang, X., Jiang, X., Kong, X., Yao, M., Jiang, S., and Wong, P.-P. (2024). Mitochondrial DNA-boosted dendritic cell-based nanovaccination triggers antitumor immunity in lung and pancreatic cancers. Cell Rep. Med. *5*, 101648. https://doi.org/10.1016/j.xcrm.2024.101648.

32. Bi, W., Li, X., Jiang, Y., Gao, T., Zhao, H., Han, Q., and Zhang, J. (2025). Tumor-derived exosomes induce neutrophil infiltration and reprogramming to promote T-cell exhaustion in hepatocellular carcinoma. Theranostics *15*, 2852–2869. https://doi.org/10.7150/thno.104557.

33. Chung, J.Y.F., Tang, P.C.T., Chan, M.K.K., Xue, V.W., Huang, X.R., Ng, C.S.H., Zhang, D., Leung, K.T., Wong, C.K., Lee, T.L., et al. (2023). Smad3 is essential for polarization of tumor-associated neutrophils in non-small cell lung carcinoma. Nat. Commun. *14*, 1794. https://doi.org/10.1038/s41467-023-37515-8.

34. Mihlan, M., Wissmann, S., Gavrilov, A., Kaltenbach, L., Britz, M., Franke, K., Hummel, B., Imle, A., Suzuki, R., Stecher, M., et al. (2024). Neutrophil trapping and nexocytosis, mast cell-mediated processes for inflammatory signal relay. Cell *187*, 5316–5335.e28. https://doi.org/10.1016/j.cell.2024.07.014.

35. Horckmans, M., Ring, L., Duchene, J., Santovito, D., Schloss, M.J., Drechsler, M., Weber, C., Soehnlein, O., and Steffens, S. (2017). Neutrophils orchestrate post-myocardial infarction healing by polarizing macrophages towards a reparative phenotype. Eur. Heart J. *38*, 187–197. https://doi.org/10.1093/eurheartj/ehw002.

36. Ma, S., Sun, B., Duan, S., Han, J., Barr, T., Zhang, J., Bissonnette, M.B., Kortylewski, M., He, C., Chen, J., et al. (2023). YTHDF2 orchestrates tumor-associated macrophage reprogramming and controls antitumor immunity through CD8(+) T cells. Nat. Immunol. *24*, 255–266. https://doi.org/10.1038/s41590-022-01398-6.

37. Scortegagna, M., Hockemeyer, K., Dolgalev, I., Poźniak, J., Rambow, F., Li, Y., Feng, Y., Tinoco, R., Otero, D.C., Zhang, T., et al. (2020). Siah2 control of T-regulatory cells limits anti-tumor immunity. Nat. Commun. *11*, 99. https://doi.org/10.1038/s41467-019-13826-7.

38. Tang, D., Chen, X., Kang, R., and Kroemer, G. (2021). Ferroptosis: Molecular mechanisms and health implications. Cell Res. *31*, 107–125. https://doi.org/10.1038/s41422-020-00441-1.

39. Glover, H.L., Schreiner, A., Dewson, G., and Tait, S.W.G. (2024). Mitochondria and cell death. Nat. Cell Biol. *26*, 1434–1446. https://doi.org/10.1038/s41556-024-01429-4.

40. Zhang, Z., Zhou, H., Gu, W., Wei, Y., Mou, S., Wang, Y., Zhang, J., and Zhong, Q. (2024). CGI1746 targets σ_1R to modulate ferroptosis through mitochondria-associated membranes. Nat. Chem. Biol. *20*, 699–709. https://doi.org/10.1038/s41589-023-01512-1.

41. Dixon, S.J., and Olzmann, J.A. (2024). The cell biology of ferroptosis. Nat. Rev. Mol. Cell Biol. *25*, 424–442. https://doi.org/10.1038/s41580-024-00703-5.

42. Zheng, J., Wang, Q., Chen, J., Cai, G., Zhang, Z., Zou, H., Zou, J.X., Liu, Q., Ji, S., Shao, G., et al. (2024). Tumor mitochondrial oxidative phosphorylation stimulated by the nuclear receptor RORγ represents an effective therapeutic opportunity in osteosarcoma. Cell Rep. Med. *5*, 101519. https://doi.org/10.1016/j.xcrm.2024.101519.

43. Fan, H., Zhang, Y., Zhang, J., Yao, Q., Song, Y., Shen, Q., Lin, J., Gao, Y., Wang, X., Zhang, L., et al. (2020). Cold-inducible klf9 regulates thermogenesis of brown and beige fat. Diabetes *69*, 2603–2618. https://doi.org/10.2337/db19-1153.

44. Cui, A., Fan, H., Zhang, Y., Zhang, Y., Niu, D., Liu, S., Liu, Q., Ma, W., Shen, Z., Shen, L., et al. (2019). Dexamethasone-induced Kruppel-like factor 9 expression promotes hepatic gluconeogenesis and hyperglycemia. J. Clin. Investig. *129*, 2266–2278. https://doi.org/10.1172/JCI66062.

45. Zhang, L., Zhang, M., Huang, J., Huang, J., Zhang, Y., Zhang, Y., Chen, H., Wang, C., Xi, X., Fan, H., et al. (2024). Klf9 is essential for cardiac mitochondrial homeostasis. Nat. Cardiovasc. Res. *3*, 1318–1336. https://doi.org/10.1038/s44161-024-00561-6.

46. Jamwal, S., Blackburn, J.K., and Elsworth, J.D. (2021). PPARgamma/PGC1alpha signaling as a potential therapeutic target for mitochondrial biogenesis in neurodegenerative disorders. Pharmacol. Ther. *219*, 107705. https://doi.org/10.1016/j.pharmthera.2020.107705.

47. Zhang, Y., Du, C., Wang, W., Qiao, W., Li, Y., Zhang, Y., Sheng, S., Zhou, X., Zhang, L., Fan, H., et al. (2024). Glucocorticoids increase adiposity by stimulating Kruppel-like factor 9 expression in macrophages. Nat. Commun. *15*, 1190. https://doi.org/10.1038/s41467-024-45477-8.

48. Li, S., Wang, X., Yan, Z., Wang, T., Chen, Z., Song, H., and Zheng, Y. (2023). Microneedle patches with antimicrobial and immunomodulating properties for infected wound healing. Adv. Sci. *10*, e2300576. https://doi.org/10.1002/advs.202300576.

49. Song, Y., You, Y., Xu, X., Lu, J., Huang, X., Zhang, J., Zhu, L., Hu, J., Wu, X., Xu, X., et al. (2023). Adipose-derived mesenchymal stem cell-derived exosomes biopotentiated extracellular matrix hydrogels accelerate diabetic wound healing and skin regeneration. Adv. Sci. *10*, e2304023. https://doi.org/10.1002/advs.202304023.

50. Theocharidis, G., Yuk, H., Roh, H., Wang, L., Mezghani, I., Wu, J., Kafanas, A., Contreras, M., Sumpio, B., Li, Z., et al. (2022). A strain-programmed patch for the healing of diabetic wounds. Nat. Biomed. Eng. *6*, 1118–1133. https://doi.org/10.1038/s41551-022-00905-2.

51. Wei, T., Pan, T., Peng, X., Zhang, M., Guo, R., Guo, Y., Mei, X., Zhang, Y., Qi, J., Dong, F., et al. (2024). Janus liposozyme for the modulation of redox and immune homeostasis in infected diabetic wounds. Nat. Nanotechnol. *19*, 1178–1189. https://doi.org/10.1038/s41565-024-01660-y.

52. Cao, T., Zhang, W., Wang, Q., Wang, C., Ma, W., Zhang, C., Ge, M., Tian, M., Yu, J., Jiao, A., et al. (2024). Cancer SLC6A6-mediated taurine uptake transactivates immune checkpoint genes and induces exhaustion in CD8(+) T cells. Cell *187*, 2288–2304.e27. https://doi.org/10.1016/j.cell.2024.03.011.

53. Zhang, T., Zhong, X.C., Feng, Z.X., Lin, X.Y., Chen, C.Y., Wang, X.W., Guo, K., Wang, Y., Chen, J., Du, Y.Z., et al. (2025). An active shrinkage and antioxidative hydrogel with biomimetic mechanics functions modulates inflammation and fibrosis to promote skin regeneration. Bioact. Mater. *45*, 322–344. https://doi.org/10.1016/j.bioactmat.2024.11.028.

54. Dong, X., Fan, J., Lin, D., Wang, X., Kuang, H., Gong, L., Chen, C., Jiang, J., Xia, N., He, D., et al. (2022). Captopril alleviates epilepsy and cognitive impairment by attenuation of C3-mediated inflammation and synaptic phagocytosis. J. Neuroinflammation *19*, 226. https://doi.org/10.1186/s12974-022-02587-8.

55. Yu, D., Gao, X., Shao, F., Liu, Z., Liu, A., Zhao, M., Tang, Z., Guan, Y., and Wang, S. (2025). Antigen-presenting innate lymphoid cells induced by BCG vaccination promote a respiratory antiviral immune response through the skin−lung axis. Cell. Mol. Immunol. *22*, 390–402. https://doi.org/10.1038/s41423-025-01267-w.

56. Dou, J., Jiang, J., Xue, Y., Jiang, X., Jiang, Y., Xiao, P., and Xu, J. (2025). The interplay of cross-organ immune regulation in inflammation and cancer. MedComm *6*, e70249. https://doi.org/10.1002/mco2.70249.

57. Herman-Edelstein, M., Guetta, T., Barnea, A., Waldman, M., Ben-Dor, N., Barac, Y.D., Kornowski, R., Arad, M., Hochhauser, E., and Aravot, D. (2021). Expression of the SARS-CoV-2 receptorACE2 in human heart is associated with uncontrolled diabetes, obesity, and activation of the renin angiotensin system. Cardiovasc. Diabetol. *20*, 90. https://doi.org/10.1186/s12933-021-01275-w.

58. Lv, D., Cao, X., Zhong, L., Dong, Y., Xu, Z., Rong, Y., Xu, H., Wang, Z., Yang, H., Yin, R., et al. (2023). Targeting phenylpyruvate restrains excessive NLRP3 inflammasome activation and pathological inflammation in diabetic wound healing. Cell Rep. Med. *4*, 101129. https://doi.org/10.1016/j.xcrm.2023.101129.

59. Lin, C., Hu, Y., Lin, Z., Du, L., Hu, Y., Ouyang, L., Xie, X., Cheng, P., Liao, J., Lu, L., et al. (2025). MMP-9 responsive hydrogel promotes diabetic wound healing by suppressing ferroptosis of endothelial cells. Bioact. Mater. *43*, 240–254. https://doi.org/10.1016/j.bioactmat.2024.09.006.

60. Xiao, H., Du, X., Tao, Z., Jing, N., Bao, S., Gao, W.Q., Dong, B., and Fang, Y.X. (2024). Taurine inhibits ferroptosis mediated by the crosstalk between tumor cells and tumor-associated macrophages in prostate cancer. Adv. Sci. *11*, e2303894. https://doi.org/10.1002/advs.202303894.

STAR★METHODS

KEY RESOURCES TABLE

REAGENT or RESOURCE	SOURCE	IDENTIFIER
Antibodies		
APC anti-human CD16	Biolegend	Cat#360705; RRID: AB_2562750
APC anti-mouse Ly6G	Biolegend	Cat#127613; RRID: AB_1877163
APC anti-mouse F4/80	Biolegend	Cat#123115; RRID: AB_893493
FITC anti-human CD66b	Biolegend	Cat#305103; RRID: AB_314495
FITC anti-mouse CD11b	Biolegend	Cat#101205; RRID: AB_312788
Mouse monoclonal anti-Arg-1	Proteintech	Cat#66129-1-Ig; RRID: AB_2881528
Mouse monoclonal anti-GAPDH	Proteintech	Cat#60004-1-Ig; RRID: AB_2107436
Mouse monoclonal anti-GPX4	Proteintech	Cat#67763-1-Ig; RRID: AB_2909469
Mouse monoclonal anti-MPO	Proteintech	Cat#66177-1-Ig; RRID: AB_2881572
Mouse monoclonal anti-GGT1	Abcam	Cat#ab55138; RRID: AB_941759
Mouse monoclonal anti-dsDNA	Santa Cruz Biotechnology	Cat#sc-58749; RRID: AB_783088
Mouse monoclonal anti-Fas	Santa Cruz Biotechnology	Cat#sc-21730; RRID: AB_627220
Mouse monoclonal anti-Keap1	Santa Cruz Biotechnology	Cat#sc-514914; RRID: AB_2861131
Mouse monoclonal anti-KLF9	Santa Cruz Biotechnology	Cat#sc-376422; RRID: AB_11151402
Mouse monoclonal anti-NQO1	Santa Cruz Biotechnology	Cat#sc-376023; RRID: AB_10987895
Mouse monoclonal anti-PGC1α	Santa Cruz Biotechnology	Cat#sc-518025; RRID: AB_2890187
Mouse Monoclonal anti-STAT3	Cell Signaling Technology	Cat#9139; RRID: AB_331757
PE anti-mouse CD163	Biolegend	Cat#111803; RRID: AB_2936729
PE anti-human CD206	Biolegend	Cat#321105; RRID: AB_571910
PerCP/Cyanine5.5 anti-mouse CD80	Biolegend	Cat#104721; RRID: AB_893406
PerCP/Cyanine5.5 anti-mouse CD206	Biolegend	Cat#141715; RRID: AB_2561991
Rabbit monoclonal anti-ACSL4	Abclonal	Cat#A20414; RRID: AB_2909505
Rabbit polyclonal anti-CCL17	Abclonal	Cat#A2854; RRID: AB_2764679
Rabbit monoclonal anti-CD31	Abclonal	Cat#A19014; RRID: AB_2862506
Rabbit polyclonal anti-cGAS	Abclonal	Cat#A8335; RRID: AB_2770305
Rabbit polyclonal anti-CXCL10	Abclonal	Cat#A19138; RRID: AB_2862631
Rabbit polyclonal anti-CXCR2	Abclonal	Cat#A3301; RRID: AB_2769086
Rabbit polyclonal anti-ICAM1	Abclonal	Cat#A5597; RRID: AB_2766365
Rabbit polyclonal anti-IFN-γ	Abclonal	Cat#A12450; RRID: AB_2759294
Rabbit polyclonal anti-PTGS2	Abclonal	Cat#A1253; RRID: AB_2759370
Rabbit monoclonal anti-4-HNE	Abclonal	Cat#A26085; RRID: AB_3718713
Rabbit polyclonal anti-SLC7A11	Abclonal	Cat#A13685; RRID: AB_2760546
Rabbit polyclonal anti-VEGFA	Abclonal	Cat#A0280; RRID: AB_2757092
Rabbit polyclonal anti-Histone H3	Proteintech	Cat#17168-1-AP; RRID: AB_2716755
Rabbit monoclonal anti-NRF2	Proteintech	Cat#80593-1-RR; RRID: AB_2918904
Rabbit monoclonal anti-TGF-β1	Proteintech	Cat#81746-2-RR; RRID: AB_3670503
Rabbit polyclonal anti-ZNF460	Proteintech	Cat#25299-1-AP; RRID: AB_2880016
Rabbit polyclonal anti-CD206	Proteintech	Cat#18704-1-AP; RRID: AB_10597232
Rabbit polyclonal anti-AGE	Abcam	Cat#ab23722; RRID: AB_447638
Rabbit monoclonal anti-CXCR4	Abcam	Cat#ab181020; RRID: AB_2910168
Rabbit monoclonal anti-iNOS	Abcam	Cat#ab283655; RRID: AB_3083470
Rabbit polyclonal anti-Ki67	Abcam	Cat#ab15580; RRID: AB_443209
Rabbit monoclonal anti-IRF3	Cell Signaling Technology	Cat#11904; RRID: AB_2722521

(Continued on next page)

Continued

REAGENT or RESOURCE	SOURCE	IDENTIFIER
Rabbit monoclonal anti-NF-κB	Cell Signaling Technology	Cat#8242; RRID: AB_10859369
Rabbit monoclonal anti-*p*-IRF3	Cell Signaling Technology	Cat#29047; RRID: AB_2773013
Rabbit monoclonal anti-p-NF-κB	Cell Signaling Technology	Cat#3033; RRID: AB_331284
Rabbit monoclonal anti-Smad3	Cell Signaling Technology	Cat#9523; RRID: AB_2193182
Rabbit monoclonal anti-STAT1	Cell Signaling Technology	Cat#14994; RRID: AB_2737027
Rabbit monoclonal anti-*p*-Smad3	Cell Signaling Technology	Cat#9520; RRID: AB_2193207
Rabbit monoclonal anti-p-STAT1	Cell Signaling Technology	Cat#9167; RRID: AB_561284
Rabbit monoclonal anti-p-STAT3	Cell Signaling Technology	Cat#9145; RRID: AB_2491009
Rabbit monoclonal anti-STING	Cell Signaling Technology	Cat#13647; RRID: AB_2732796
Rabbit monoclonal anti-TBK1	Cell Signaling Technology	Cat#3504; RRID: AB_2255663
Rabbit monoclonal anti-p-STING	Cell Signaling Technology	Cat#19781; RRID: AB_2737062
Rabbit monoclonal anti-*p*-TBK1	Cell Signaling Technology	Cat#5483; RRID: AB_10693472
Bacterial and virus strains		
Escherichia coli	ATCC	ATCC 25922
Methicillin-resistant *Staphylococcus aureus*	ATCC	ATCC 33591
Biological samples		
Human skin wound tissues	Union Hospital, Tongji Medical College, Huazhong University of Science and Technology	N/A
Chemicals, peptides, and recombinant proteins		
Alginate methacryloyl	Engineering for Life	N/A
AGE-BSA	Abcam	Cat#ab51995
cFLFLFK-NH2 peptide	BioAct Peptide Biotech	N/A
Enalaprilat	MedChemExpress	Cat#HY-B0231
Erastin	MedChemExpress	Cat#HY-15763
Ferrostatin-1	MedChemExpress	Cat#HY-100579
H-151	MedChemExpress	Cat#HY-112693
Lipofectamine 3000	Invitrogen	Cat#L3000015
Lipofectamine RNAiMAX	Invitrogen	Cat#13778150
M-CSF	PeproTech	Cat#315-02-10UG
$Mn(NO_3)_2 \cdot 4H_2O$	Sigma-Aldrich	Cat#935697-50G
Phosphatase and protease inhibitor	MedChemExpress	Cat#HY-K0013
Phalloidin	Sigma-Aldrich	Cat#P5282
Polyvinyl alcohol	Engineering for Life	N/A
Pronase	Sigma-Aldrich	Cat#P5147
RIPA	Boster	Cat#AR0102
Streptavidin, agarose beads	Millipore	Cat#16-126
Streptozotocin	Sigma-Aldrich	Cat#S0130
Taurine	Sigma-Aldrich	Cat#T0625
TRIzol	Invitrogen	Cat#15596026
$Zn(NO_3)_2 \cdot 6H_2O$	Sigma-Aldrich	Cat#228737-100G
Critical commercial assays		
Annexin V-FITC/PI apoptosis kit	Beyotime	Cat#C1062S
BCA assay kit	Beyotime	Cat#P0010
BODIPY 581/591 C11 assay kit	Sigma-Aldrich	Cat#SML3717
Calcein/PI cell viability kit	Beyotime	Cat#C2015S
CCK-8 assay kit	Dojindo	Cat#CK04
ChIP assay kit	Beyotime	Cat#P2078
Dual-luciferase reporter assay kit	Promega	Cat#E1910

(*Continued on next page*)

Continued

REAGENT or RESOURCE	SOURCE	IDENTIFIER
EdU cell proliferation kit	Beyotime	Cat#C0078S
FerroOrange assay kit	Dojindo	Cat#F374
Hiscript III reverse transcriptase kit	Vazyme	Cat#R302-01
Matrigel matrix	Corning	Cat#356234
Mitochondrial membrane potential assay kit	Beyotime	Cat#C2006
Mouse TNF-α ELISA kit	Boster	Cat#EK0527
Mouse IL-4 ELISA kit	Boster	Cat#EK0405
Mouse IFN-γ ELISA kit	Boster	Cat#EK0375
Mouse CCL5 ELISA kit	NeoBioscience	Cat#EMC106
Mouse CCL22 ELISA kit	NeoBioscience	Cat#EMC105
Mouse bone marrow neutrophil isolation kit	Tbdscience	Cat#TBD2013NM
Nuclear and cytoplasmic protein extraction kit	Beyotime	Cat#P0027
Reactive oxygen species assay kit	Beyotime	Cat#S0033S
Taurine detection assay kit	Cell Biolabs	Cat#MET-5071
Taq Pro U$^+$ multiple probe qPCR mix kit	Vazyme	Cat#QN213-01
Deposited data		
Metabolomics data	This paper	Mendeley data: https://data.mendeley.com/datasets/hy5v62xxpm/2
RNA sequencing	This paper	SRA: SRP668579
Experimental models: Cell lines		
HL-60	ATCC	Cat#CCL-240
HUVEC	ATCC	Cat#CRL-1730
Experimental models: Organisms/strains		
Bama mini pigs	Hubei Aofei Biotechnology	N/A
C57BL/6 mice	Hubei Biont Biological Technology	N/A
Oligonucleotides		
Primers for qPCR	This paper	N/A
siRNA sequence	This paper	N/A
Recombinant DNA		
pcDNA3.1-ZNF460	GeneChem	N/A
pcDNA3.1-GGT1	GeneChem	N/A
pcDNA3.1-KLF9	GeneChem	N/A
pcDNA3.1-PGC1α	GeneChem	N/A
Software and algorithms		
AutoDock Vina	Scripps	Version 1.1.2
Cutadapt	Illumina	Version 1.15
FlowJo	BD Biosciences	Version 10.8.1
GraphPad Prism	GraphPad	Version 9.5.1
ImageJ	NIH	Version 1.54f
OriginPro 2025	OriginLab	Version 10.2.0.188
Pymol	Schrödinger	Version 2.3.4

EXPERIMENTAL MODEL AND STUDY PARTICIPANT DETAILS

Ethics statement

The human skin wound samples used in this study were obtained from diabetic patients who had ulcers below the ankle that persisted for more than one month without healing and individuals underwent urgent reconstruction surgery because of foot injury without other underlying diseases at Department of Orthopedics, Union Hospital, Tongji Medical College, Huazhong University of Science and Technology (Table S1). Our study protocol was approved by the Institutional Review Board at Union Hospital, Tongji

Medical College, Huazhong University of Science and Technology (Permit number: UHCT-IEC-SOP-016-03-01) and carried out in accordance with the Declaration of Helsinki. Written informed consent was obtained from each donor included in this study.

All the animal experiments were performed in line with the National Institutes of Health guidelines for the care and use of laboratory animals. The study protocol involving the use of mice was approved by the Institutional Animal Care and Use Committee of Huazhong University of Science and Technology (Permit number: IACUC No. 3935). The research proposal involving the use of mini pigs was approved by the Laboratory Animal Welfare & Ethics Committee of Hubei Yizhicheng Biotechnology Co., Ltd. (Permit number: IACUC No. 202302006).

Dorsal skin wound model

Male C57BL/6 mice aged 6 weeks were acquired from Hubei Biont Biological Technology Co., Ltd. Wuhan, China. To establish the type II diabetes model, mice were fed with high-fat diet for 4 weeks, accompanied by 3 consecutive days of intraperitoneal injection of STZ (60 mg/kg). Then, mice with random blood glucose higher than 16.7 mmol/L for 2 weeks were considered diabetic. Both of the normal mice and diabetic mice were anesthetized with 1% pentobarbital sodium (5 μL/g) and circular 10 mm full-thickness cutaneous wounds were generated on the dorsal region. The wound closure rate was quantified using the ImageJ software based on the digital photographs captured on the day of operation and the indicated days thereafter. Mice were euthanized at day 10 and skin wound tissues were harvested and subjected to immunofluorescent and flow cytometry analyses.

Cell culture

Neutrophil cell line (HL-60) and human umbilical vein endothelial cell (HUVEC) were gained from the American Type Culture Collection (ATCC) and grown at 37°C with 5% CO_2. The culture medium consisted of RPMI 1640 containing 10% FBS and 1% penicillin-streptomycin solution.

Bone marrow-derived neutrophils (BMDNs) and macrophages (BMDMs) were isolated from C57BL/6 mice at the age of 6 weeks using density gradient centrifugation. Briefly, femurs and tibiae were collected once the mice were sacrificed, and cell suspensions were flushed out from the marrow cavity, which were then layered over the Bone Marrow Neutrophil Isolation Kit. After the purification procedure, murine neutrophils were identified by flow cytometry and cultured in DMEM/F12 medium at a density of 2×10^6 cells per mL. For the acquirement of BMDMs, cells discharged from the marrow cavity were filtered and seeded in a 10 cm culture dish enveloped by DMEM/F12 medium comprising 50 ng/mL M-CSF. Undergoing the committed differentiation at 37°C in a 5% CO_2 atmosphere over 5 days, primary macrophages were developed and applied for the next step.

Microbe strains

Gram-negative *Escherichia coli* (E. coli) and Gram-positive methicillin-resistance *Staphylococcus aureus* (MRSA) were obtained from ATCC and inoculated into a sterilized Luria-Bertani (LB) liquid medium at 37°C under aerobic conditions.

In vivo assessment of diabetic wound repair

Following the establishment of diabetes model and creation of dorsal wounds, all mice were randomly divided into the control, H-151 and H-151 + Erastin groups. At day 0, 3, 7 and 10 post-surgery, digital images of wounds were acquired and treatments in each group were carried out. At day 14 post-wounding, the mice were euthanized and tissues were harvested for histological detection. Wound areas were measured by ImageJ and residual wound size percentage was calculated as follows: [wound area at a certain day/wound area at day 0] × 100%. Moreover, for assessing the effects of the drug delivery system on wound healing, the C57BL/6 mice with diabetic wounds were randomly allocated into 5 groups with different interventions: PBS, MZM@A-MN, Ena, MZE@A-MN and TMZE@A-MN. Then, on the day 14 post-surgery, all mice were sacrificed and tissues and blood samples were collected for the next step.

The male Bama mini pigs aged at 6 months were purchased from Hubei Aofei Biotechnology Co., Ltd. Porcine diabetes was induced by 6 months of high fat diet feeding accompanied by intravenous injection of STZ (75 mg/kg) once a week sequentially. One week after the second STZ administration, pigs exhibiting fasting blood glucose levels ≥11.1 mM and sustained for more than 10 days were considered as diabetes. Afterward, animals were anesthetized with a combined regimen of ketamine (10 mg/kg), atropine (0.05 mg/kg) and diazepam (1 mg/kg) and then maintained with 1–2% isoflurane throughout the surgery. Both sides of dorsum regions were clipped and cleaned with 70% alcohol and povidone-iodine, which were marked with individual squares using a sterile surgical marker, accompanied by creation of dermal full-thickness excisional wounds (approximately 2 cm × 2 cm × 0.6 cm) with skin forceps and double-blade cutting scissors. Subsequently, the wounds were treated with PBS, MZM@A-MN, Ena, MZE@A-MN or TMZE@A-MN every 3 days and each wound as imaged on days 0, 7, 14 and 21 for calculating the relative wound area using ImageJ software. Then, at day 21 post-surgery, porcine skin wounds were collected for histological analysis and gene expression measurements.

METHOD DETAILS

Flow cytometry

Fresh skin tissues were cut into pieces on the ice and then incubated with collagenase-IV and DNase-I at 37°C in a continuously overturned manner for 1 h. The mixture was filtered through a 70-μm cell strainer to obtain a single-cell suspension, which was centrifuged

and washed with PBS, accompanied by intervention of Fc receptor blockade (anti-human CD16 (302001, BioLegend) for clinical samples, and anti-mouse CD16/32 (101301, BioLegend) for animal samples). For the evaluation of BMDNs and BMDMs, cells were seeded in 6-well plates and experienced indicated treatments prior to staining processes. Thereafter, cells were harvested and stained with fluorescent dye-conjugated antibodies against cytomembrane proteins at 4°C in the dark for 15 min. For intracellular markers, cells were then fixed and penetrated using Cytofix/Cytoperm reagent at 4°C for 20 min, subsequently rinsed and stained with corresponding antibodies at 4°C without light for 15 min. After that, cell suspension was washed and analyzed with BD FACSCelesta flow cytometer and the data were processed using FlowJo software.

In vitro experiment for the effect of Ena on neutrophils

BMDNs were plated at a density of 2×10^6 cells per well in 6-well culture plates and divided into 3 groups with different treatments: Control, AGEs (150 μg/mL), AGEs (150 μg/mL) + Ena (10 μM). Following 24 h of incubation, cells were gathered for determining the polarization phenotype and inflammation profiles.

RNA sequencing

Total RNA from AGEs-stimulated BMDNs with or without Ena intervention was extracted using TRIzol according to the manufacturer's protocols and the isolated RNA purity and quality were assessed using a NanoDrop NC-2000 spectrophotometer (Thermo Fisher Scientific). Adopting poly-T oligo-attached magnetic beads, mRNA with polyA was specifically captured and segmented into short fragments by magnesium ions under elevated temperature, which were then reverse-transcribed to synthesize cDNA strands using random oligonucleotides. After purification, end repair and adenylation of the 3′ ends, obtained cDNA fragments with a final size of 400–500 bp were amplified and purified with the AMPure XP system (Beckman Coulter) to produce RNA-seq libraries. The transcriptome sequencing was conducted by Bioprofile Biotechnology Co., Ltd. (Shanghai, China) using an Illumina Novaseq 6000 platform following the vendor's recommendations. Cutadapt (v1.15) software was employed to filter the sequencing data to remove low-quality sequence reads. After reference genome comparisons and expression standardization, difference of gene expression was analyzed by DESeq (1.30.0) between two groups and differentially expressed genes (DEGs) possessed a p-value <0.05 and |log2FoldChange| > 1. Gene Ontology (GO) and Kyoto Encyclopedia of Genes and Genomes (KEGG) pathway databases as well as gene set enrichment analysis (GSEA) were applied to reveal the functional enrichment of these DEGs.

Metabolome analysis

The nontargeted metabolomics procedure was performed by Bioprofile Biotechnology Co., Ltd. (Shanghai, China) using a UPLC-ESI-Q-Orbitrap-MS system (UHPLC, Shimadzu Nexera X2 LC-30AD) coupled with Q-Exactive Plus (Thermo Fisher Scientific). Metabolites within neutrophils were extracted using 1 mL precooled mixtures of methanol, acetonitrile and water (v/v/v: 2:2:1) and then placed for 1 h ultrasonic shaking in ice baths. Then, the mixture was centrifuged to obtained supernatant, which in turn was concentrated to dryness in vacuum, followed by filtration with a disposable 0.22 μm cellulose acetate and transferring into 2 mL HPLC vials. For liquid chromatography (LC) separation, samples were analyzed using a ACQUITY UPLC HSS T3 column (2.1 × 100 mm, 1.8 μm, Waters). The raw MS data were processed using MS-DIAL for peak alignment, retention time correction and peak area extraction. The metabolites were identified by accuracy mass (mass tolerance <10 ppm) and MS/MS data (mass tolerance <0.02 Da), which were matched with HMDB, massbank and other public databases and our self-built metabolite standard library. R (version:4.0.3) and R packages were used for all multivariate data analyses and modeling. The variable importance on projection (VIP) score value indicates the contribution of a variable to the discrimination between all the classes of samples. Metabolites with VIP values >1.0, fold change ≥2 or ≤0.5 and p value <0.05 were considered to be statistically significant metabolites. Fold change was calculated as the logarithm of the average mass response (area) ratio between two arbitrary classes. To identify the perturbed biological pathways, the differential metabolite data were performed by KEGG pathway analysis using KEGG database.

Co-culture experiments

Exposure to the treatment with or without AGEs and Ena for 24 h, HL-60 cells were transferred to the upper chamber of the co-culture system and the HUVECs at exponential growth phase were seeded in the lower chamber. Following 24 h of co-incubation, HUVECs were harvested for further assessing the angiogenic properties. In addition, BMDMs were co-cultured with the preconditioned neutrophils and then were collected for evaluating the phenotype features and inflammation activities using flow cytometry and immunofluorescent staining.

Proliferation assay

HUVECs (200 μL) at a density of 2×10^5/mL were added to the lower chamber of a 24-well plate. Upon the incubation in the microenvironment affected by neutrophils in the upper chamber, HUVECs were stained with a 5-ethynyl-2′-deoxyuridine (EdU) kit and proliferated cells were visualized using a fluorescence-inverted microscope (Olympus).

Cellular migration

In the wound scratch test, HUVECs were seeded in the 6-well plate at a density of 1×10^6/mL. Uniform linear scratches were established with the tip of a 200 μL pipette, and images were captured by an inverted optical microscope (Olympus) after 24 h of

co-incubation. Meanwhile, for the transwell assay, 2×10^4 HUVECs (200 μL of serum-free RPMI 1640 per well) were added to the upper chamber of a 24-well plate, and neutrophil-loaded RPMI 1640 medium (600 μL) containing 10% FBS were added to the lower chamber. Following 24 h of co-culture, HUVECs moved to the lower chamber were fixed and stained with crystalline violet and then photographed with an inverted optical microscope (Olympus).

Tube formation *in vitro*

HUVECs were planted at a density of 1×10^5 cells per well in the lower chamber of a 24-well plate precoated with Matrigel matrix (200 μL). Tube formation was quantified 6 h after co-incubation by analyzing the sprouting tube-like structures with ImageJ software.

Plasmid transfection and RNA interference

Expression vectors encoding ZNF460, GGT1, KLF9 and PGC1α were constructed by GeneChem (Shanghai, China) via cloning the open reading frames of the indicated genes into a pcDNA3.1 plasmid vector. Transiently transfection of plasmids into cells for overexpression was performed with Lipofectamine 3000 reagent as per the manufacturer's instructions. siRNAs against ZNF460, GGT1 and KLF9 were synthesized by GenePharma (Shanghai, China) and their sequences were presented in Table S2. Cells were seeded in a 6-well plate and transfected with the siRNA oligonucleotides using Reduced-Serum Medium (Opti-MEM, Gibco) and Lipofectamine RNAiMAX in accordance with the manufacturer's protocols. ZNF460 and GGT1 vector plasmids and siRNAs were used for regulating gene expression in HL-60 neutrophils, while others were applied to be transferred into HUVECs separately.

Chromatin immunoprecipitation (ChIP)

A ChIP assay kit was used to perform the ChIP assay according to the manufacturer's recommended guidelines. Briefly, following the indicated treatments, HL-60 cells were cross-linked with 1% formaldehyde at 37°C for 10 min and the reaction was quenched with glycine solution. Then, neutrophils were collected and incubated with ChIP lysis buffer, accompanied by sonication for chromatin shearing on ice. For ZNF460 immunoprecipitation, equal aliquots of chromatin protein complexes were incubated with anti-ZNF460 antibody or isotype-matched control IgG at 4°C overnight. After washing three times, cellular lysates were treated with protein A/G-conjugated agarose beads and were subjected to the reversal of DNA-protein cross-linking, by which the enriched DNA fragments were purified and then determined using qRT-PCR. The sequences of primers used in the ChIP assay were shown in Table S3.

Luciferase reporter assay

The wild-type, mutant response element 1 (RE1) or mutant RE2 of promoter fragments of GGT1 were cloned upstream of the firefly luciferase reporter in a pGL3-Basic vector (Genecreate, Wuhan, China). HL-60 neutrophils seeded in a 24-well plate were co-transfected with the indicated pGL3-promoter plasmid and a pRL-SV40 Renilla luciferase reporter plasmid using lipofectamine 3000. After 48 h, cells were harvested and firefly and Renilla luciferase activities were detected by a dual luciferase reporter assay system. Moreover, after the co-transfection was completed stably, HL-60 cells were treated with different concentrations of Ena in the presence of ZNF460 upregulation for 24 h. Afterward, neutrophils were collected for measuring the luciferase activity using a multifunctional enzyme marking instrument (Biotek). The Renilla luciferase plasmid was set as an internal control.

Molecular docking

AutoDock Vina was used to analyze the binding affinity and mode of interaction between Ena and ZNF460 protein. The chemical structure of Ena was retrieved from the PubChem database (http://pubchem.ncbi.nlm.nih.gov/) in SDF file and converted to the PDBQT format for serving as a ligand. The crystal structure of ZNF460 was downloaded from the RCSD Protein DataBank (PDB, https://www.rcsb.org/) and saved in a PDB format. The protein file was prepared as a receptor by all water molecule and ligand elimination, polar hydrogen atom addition and charge insertion. The docking model was then visualized by pymol software.

Cellular thermal shift assay (CETSA)

In brief, HL-60 neutrophils were exposed to Ena (10 μM) or solvent control (DMSO) for 12 h and harvested using PBS supplemented with protease and phosphatase inhibitors. The collected cells underwent thermal denaturation at various temperature intervals ranging from 45°C to 63°C using a PCR instrument for 3 min, accompanied by three cycles of freeze-thawing with liquid nitrogen and centrifugation at 20,000 g at 4°C for 15 min. The protein level in obtained supernatants was analyzed by western blotting.

Drug affinity responsive target stability (DARTS) assay

HL-60 cells were treated with RIPA lysis buffer and then centrifuged to collect the supernatant lysates, which were incubated with different dosages of Ena at room temperature for 2 h. Subsequently, the lysates were proteolyzed with pronase (2.5 μg/mL) or treated with DMSO at 37°C for 20 min. Finally, reactions were terminated after adding the cocktail and the immunoblot was employed to quantify the expression of ZNF460.

Biotin pull-down assay

The lysates from HL-60 neutrophils were divided into two aliquots, followed by incubation with Biotin-Ena (50 μM) synthesized by Zoonbio Biotechnology (Nanjing, China) or free biotin at 4°C overnight, separately. The combined proteins were precipitated by the streptavidin-conjugated beads at 4°C for additional 2 h. Afterward, the beads were extensively washed with PBS and boiled with SDS loading buffer with the reducing agent, and then the supernatants were collected for western blot analysis.

Taurine measurements

For detection of taurine in HL-60 cells, murine and porcine skin wounds, samples were measured using a taurine assay kit according to the manufacturer's instructions. Pretreated neutrophils or wound tissues were homogenized in cold PBS via ultrasonic processing and centrifuged to remove debris, and a BCA test was employed to measure the protein level in the supernatant. Then, the homogenate was blended in taurine reaction buffer and kept at room temperature for 30 min. After the stop solution was added, the absorbance values were read at 405 nm using a microplate reader. Taurine concentration in each sample was calculated by a standard curve produced by fitting the absorbance values of taurine standards.

Cellular viability test

Under the stimulation produced by neutrophils pretreated with or without AGEs (150 μg/mL) and Ena (10 μM), HUVECs were treated with or without ferrostatin-1 (5 μM) or erastin (10 μM) for 24 h. Afterward, cell counting kit-8 (CCK-8) reagent was added to the culture medium and kept for 30 min, which in turn was gathered for the absorbance measurement using a multifunctional enzyme marking instrument (Biotek).

Additionally, HL-60 cells were divided into 5 groups: Control, AGEs, AGEs + Ena, AGEs + Ena + GGT1 overexpression (plasmid pretreatment, OE-GGT1), and AGEs + Ena + GGT1 + Taurine (50 μM). Following 24 h of intervention, neutrophils were co-incubated with HUVECs for another 24 h, and then the HUVECs were collected and subjected to Annexin-V/PI staining for assessing survival rate using flow cytometry.

Ferroptosis indicator detection

Initially, HL-60 cells were received indicated treatments for 24 h and then were transferred to the upper chamber of a 24-well plate, where HUVECs were planted in the bottom chamber. HUVECs were cultured in the micro-niche affected by the neutrophils for 24 h, followed by quantification of MDA and GSH, and then examination of lipid peroxidation and intracellular ferrous ions using C11-BODIPY and FerroOrange probes respectively. Subsequently, the mitochondrial membrane potential and reactive oxygen species (ROS) level in HUVECs were evaluated by JC-1 kit and DCFH-DA probe using a Nikon confocal laser scanning microscope. Furthermore, transmission electron microscopy (TEM, Zeiss) imaging was performed to determine the mitochondrial morphology within HUVECs.

Preparation and characterization of TMZE

Firstly, $Zn(NO_3)_2 \cdot 6H_2O$ and $Mn(NO_3)_2 \cdot 4H_2O$ were added to the methanol solution containing 2-methylimidazole, followed by vigorous stirring at room temperature for 20 h. Then, the mixture was centrifuged at 12,000 rpm for 15 min and washed three times with dimethylformamide (DMF), and the resulting MnZn-MOF nanoparticles were resuspended in DMF for the further application.

To obtain Ena-loaded MnZn-MOF (MZE), Ena was mixed with the MOF at a mass ratio of 1:1, and the mixture was stirred at room temperature overnight. After the reaction, the solution was centrifuged at 10,000 rpm for 20 min. The supernatant was discarded and the pellets were washed three time with DMF and double distilled water (ddH_2O). Subsequently, obtained MZE and cinnamyl-F-(D)L-F (cFLFLFK)-NH_2 targeting neutrophil formyl peptide receptor (mass ratio of 1:5) in tetrahydrofuran were added into ddH_2O dropwise under agitation and maintained in a fume hood overnight to evaporate the organic solvent. Afterward, the synthesized TMZE nanoparticles were purified via dialysis and washed three times with ddH_2O and stored at 4°C for the following use.

Then, SEM (Zeiss) and TEM (Zeiss) were adopted to characterize the micro-morphology of TMZE. The elemental mapping images of TMZE were obtained by a Tecnai G2 F30 instrument (FEI). Dynamic light scattering and ζ potential of the nanoparticles mentioned above were determined with Malvern Zeta Sizer Nano series. Moreover, their crystalline structures and surface features were analyzed by powder X-ray diffractometry (XRD, Bruker D2 PHASER). X-ray photoelectron spectroscopy (XPS, Thermo Fisher Scientific) was performed to reveal the elemental composition and chemical bonding. In addition, the absorbance spectra and infrared spectra of the nanoparticles were recorded on a UV-vis spectrophotometer (METASH) and a Fourier transform infrared spectrometer (Thermo Fisher Scientific), respectively. A series of free radical scavenging assays were performed to identify the antioxidant capacities of TMZE in accordance with the manufacturer's protocols. For verifying the neutrophil-targeted property of the nanoparticles, rhodamine B (RhB)-coupled TMZE was treated with BMDNs, BMDMs or HUVECs. After 6 h of incubation, cells were harvested and subjected to fluorescence detection.

Fabrication and characterization of TMZE@A-MN

To prepare the microneedle patch, lyophilized alginate methacryloyl (AlgMA) was dissolved in PBS at an appropriate concentration with the photoinitiator phenyllithium-2,4,6-trimethylbenzoylphosphonate (LAP), followed by ultrasound-mediated uniform dispersion of TMZE into the solution to form a 5% AlgMA pre-hydrogel (0.5% LAP). Then, TMZE-encapsulated AlgMA solution (TMZE@A) was injected as the hydrogel tip into a polydimethylsiloxane (PDMS) microneedle mold, which was then placed in a vacuum condition for

3 min to fill the microcavities. After the elimination of surface bubbles, the mold was dried at 35°C for 5 h to condense the solution. The vacuum de-bubbling and drying procedures were repeated twice, accompanied by formation of TMZE@A-MN with photo-crosslinking irradiation under the 405 nm UV. Afterward, polyvinyl alcohol (PVA, 20% w/v) was added as the backing layer solution to the model bottom and experienced vacuum pumping and drying processes. Following the quiescence at 35°C for 12 h, the MN patch was demolded for further usage.

A stereoscopic microscope (Olympus) was employed to visualize the overall appearance of the MN array carrying different components. The microcosmic structures of AlgMA hydrogel and MN patches were recorded using SEM (Zeiss). To measure the distribution of TMZE in the pyramid, the fluorescence staining was performed and detected with a Nikon confocal laser scanning microscope. Apart from the fluorescent measurement, the transdermal delivery ability of TMZE@A-MN was further identified by the histological analysis of H&E staining. Moreover, mouse and porcine skin tissues on the back were used for the insertion test and the mechanical strength of the MN loading diverse constituents was determined using an electronic universal testing instrument (WH-70). To ascertain the biodegradability of TMZE@A-MN and its minimal skin damage *in vivo*, the MN patch was applied to mice on the back skin tissue without hair shielding, and then the needle tip and skin surface were photographed at different time intervals. Additionally, the release of Ena from the delivery vehicle was quantified by recording the absorbance of soaked PBS solution using a UV-vis spectrophotometer according to a standard curve.

Antibacterial activity

The colony count method was performed to evaluate the antibacterial performance of MnZn-MOF. E. coli and MRSA were seeded into LB medium and then placed in a horizontal shaker in a 150 rpm overnight to achieve the desired concentration. Both bacteria were quantified by measuring the optical density at 600 nm with a spectrophotometer in accordance with the established standard curve. Then, E. coli or MRSA (500 μL of 1×10^8 CFU/mL) was added into 24-well culture plates, accompanied by supplement of Ena, MnZn-MOF or TMZE. The control was an LB medium containing bacteria treated with PBS. Following 12 h of incubation under aerobic conditions at 37°C, the supernatants from the plates were serially diluted and evenly coated on LB agar plates, and allowed to grow. After a further 12 h of incubation at 37°C, images were recorded and the antibacterial properties of each group were assessed by counting the number of colonies formed on the plates. Following the indicated treatments, E. coli and MRSA were washed with PBS, fixed with 4% paraformaldehyde and then subjected to be dehydrated by grade ethanol solutions. The morphologies of dried bacteria were photographed with SEM (Zeiss).

Wound healing marker detection

Obtained animal tissues were fixed in 4% paraformaldehyde at 4°C for 24 h. Subsequently, samples were embedded in paraffin and cross-sectioned for evaluating re-epithelialization, granular tissue formation and collagen deposition via hematoxylin & eosin (H&E) and Masson's trichrome staining. For visualizing the blood flow in skin wound area at day 10 post-surgery, the laser speckle contrast imaging (LSCI) was performed.

ELISA assay

Following the indicated treatments, culture medium of HL-60 cells and BMDMs was harvested to detect the contents of IFN-γ, TNF-α, IL-4, VEGFA, CCL5 and CCL22 using the ELISA kits according to the manufacturer's guidelines.

Upon the termination of animal experiment, the skin wounds of mice were collected and subjected to the ELISA assay for measuring the expression of inflammation-related cytokines.

Biosafety evaluation

For detecting the safety of the MOF nanoparticle-loaded MN delivery vehicle *in vitro*, HL-60 neutrophils, BMDMs and HUVECs were treated with PBS, MZM@A-MN, Ena, MZE@A-MN or TMZE@A-MN. After 24 h of incubation, cells were harvested and used for viability examination by CCK-8 assay and calcein/PI staining.

Moreover, at the endpoint of the animal experiment, blood samples were collected and subjected to the blood routine and biochemical index examination for determining the biosafety of the drug delivery system *in vivo*.

Immunofluorescent detection

Wound tissues embedded in paraffin were cross-sectioned to 5-μm-thick slices, which were subjected to dewaxing and antigen retrieval. After cells underwent indicated treatments, the culture medium was discarded and cells were washed and fixed with 4% paraformaldehyde for 15 min. Then, tissue sections and fixed cells were permeabilized by 0.2% Triton X-100 in PBS for 15 min, followed by blockade with 5% BSA solution at room temperature for 1 h and incubation with primary antibodies at 4°C overnight. After three times washing with PBS, samples were stained with appropriate fluorescence-conjugated secondary antibodies at room temperature for 2 h. Finally, the nuclei were stained with DAPI for 15 min. Fluorescent signals were captured by a Nikon confocal laser scanning microscope.

RNA extraction and quantitative real-time PCR (qRT-PCR)

Both of wound tissues and treated cells were lysed with TRIzol reagent to extract the total RNA based on the manufacturer's instructions. After the RNA concentration was measured, the cDNA was generated using Hiscript III reverse transcriptase kit. Quantitative real-time PCR was performed with the Taq Pro U^+ multiple probe qPCR mix on a Roche LightCycler 96 System. Data were analyzed by $\Delta\Delta$Ct method. GAPDH was set as the internal reference gene. The sequences of primers were listed in Table S4.

Western blot analysis

Tissue and cell lysates were prepared using RIPA buffer containing phosphatase and protease inhibitor. The nuclear protein from skin wounds was extracted using Nuclear and Cytoplasmic Protein Extraction kit. Isolated proteins were quantified with the BCA assay kit and proteins at equal mass were separated on the SDS polyacrylamide gel and then transferred onto polyvinylidene difluoride (PVDF) membrane (Millipore), accompanied by incubation with primary antibodies at 4°C overnight. Following rinse with tris buffered saline-tween solution, the membrane was incubated with horse radish peroxidase-conjugated secondary antibodies. Immunoblot bands were visualized using ECL enhanced chemiluminescence substrate on a ChemiDoc system (Bio-Rad). Signal intensity was quantified with ImageJ software via background subtraction.

Hemolytic test

Fresh blood from C57BL/6 mice was used for the hemolysis assay via measuring hemoglobin release from red blood cells (RBCs). The mouse blood was centrifuged at 1500 rpm for 5 min to precipitate erythrocytes and discard supernatant. After washing with PBS, RBCs were mixed with indicator solutions and allowed to incubate at 37°C for 1 h. The mixture was then centrifuged and supernatant absorbance was determined at 540 nm through a spectrophotometer. Treatment of ddH_2O was set as the positive control.

QUANTIFICATION AND STATISTICAL ANALYSIS

The data included in this study were presented as the mean ± standard deviation (SD). Statistical analyses were performed using GraphPad Prism. All experiments were repeated at least three times. A two-tailed, unpaired Student's *t* test was used to compare significant differences between the two groups. For multiple groups with one variable, one-way analysis of variance (ANOVA) followed by post hoc Tukey's test was adopted to determine the differences. The *p* value <0.05 was considered statistically significant. *$p < 0.05$, **$p < 0.01$, ***$p < 0.001$, ****$p < 0.0001$.

Cell Reports Medicine, Volume *7*

Supplemental information

Enalaprilat reverses neutrophil polarization imbalance via targeting taurine-STING axis for treatment of diabetic wounds

Li Lu, Yuan Xiong, Jiewen Liao, Juan Zhou, Guangji Wang, Yating Qin, Shengming Zhang, Yanzhi Zhao, Xiaodan Zhong, Mengwen Wang, Kangkang Zha, Fawwaz Al-Smadi, Guohui Liu, Yanli Zhao, and Bobin Mi

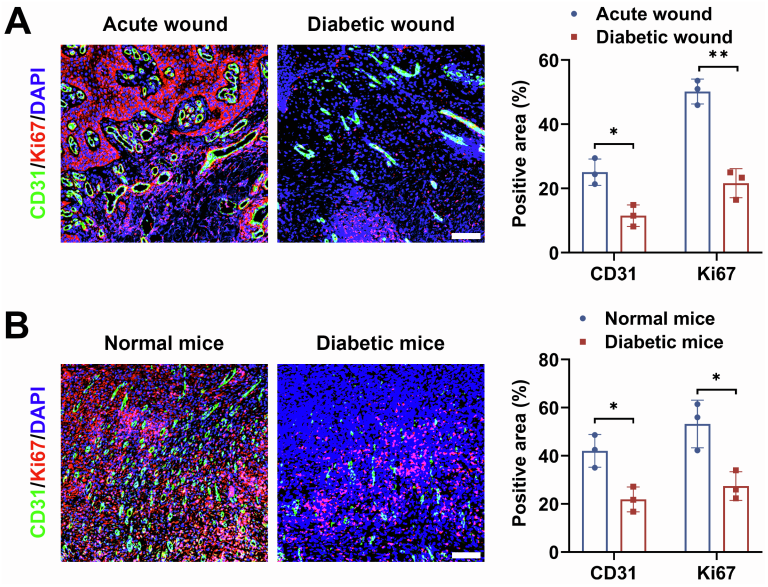

Figure S1. New vessel formation in the wound area of clinical samples and animal samples. Related to Figure 1.

(A) Representative immunofluorescence staining images of CD31 and Ki67 levels in clinical specimens of diabetic wounds and acute wounds. Scale bar: 100 µm; n = 3 biologically independent samples.

(B) Representative immunofluorescence staining images of CD31 and Ki67 levels in murine diabetic wounds and acute wounds. Scale bar: 200 µm; n = 3 biologically independent samples.

Data were shown as mean ± standard deviation (S.D.) from biological replicates and statistical comparisons were performed using unpaired Student's t test in A and B. *$P < 0.05$, **$P < 0.01$.

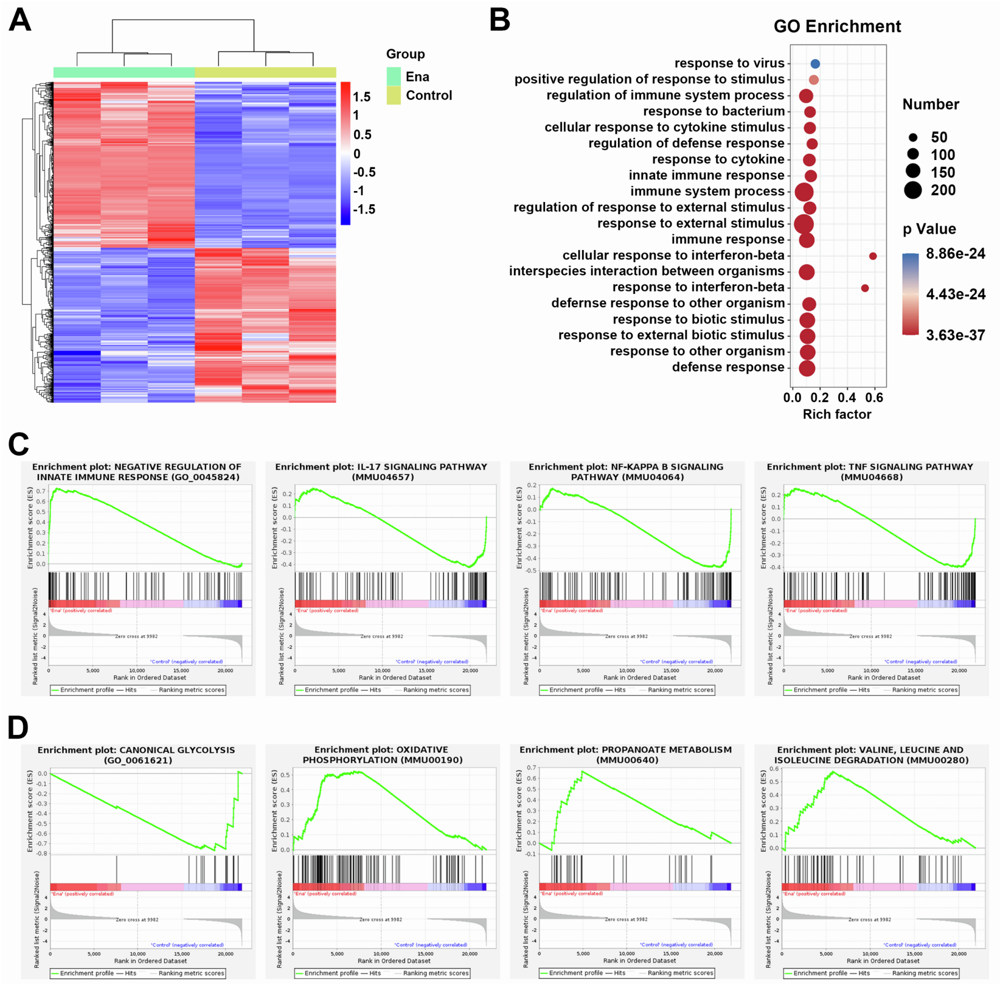

Figure S2. High-throughput RNA sequencing performed on AGE-stimulated BMDNs with or without Ena treatment. **Related to Figure 3.**

(A) Clustering analysis plot.

(B) Top 20 GO terms enriched by differentially expressed genes.

(C) Pathways involved in immune regulation shown by GESA.

(D) GESA revealing metabolic pathway including glycolysis, oxidative phosphorylation, propanoate metabolism as well as degradation of valine, leucine, and isoleucine.

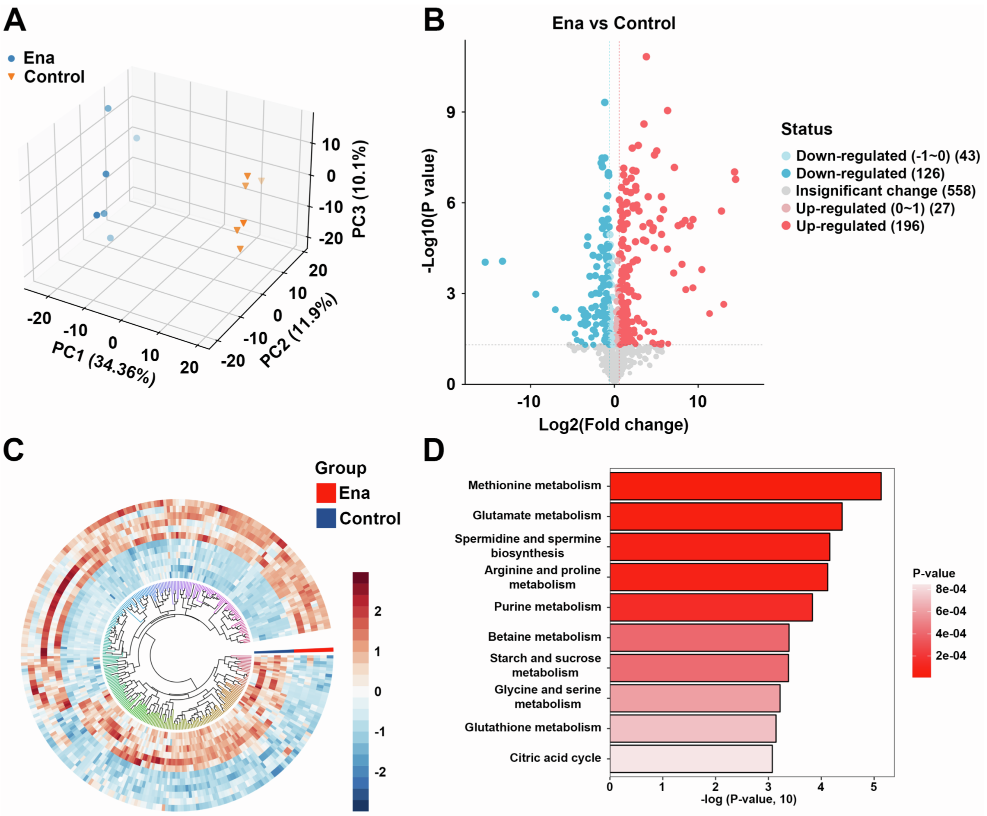

Figure S3. Metabolomics analysis performed on AGE-stimulated BMDNs with or without Ena treatment. Related to Figure 3.

(A) Visualization of PCA result.

(B) Volcano plot.

(C) Hierarchical clustering of differentially accumulated metabolites after the treatment of Ena.

(D) Small Molecule Pathway Database analysis showing significant pathways enriched by differentially accumulated metabolites.

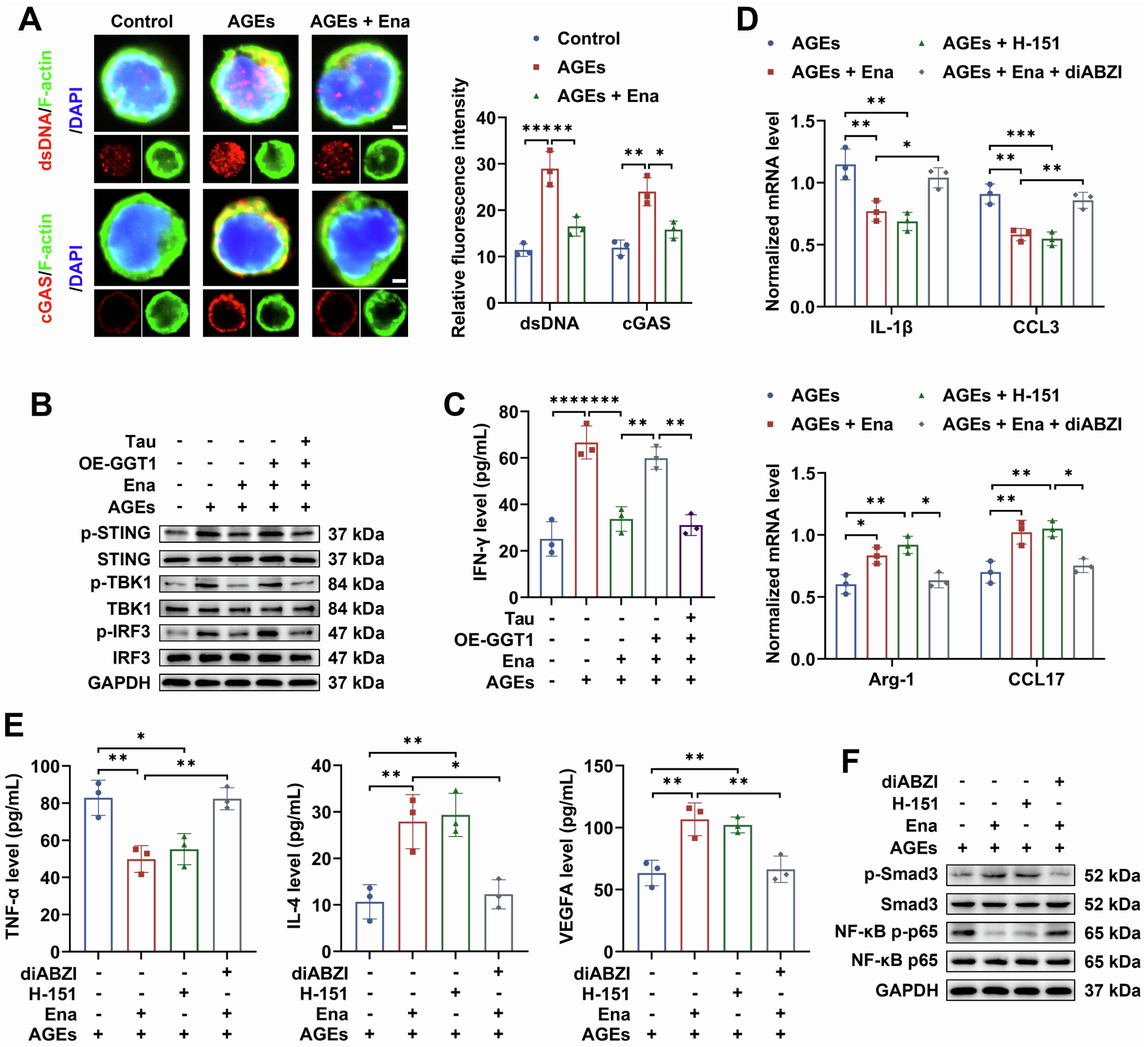

Figure S4. Ena facilitated phenotype repolarization via taurine-dependent cGAS-STING pathway inhibition. Related to Figure 4.

(A) Immunofluorescence staining analysis of dsDNA and cGAS in BMDNs with different treatments. Scale bar: 2 μm; n = 3 independent experiments.

(B) Western blot analysis of STING, TBK1 and IRF3 activity in neutrophils with indicated treatments; n = 3 independent experiments.

(C) Levels of IFN-γ released by neutrophils as measured by ELISA reagent. n = 3 independent experiments.

(D) qRT-PCR was employed to measure the expression of *il1b*, *ccl3*, *arg1* and *ccl17* in neutrophils. n = 3 independent experiments.

(E) Levels of TNF-α, IL-4 and VEGFA generated by neutrophils were detected by ELISA kits. n = 3 independent experiments.

(F) Western blot was adopted to determine the activities of Smad3 and NF-κB p65 in neutrophils. n = 3 independent experiments.

Data were shown as mean ± standard deviation (S.D.) from biological replicates and statistical comparisons were performed using one-way ANOVA followed by Tukey's multiple comparisons test in (A-F). $*P < 0.05$, $**P < 0.01$, $***P < 0.001$, $****P < 0.0001$.

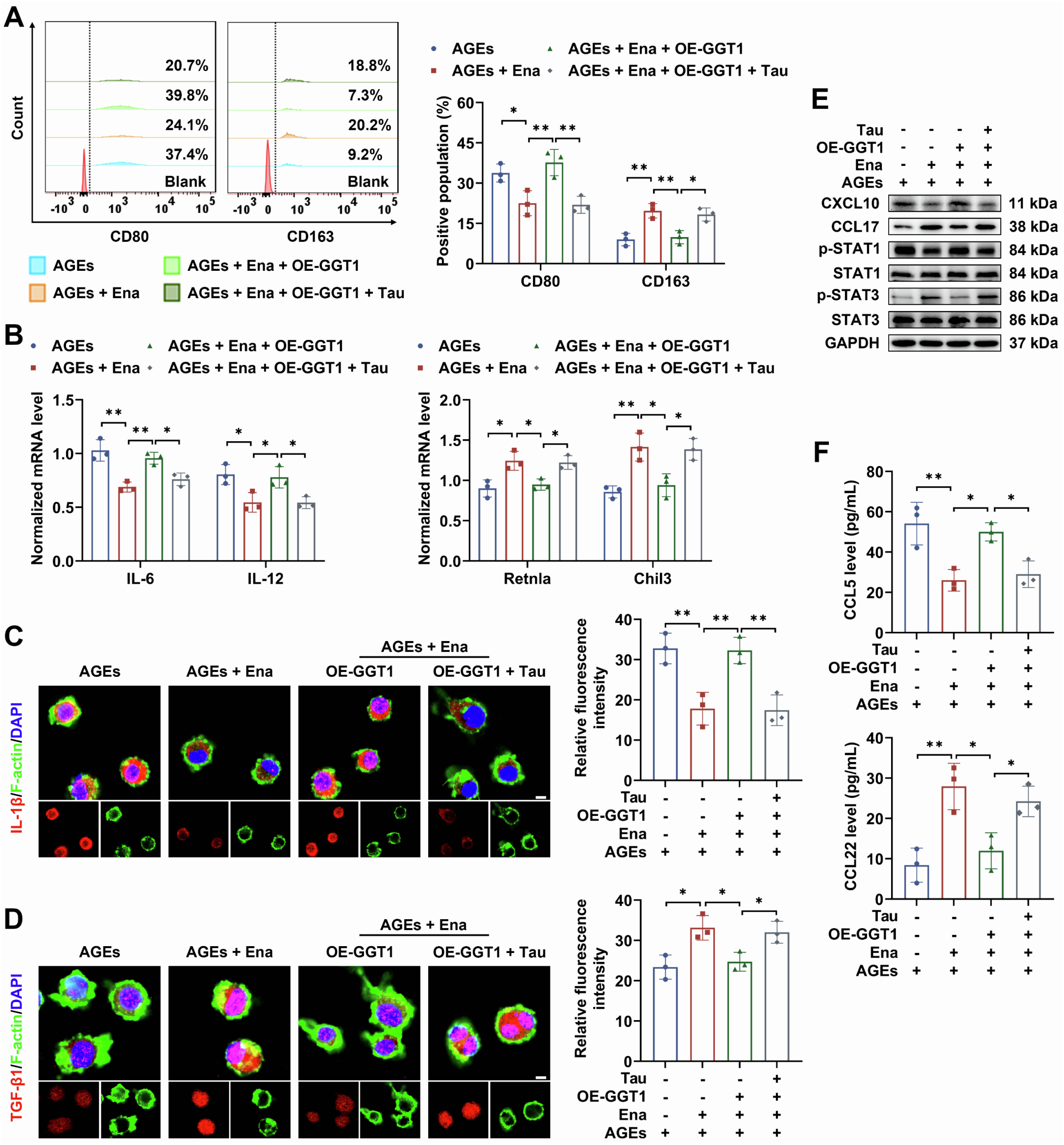

Figure S5. Regulator roles produced by Ena-treated neutrophils in the inflammation alleviation of macrophages. Related to Figure 4.

(A) Population of CD80+ or CD163+ BMDMs as evaluated by flow cytometry. n = 3 independent experiments.
(B) mRNA levels of *il6*, *il12*, *retnla* and *chil3* in BMDMs incubated with neutrophils pretreated by different approaches as measured by qRT-PCR. n = 3 independent experiments.
(C and D) Representative immunofluorescence staining of IL-1β and TGF-β1 in BMDMs affected by neutrophils with indicated preconditioning. Scale bar: 5 μm; n = 3 independent experiments.
(E) Western blot analysis of CXCL10, CCL17, STAT1 and STAT3 in macrophages. n = 3 independent experiments.
(F) Levels of CCL5 and CCL22 in BMDMs as measured by ELISA kits. n = 3 independent experiments.
Data were shown as mean ± standard deviation (S.D.) from biological replicates and statistical comparisons were performed using one-way ANOVA followed by Tukey's multiple comparisons test in (A-F). $^{*}P < 0.05$, $^{**}P < 0.01$.

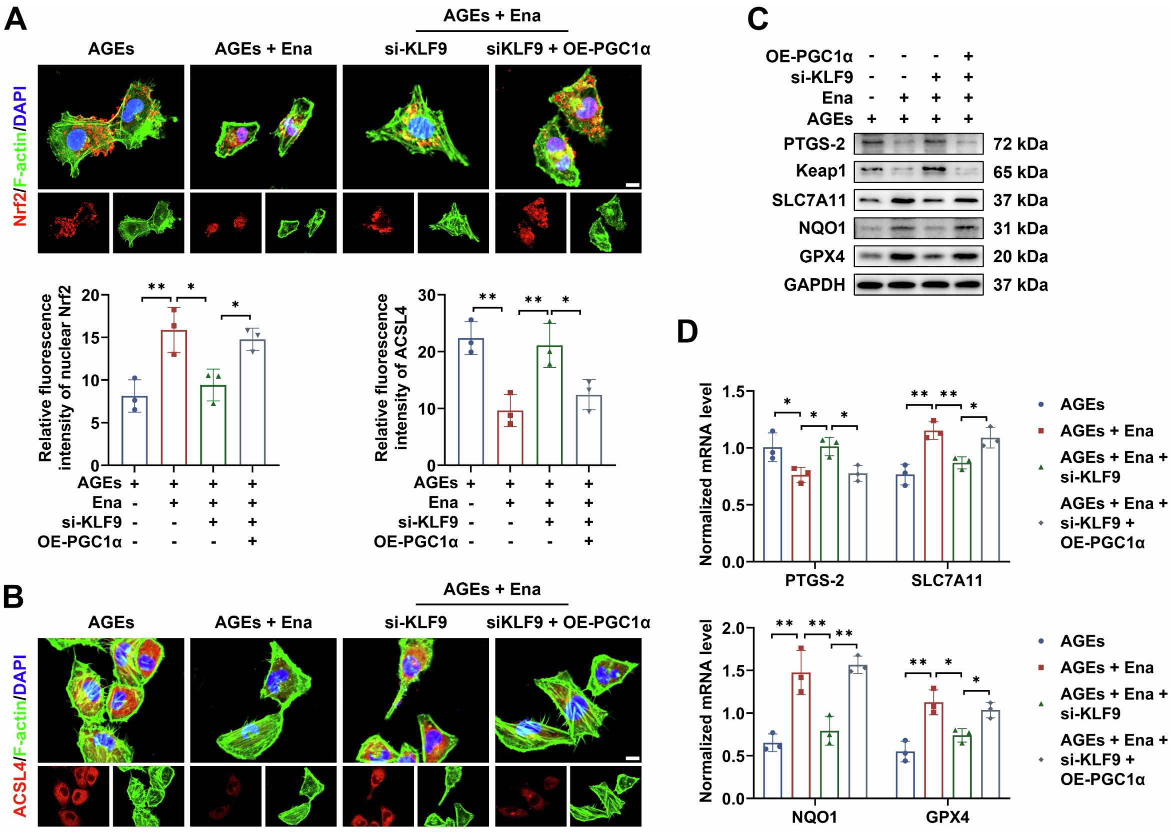

Figure S6. Mechanisms by which Ena-treated neutrophils repressed HUVEC ferroptosis. Related to Figure 5.

(A and B) Immunofluorescence analysis was employed to detect Nrf2 and ACSL4 expression in pretreated HUVECs co-cultured with AGEs and Ena-treated neutrophils. Scale bar: 10 μm; n = 3 independent experiments.

(C) Western blot analysis of PTGS-2, Keap1, SLC7A11, NQO1 and GPX4 in HUVECs. n = 3 independent experiments.

(D) mRNA levels of *ptgs2*, *slc7a11*, *nqo1* and *gpx4* in HUVECs measured by qRT-PCR. n = 3 independent experiments.

Data were shown as mean ± standard deviation (S.D.) from biological replicates and statistical comparisons were performed using one-way ANOVA followed by Tukey's multiple comparisons test in (A-D). $*P < 0.05$, $**P < 0.01$.

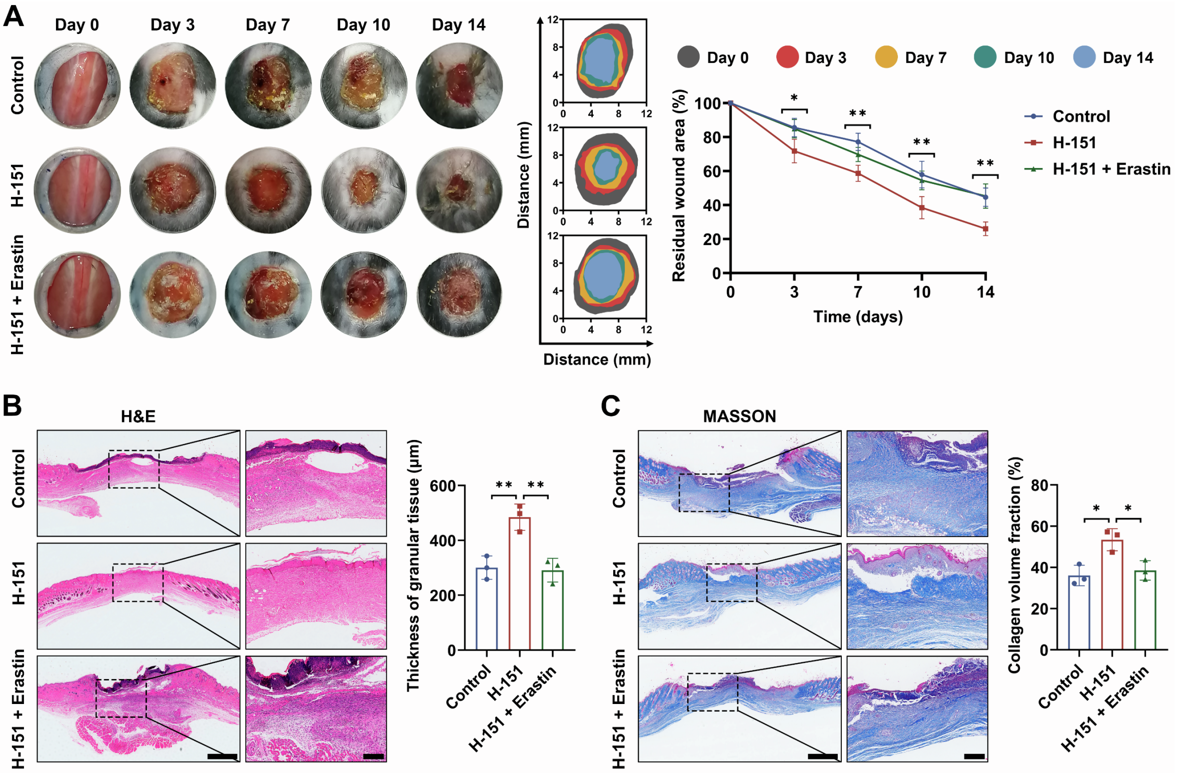

Figure S7. Effect of STING-ferroptosis axis on the healing process of diabetic wounds. Related to Figure 5.

(A) Representative images of wound area during the healing course. n = 4 mice per group.

(B) Histological analysis via H&E staining. Scale bar: 1 mm (left) and 250 μm (right); n = 3 biologically independent samples.

(C) Tissue collagen content visualized by Masson's trichrome staining. Scale bar: 1 mm (left) and 250 μm (right); n = 3 biologically independent samples.

Data were shown as mean ± standard deviation (S.D.) from biological replicates and statistical comparisons were performed using one-way ANOVA followed by Tukey's multiple comparisons test in (A-C). $*P < 0.05$, $**P < 0.01$.

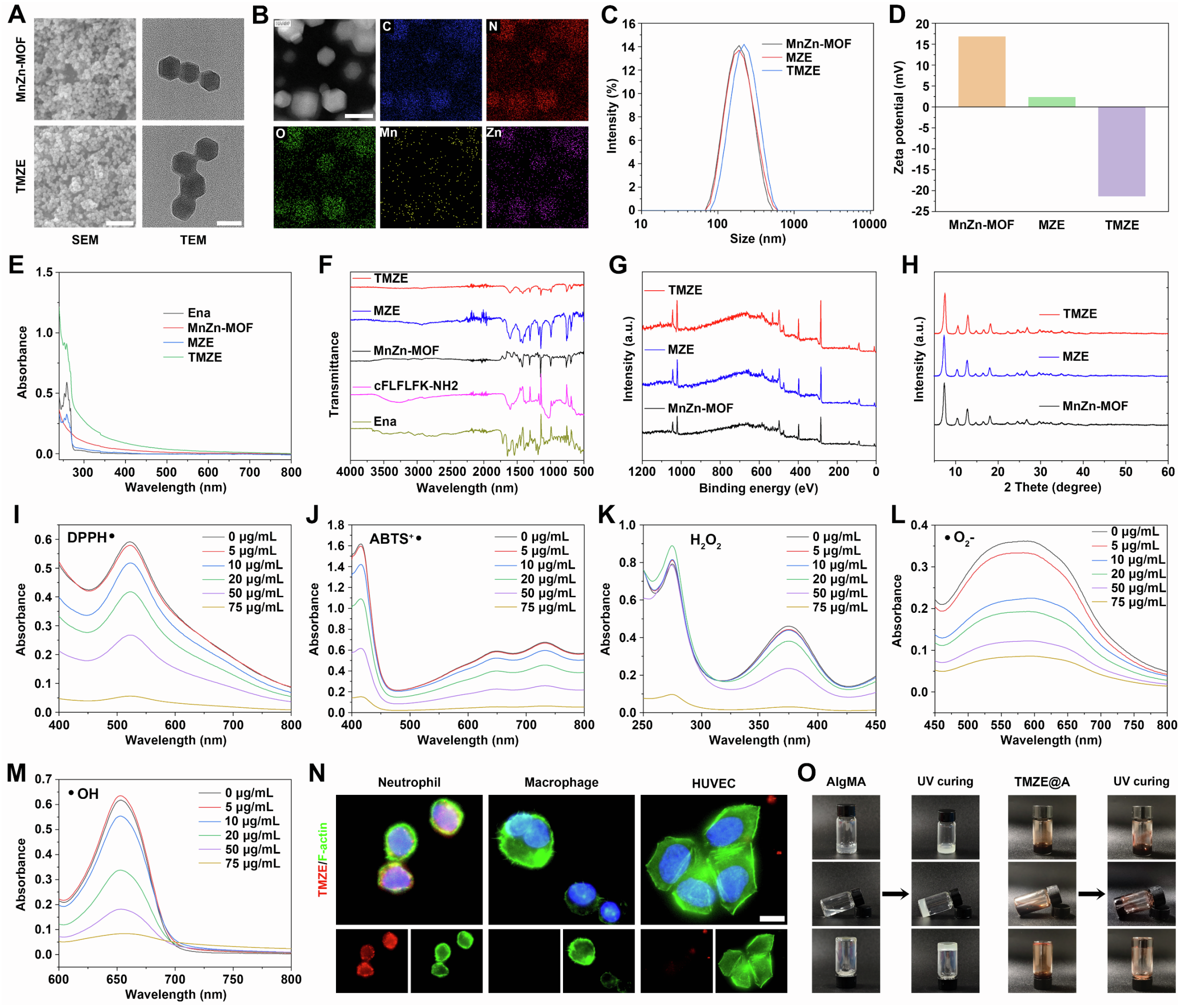

Figure S8. Manifestation of TMZE nanoparticles. Related to Figure 6.

(A) SEM and TEM were employed to disclose the appearance of MnZn-MOF and TMZE. Scale bar: 500 nm (SEM) and 50 nm (TEM); n = 3 independent experiments.

(B) Composite images were visualized by elemental mapping. Scale bar: 50 nm. n = 3 independent experiments.

(C and D) Particle size distribution and zeta potential of MnZn-MOF, MZE and TMZE. n = 3 independent experiments.

(E and F) UV-vis absorption spectra and Fourier transform infrared spectroscopy (FTIR) of each sample. n = 3 independent experiments.

(G and H) XPS spectra and powder XRD pattern of MnZn-MOF, MZE and TMZE. n = 3 independent experiments.

(L-M) Radical-eliminating activities of varied concentrations of TMZE for DPPH•, ABTS$^+$•, H_2O_2, •O_2^- and •OH as measured using UV-vis absorbance spectra. n = 3 independent experiments.

(N) Fluorescence staining was used to detect the uptake capacity of TMZE by multiple cells. Scale bar: 10 μm; n = 3 independent experiments.

(O) UV-curing property of AlgMA-based hydrogel. n = 3 independent experiments.

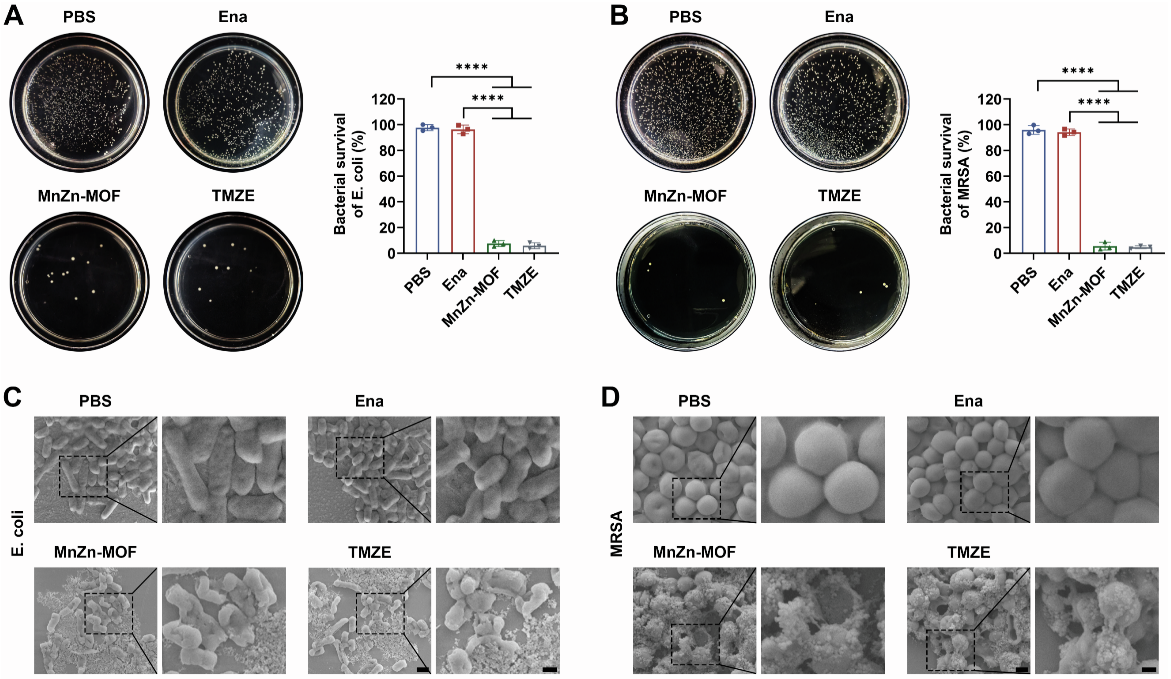

Figure S9. Antibacterial abilities of the drug carrier. Related to Figure 6.
(A and B) Bacteria-killing properties of MnZn-MOF and TMZE determined by the colony count method. n = 3 independent experiments.
(C and D) Morphology of E. coli and MRSA detected by SEM. Scale bar: 500 nm and 250 nm; n = 3 independent experiments.
Data were shown as mean ± standard deviation (S.D.) from biological replicates and statistical comparisons were performed using one-way ANOVA followed by Tukey's multiple comparisons test in (A and B). ****$P < 0.0001$.

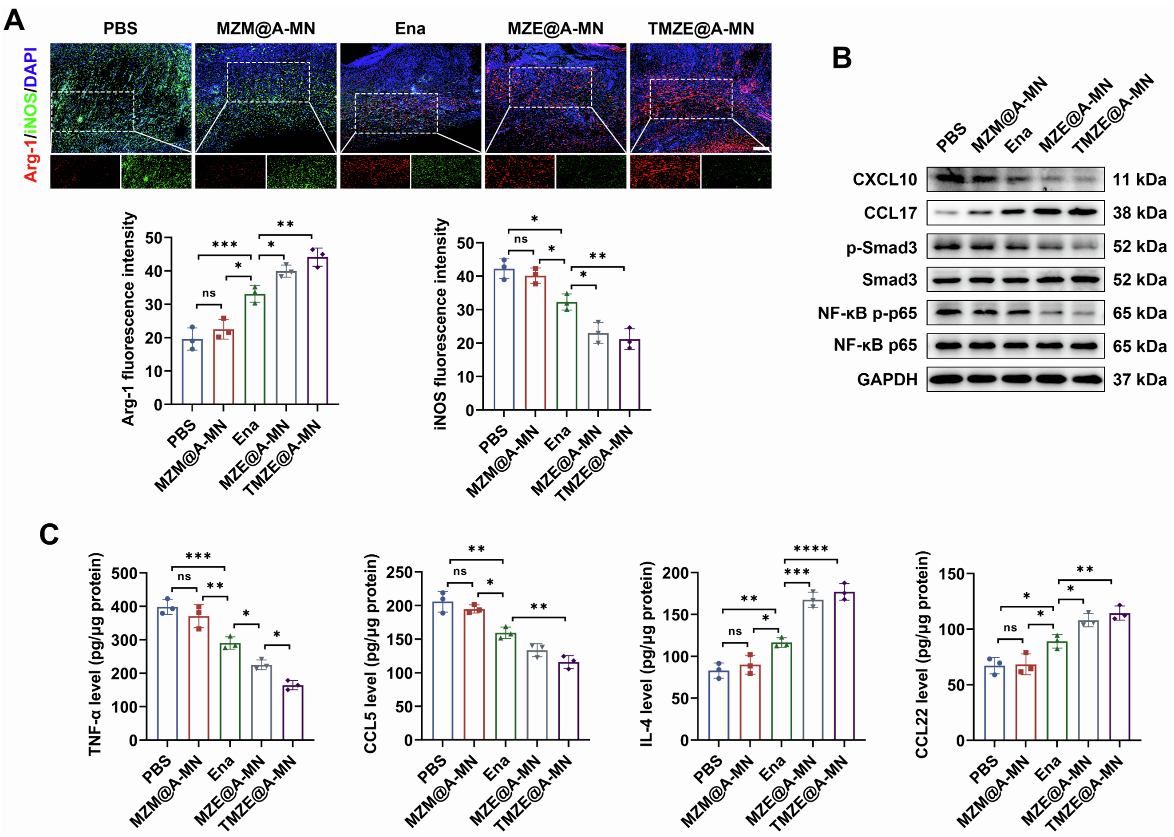

Figure S10. Effects of TMZE@A-MN on repressing inflammation responses of diabetic mouse wound tissues. Related to Figure 7.

(A) Levels of Arg-1 and iNOS in the wound area as measured using immunofluorescence staining. Scale bar: 200 μm; n = 3 biologically independent samples.

(B) Western blot detection of CXCL10, CCL17, Smad3 and NF-κB in wound tissues with different treatments. n = 3 biologically independent samples.

(C) Contents of TNF-α, CCL5, IL-4 and CCL22 in skin wounds as measured by ELISA kits. n = 3 biologically independent samples.

Data were shown as mean ± standard deviation (S.D.) from biological replicates and statistical comparisons were performed using one-way ANOVA followed by Tukey's multiple comparisons test in (A-C). ns: no significance, *$P < 0.05$, **$P < 0.01$, ***$P < 0.001$, ****$P < 0.0001$.

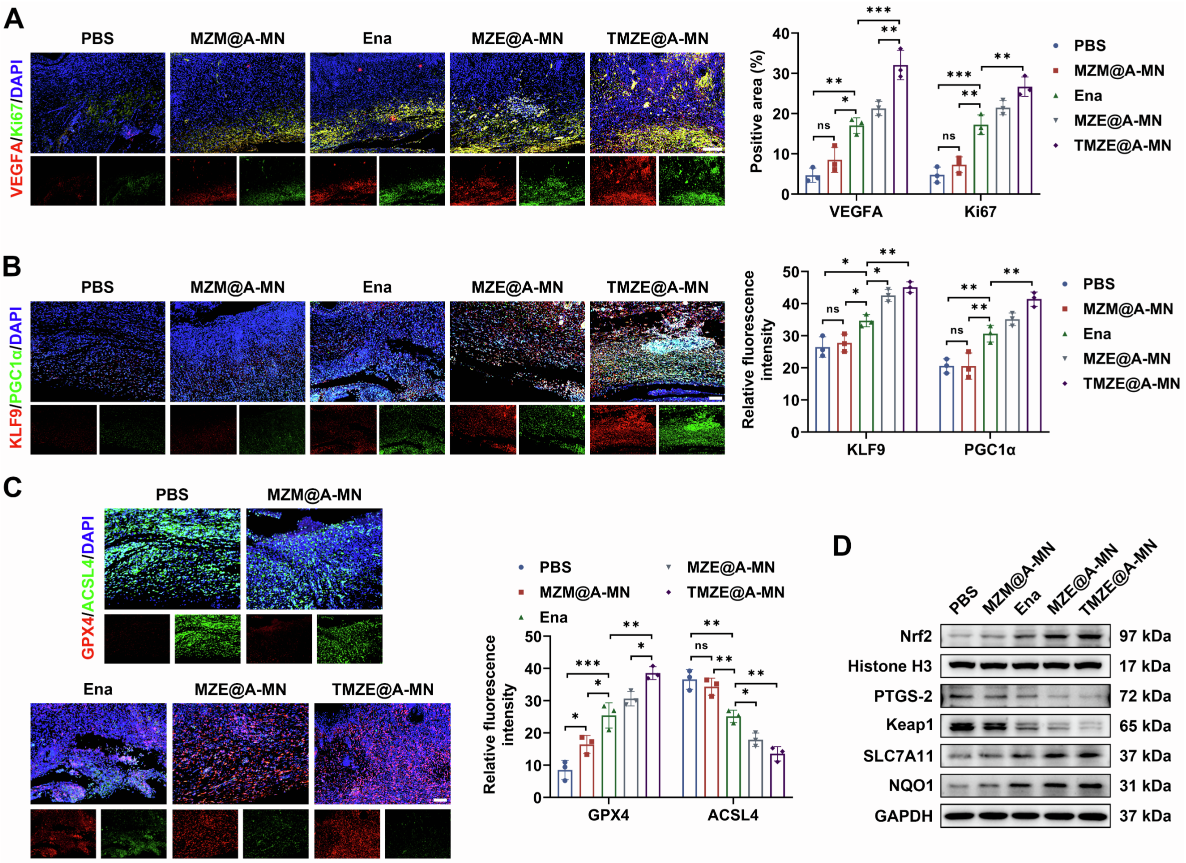

Figure S11. Regulatory roles of TMZE@A-MN in facilitating angiogenesis in the wound tissue of diabetic mice. Related to Figure 7.

(A) Expressions of VEGFA and Ki67 detected by immunofluorescence staining. Scale bar: 200 μm; n = 3 biologically independent samples.

(B) Fluorescence signals of KLF9 (red) and PGC1α (green) in the wound area. Scale bar: 100 μm; n = 3 biologically independent samples.

(C) Levels of GPX4 and ACSL4 measured by immunofluorescence staining. Scale bar: 100 μm; n = 3 biologically independent samples.

(D) Western blot analysis of Nrf2, PTGS-2, Keap1, SLC7A11 and NQO1 in the wound tissues with different treatments. n = 3 biologically independent samples.

Data were shown as mean ± standard deviation (S.D.) from biological replicates and statistical comparisons were performed using one-way ANOVA followed by Tukey's multiple comparisons test in (A-D). ns: no significance, *$P < 0.05$, **$P < 0.01$, ***$P < 0.001$.

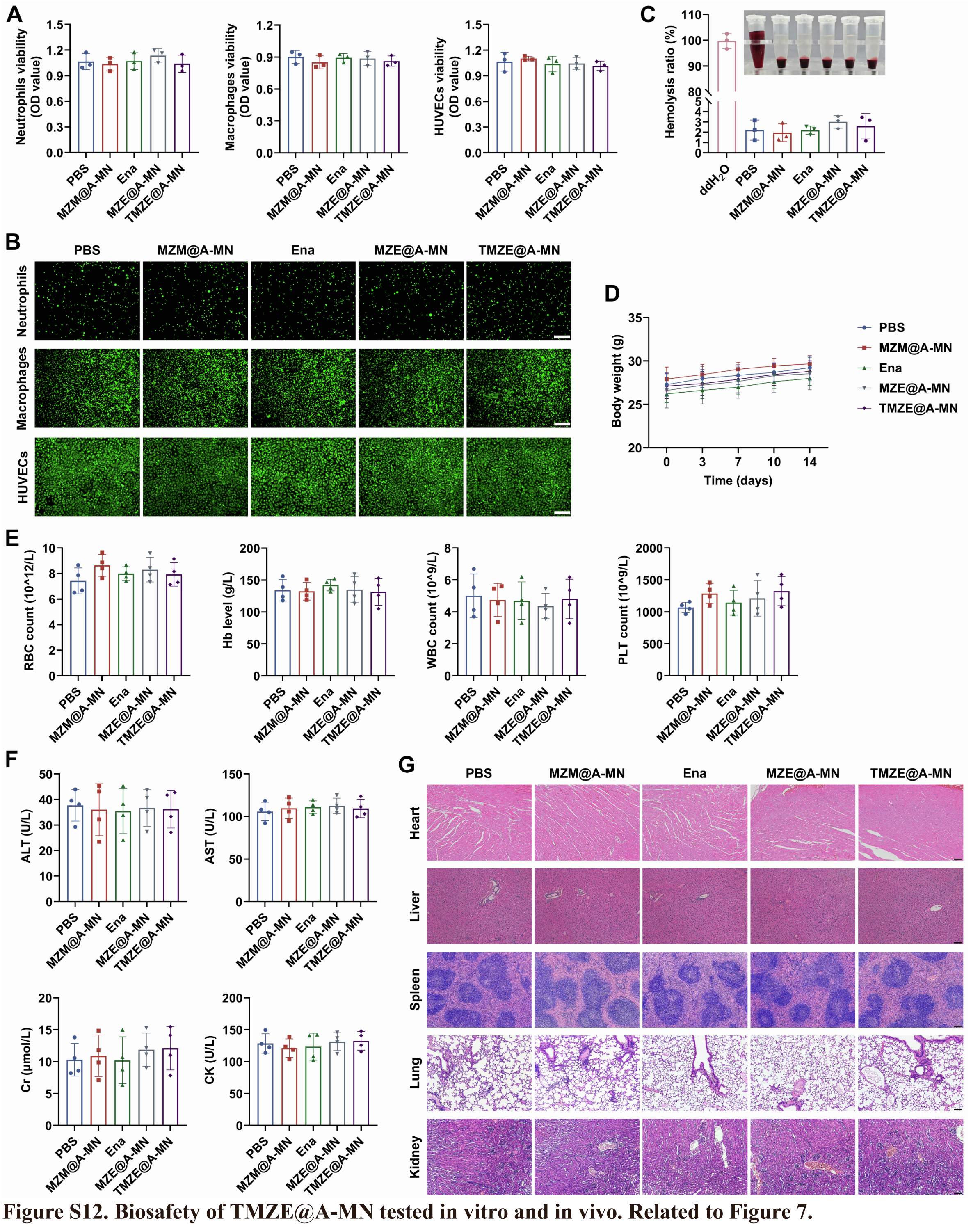

Figure S12. Biosafety of TMZE@A-MN tested in vitro and in vivo. Related to Figure 7.

(A and B) Viability of BMDNs, BMDMs and HUVECs as measured by CCK-8 assay and calcein/PI staining after incubation with different agents. Scale bar: 200 μm; n = 3 independent experiments.

(C) Results of hemolysis test. n = 3 independent experiments.

(D) Body weight changes of mice in each group. n = 4 mice per group.

(E) Main blood routine parameters as detected after mice were sacrificed. n = 4 biologically independent samples.

(F) Biochemical indicators in the blood of mice in each group. n = 4 biologically independent samples.

(G) H&E staining of visceral organs in mice with different treatment modalities for 14 days. Scale bar: 100 μm; n = 4 biologically independent samples.

Data were shown as mean ± standard deviation (S.D.) from biological replicates and statistical comparisons were performed using one-way ANOVA followed by Tukey's multiple comparisons test (A-E). The statistical differences showed above were not significant.

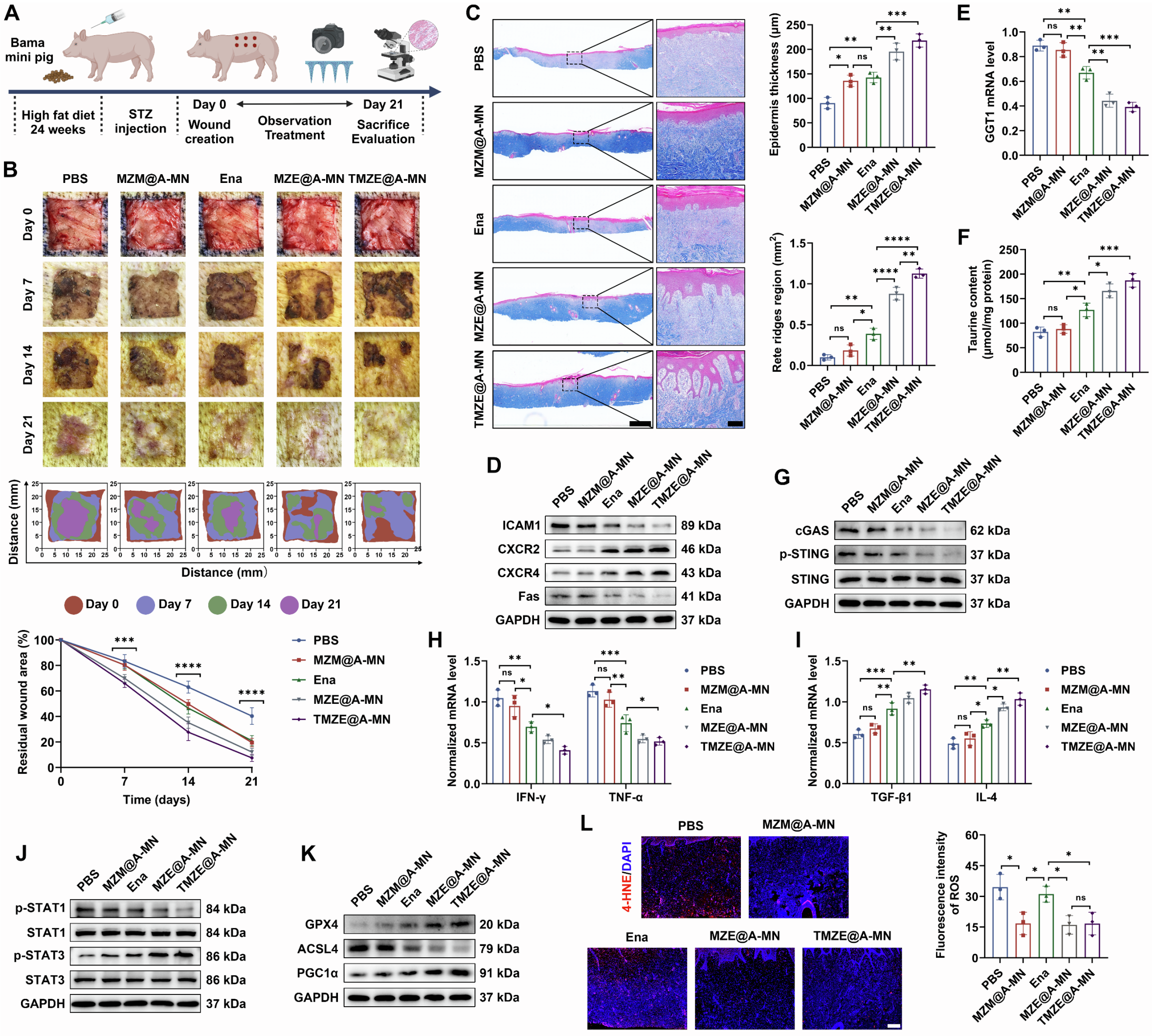

Figure S13. Beneficial roles of Ena-loaded delivery system in healing processes of porcine diabetic wounds. Related to Figure 7.

(A) Schematic illustration of the diabetic porcine wound establishment and the schedule of wound treatment *in vivo*.

(B) Representative wound images and wound closure rates of each group at days 0, 7, 14 and 21 post-surgery. n = 3 biologically independent samples.

(C) Masson's trichrome staining images of wound samples at day 21. Scale bar: 2.5 mm (left) and 250 μm (right); n = 3 biologically independent samples.

(D) Contents of ICAM1, CXCR2, CXCR4 and Fas in porcine wound tissues as determined by Western blot. n = 3 biologically independent samples.

(E and F) Quantitative analysis of GGT1 expression and taurine content within wound tissues after animals were sacrificed. n = 3 biologically independent samples.

(G-I) Activity of cGAS-STING pathway and expression of *ifnγ*, *tnfa*, *tgfb1* and *il4* in skin wounds. n = 3 biologically independent samples.

(J and K) Western blot was performed to measure the activation of STAT1 and STAT3 and the level of PGC1α, GPX4 and ACSL4 in diabetic porcine wounds. n = 3 biologically independent samples.

(L) ROS level in the wound tissue evaluated by immunofluorescence staining. Scale bar: 200 μm; n = 3 biologically independent samples.

Data were shown as mean ± standard deviation (S.D.) from biological replicates and statistical comparisons were performed using one-way ANOVA followed by Tukey's multiple comparisons test in (B-L). ns: not significant, *$P < 0.05$, **$P < 0.01$, ***$P < 0.001$, ****$P < 0.0001$.

Table S1. Demographic features of study participant. Related to STAR Methods.

Group	Patient	Age (years)	Gender	Diagnosis	Complication	Wound location	Sample size (cm^3)
DW	1	52	Male	Diabetes	Refractory ulcer	Foot	1 × 0.6 × 0.4
DW	2	59	Male	Diabetes	Refractory ulcer	Foot	0.8 × 0.8 × 0.5
DW	3	47	Male	Diabetes	Refractory ulcer	Foot	0.8 × 0.7 × 0.4
DW	4	46	Male	Diabetes	Refractory ulcer	Foot	1 × 0.8 × 0.6
DW	5	54	Male	Diabetes	Refractory ulcer	Foot	1 × 0.7 × 0.5
DW	6	63	Male	Diabetes	Refractory ulcer	Foot	0.9 × 0.6 × 0.5
AW	7	54	Male	Lower limb trauma	N/A	Foot	1 × 0.7 × 0.3
AW	8	50	Male	Lower limb trauma	N/A	Foot	0.9 × 0.6 × 0.4
AW	9	65	Male	Lower limb trauma	N/A	Foot	0.9 × 0.5 × 0.5
AW	10	49	Male	Lower limb trauma	N/A	Foot	1 × 0.7 × 0.5
AW	11	61	Male	Lower limb trauma	N/A	Foot	0.9 × 0.4 × 0.4
AW	12	51	Male	Lower limb trauma	N/A	Foot	0.7 × 0.6 × 0.5

Table S2. Sequences for siRNA design. Related to STAR Methods.

siRNA	Sense strand (5' to 3')	Antisense strand (5' to 3')
ZNF460	GGAUGGAGAUGAUAUUGUAGC	UACAAUAUCAUCUCCAUCCUA
GGT1	GCGGGAUCCUGUUCAAUAAUG	UUAUUGAACAGGAUCCCGCUG
KLF9	GGUUCAAAUAGGACUACUAGA	UAGUAGUCCUAUUUGAACCAU

Table S3. Primer sequences for ChIP. Related to STAR Methods.

Fragment	Forward (5' to 3')	Reverse (5' to 3')
RE1	TTTGAGACAGTCTCGCTCCG	AAATTAGTCAGGCGTGGTGG
RE2	TGAAGGAGGTAGGTGCTCTG	TGAAGGAGGTAGGTGCTCTG

Table S4. Primer sequences for qRT-PCR. Related to STAR Methods.

Gene	Forward (5' to 3')	Reverse (5' to 3')
**Mus musculus**		
IL-1β	TGCCACCTTTTGACAGTGATG	AAGGTCCACGGGAAAGACAC
CCL3	GTAGCCACATCGAGGGACTC	GATGGGGGTTGAGGAACGTG
Arg-1	AGCCAGGGACTGACTACCTT	TTGGGAGGAGAAGGCGTTTG
CCL17	AATGTAGGCCGAGAGTGCTG	TGCCCTGGACAGTCAGAAAC
IL-6	CCCCAATTTCCAATGCTCTCC	CGCACTAGGTTTGCCGAGTA
IL-12	GCCACCTACTCCCTTGGATCT	GACTGGCTAAGACACCTGGC
Retnla	GGGATGACTGCTACTGGGTG	TCAACGAGTAAGCACAGGCA
Chil3	TGTACCAGCTGGGAAGAAACAA	TGAGAGCAAGAAACAAGCATGG
**Homo sapiens**		
IL-1β	AGCTGACTGTCCTGGCTGAT	AGCTGACTGTCCTGGCTGAT
CCL3	ATTCCGTCACCTGCTCAGAA	GTCACACGCATGTTCCCAAG
Arg-1	GTCTGTGGGAAAAGCAAGCG	CACCAGGCTGATTCTTCCGT
CCL17	ATTCAAAACCAGGGTGTCTCC	CTCTTGTTGTTGGGGTCCGA
CDO	GGTGGGTCTCTTGCTGTCTC	TCAGCACTTCGGTCTGTTCC
CSAD	CTCAGAGCGGGTGAGCTTG	CACGTGTGGAGGCTAGTGTT
FMO1	TTGTTAGAGCAGCCAAGGGT	TTCGGTGAATCTCCACAGCC
BAAT	CAGGAGAGGGTCTCTTCCCA	AGAAAGTTGGCAGCCTCCTC
PTGS-2	TTGCATTCTTTGCCCAGCAC	ACCGTAGATGCTCAGGGACT
SLC7A11	TGGAACGAGGAGGTGGAGAA	TGGTGGACACAACAGGCTTT
NQO1	TGCTTACACTTACGCTGCCAT	CCAGTGGTGATGGAAAGCAC
GPX4	ATTGGTCGGCTGGACGAGG	TCGATGTCCTTGGCGGAAAA
**Sus scrofa**		
GGT1	GAAGGAGGACTGTCAGTGGC	CTCAATGCTGGGCTGGAAGA
IFN-γ	AGCTTTTCAGCTTTGCGTGA	TGCTCCTTTGAATGGCCTGG
TNF-α	CTGTAGGTTGCTCCCACCTG	ACACGCTTTTATTTCTCGCCA
TGF-β1	ACCTGCAAGACCATCGACAT	AGAGCAATACAGGTTCCGGC
IL-4	CTCCCAACTGATCCCAACCC	TGCACGAGTTCTTTCTCGCT
